# Discovery of Dipyridamole Analogues with Enhanced Metabolic Stability for the Treatment of Idiopathic Pulmonary Fibrosis

**DOI:** 10.3390/molecules27113452

**Published:** 2022-05-26

**Authors:** Meng-Xing Huang, Yan-Quan Chen, Run-Duo Liu, Yue Huang, Chen Zhang

**Affiliations:** 1 School of Pharmaceutical Sciences, Sun Yat-Sen University, Guangzhou 510006, China; huangmx28@mail2.sysu.edu.cn (M.-X.H.); chenyq326@mail2.sysu.edu.cn (Y.-Q.C.); liurd3@mail2.sysu.edu.cn (R.-D.L.); huangy266@mail2.sysu.edu.cn (Y.H.); 2 School of Chemistry and Chemical Engineering, Guangdong Pharmaceutical University, Zhongshan 528458, China

**Keywords:** dipyridamole, phosphodiesterase, metabolic stability, pulmonary fibrosis, molecular docking

## Abstract

Dipyridamole, apart from its well-known antiplatelet and phosphodiesterase inhibitory activities, is a promising old drug for the treatment of pulmonary fibrosis. However, dipyridamole shows poor pharmacokinetic properties with a half-life (T_1/2_) of 7 min in rat liver microsomes (RLM). To improve the metabolic stability of dipyridamole, a series of pyrimidopyrimidine derivatives have been designed with the assistance of molecular docking. Among all the twenty-four synthesized compounds, compound **(*S*)-4h** showed outstanding metabolic stability (T_1/2_ = 67 min) in RLM, with an IC_50_ of 332 nM against PDE5. Furthermore, some interesting structure–activity relationships (SAR) were explained with the assistance of molecular docking.

## 1. Introduction

Idiopathic pulmonary fibrosis (IPF) is a chronic, progressive and fibrotic interstitial lung disease with unknown etiology [1,2]. The majority of IPF patients are middle-aged or elderly [3]. Once diagnosed, the average survival of patients is only 3–5 years [4,5]. Clinical manifestations are early acute lower respiratory tract infection, leading to pulmonary hypertension, progressive dyspnea and, eventually, death, due to respiratory failure in most cases [6,7]. The marketed drugs pirfenidone and nintedanib, approved for IPF, can slow down the disease progression but not cure it [8,9]. Currently, the COVID-19 epidemic remains relatively grim, and extensive data from the COVID-19 pandemic indicate that a large number of pulmonary fibrosis sequelae are likely to occur after SARS-CoV-2 infection [10]. Given that hundreds of millions of people worldwide are suffering from COVID-19, it is likely to cause pulmonary fibrosis sequelae among the infected population. Therefore, novel and effective therapeutic approaches for IPF are in great demand.

Dipyridamole (DIP), an antiplatelet-marketed drug, is mainly used for ischemic heart disease and strokes [11]. Publications reported that DIP is a phosphodiesterase-5 (PDE5) inhibitor [12]. PDE5 belongs to a subfamily of phosphodiesterases (PDEs) and performs a variety of physiological functions by specifically hydrolyzing the second messenger cGMP. Published studies have demonstrated that activation of cGMP signaling showed good antifibrotic efficacy in various animal models of pulmonary fibrosis [13]. Sildenafil, a well-known PDE5 inhibitor, has been proven to attenuate the degree of pulmonary fibrosis, and several phase II clinical trials have been completed [14]. Most recently, Liu et al. reported that DIP showed good efficacy for the treatment of critically ill patients with COVID-19 and improved the lung fibrosis of these patients [15]. In addition, a published patent indicated that DIP significantly decreased the levels of fibrosis markers α-SMA and FN, exerting remarkable therapeutic effects in the rat model of pulmonary fibrosis induced by bleomycin and prevented myofibroblast differentiation induced by TGF-β [16]. However, the pharmacokinetic properties of DIP are poor, practically insoluble in water, which leads to poor bioavailability. Moreover, we found that the half-life (T_1/2_) of DIP (measured by Shanghai Medicilon Inc., Shanghai, China) in rat liver microsomes (RLM) is only 7 min. Therefore, it was necessary to develop novel dipyridamole analogues with improved metabolic stability. Given that, we designed and synthesized pyrimidopyrimidine+ derivatives with excellent metabolic stability.

**Figure 1:**
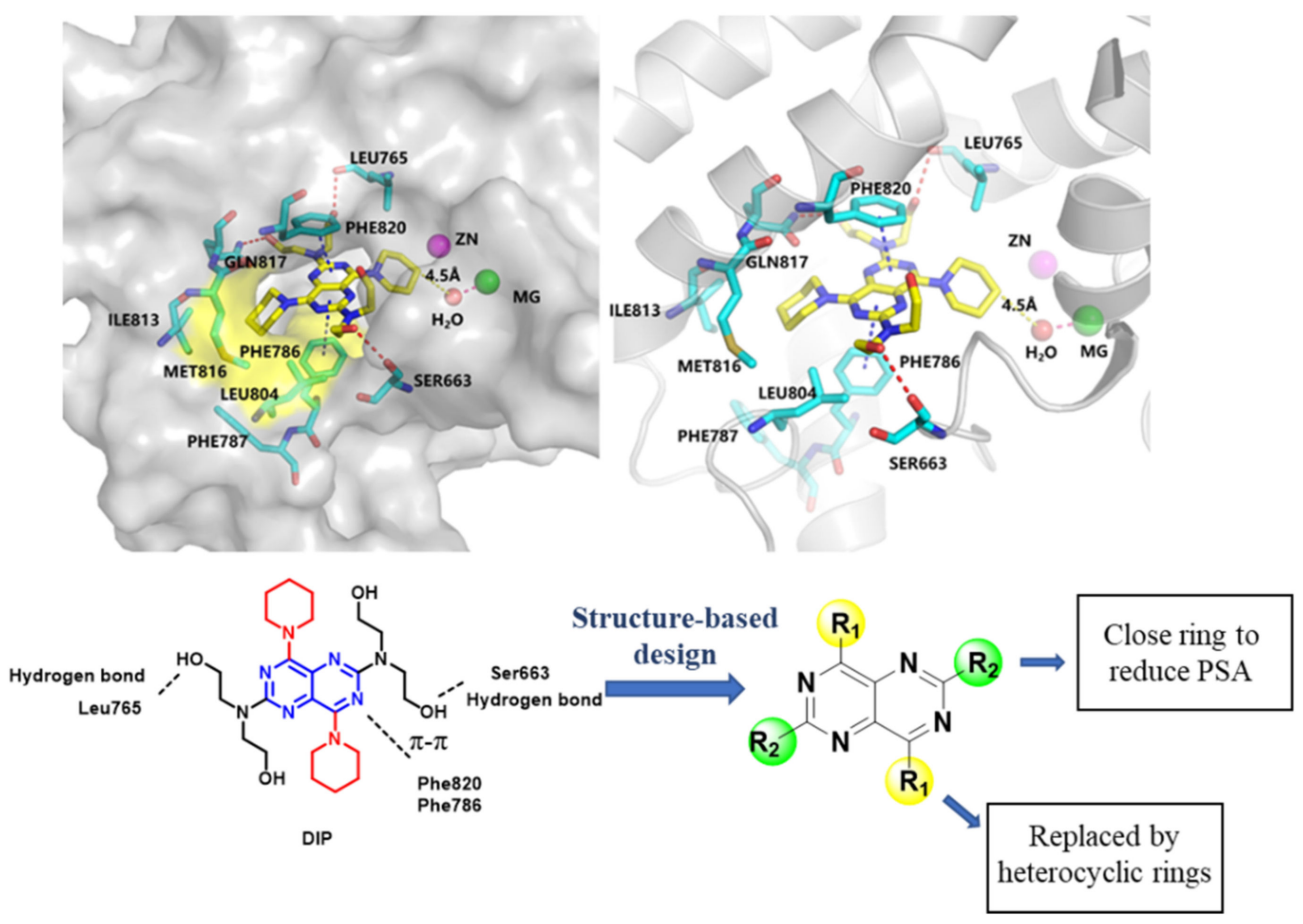
Chemical structure and putative binding pattern of dipyridamole with PDE5, according to the molecular docking and the design strategy for PDE5 inhibitors.

Based on the molecular docking pattern of DIP in PDE5 catalytic domain (see Figure 1), we found that the pyrimido[5,4-d]pyrimidine ring is anchored to the core pocket of PDE5 by forming *π*–*π* stacking with Phe820 and Phe786. For the two diethanolamine chains, one can form hydrogen bond interactions with Gln817 and Leu765, respectively, each using a hydroxyl group. The other diethanolamine chain donates a hydrogen bond to Ser663 using one of its hydroxyl groups. In particular, two hydrogen bonds with Leu765 and Ser663 have not been reported in other PDE5 inhibitors.

Our previous studies suggested that introducing certain heterocyclic rings (especially those containing fluorine atoms), other than piperidine rings, were useful for the improved metabolic stability of PDE inhibitors [17,18]. For DIP, its piperidine rings made fewer key interactions than diethanolamine chains with PDE5. Therefore, we first replaced the piperidine rings by other heterocyclic rings in order to increase the inhibitory activity as well as metabolic stability of designed compounds. Second, the strategy of closing ring was applied to reduce the number of hydroxyl groups. Since DIP possesses a total of twelve nitrogen and oxygen atoms, contributing to its high PSA value, the chains of diethanolamine on both sides were replaced by heterocyclic rings to decrease the PSA of DIP. All designed compounds were docked to PDE5 for the prediction of binding patterns and then synthesized to evaluate their inhibitory activities against the PDE5 and T_1/2_ of preferred compounds in RLMs. Furthermore, the structure–activity relationship was explained based on molecular docking results.

## 2. Results

### 2.1. Chemistry

**Scheme 1:**
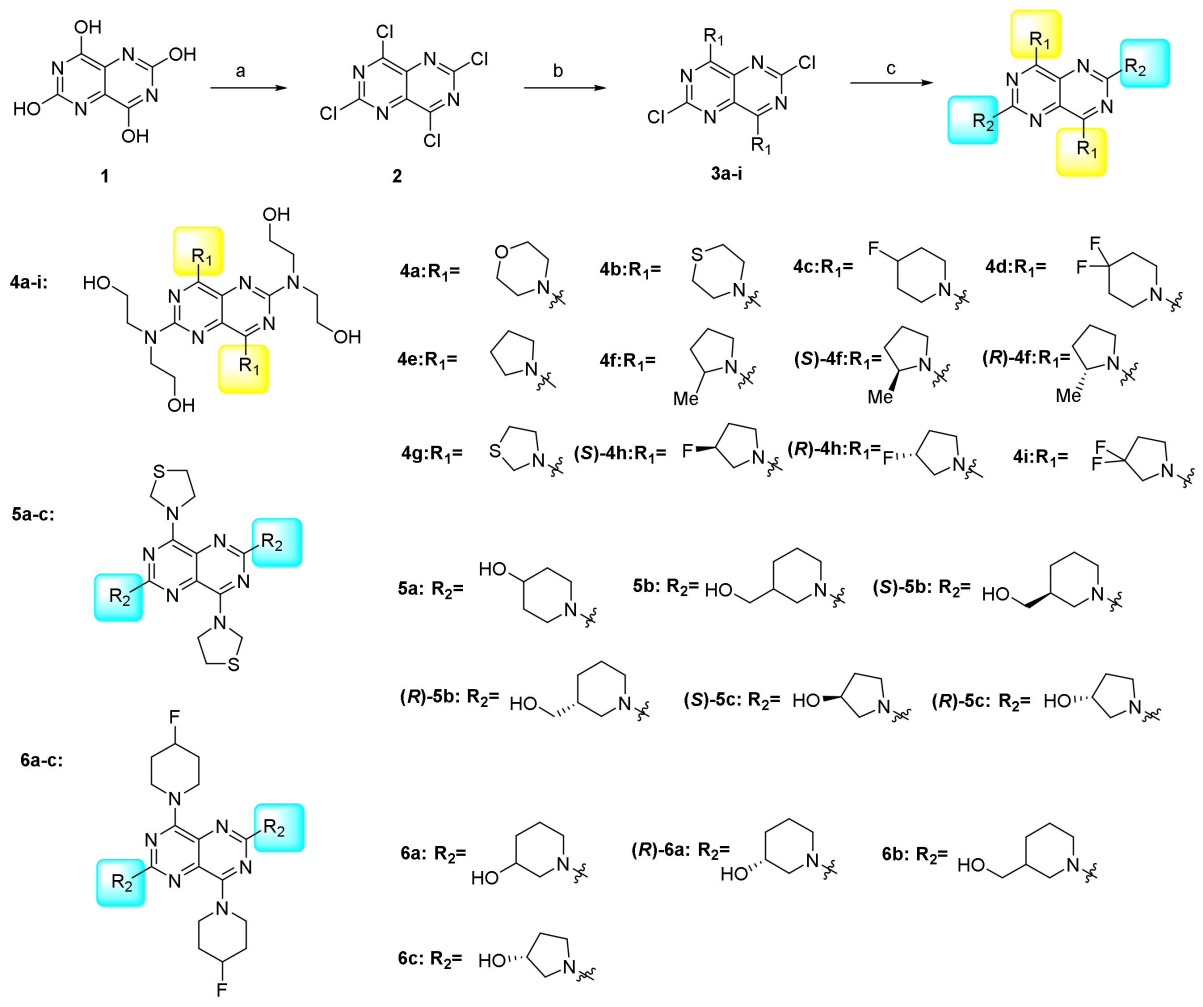
The synthetic routes of targeted compounds (**4a–i**, **5a–c**, **6a–c**).

The preparation route of the designed compounds is displayed in Scheme 1. Pyrimido[5,4-d]pyrimidine-2,4,6,8-tetraol (1) reacted with phosphorus oxychloride and phosphorus pentachloride to afford perchloropyrimido[5,4-d]pyrimidine (2). In the presence of THF, the intermediate 2 was substituted with different amines to generate corresponding intermediate 3 [19], and then reacted with diethanolamine or different amines to afford **4a–i**, **5a–c** and **6a–c** in good yields.

Reagents and conditions: (a) POCl_3_, PCl_5_, 120 °C; (b) THF, room temperature, 30 min; (c) DEA, 150 °C, overnight (**4a–i**); amine, 100 °C, overnight (**5a–c**, **6a–c**).

**Scheme 2:**
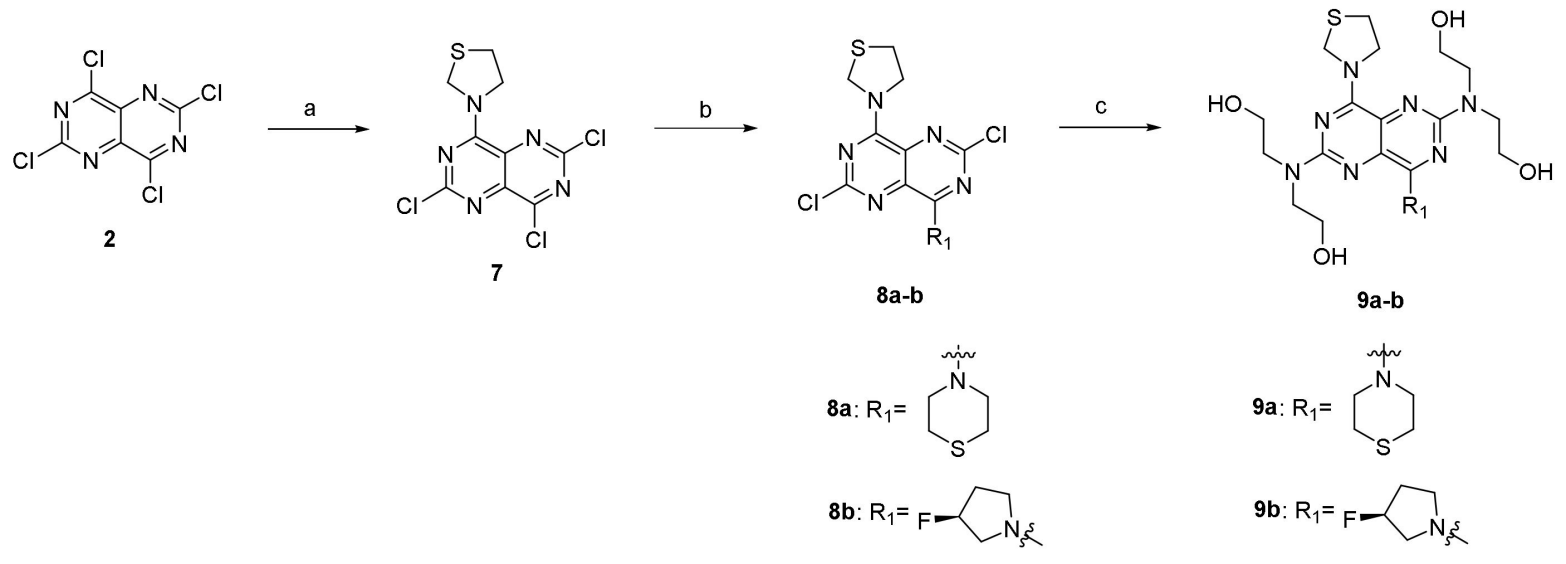
The synthetic routes of targeted compounds **9a** and **9b**. Reagents and conditions: (**a**) 1.14 equiv. thiazolidine, K_2_CO_3_, THF, −78 °C, 15 min; (**b**) 2 equiv. amine, THF, 0 °C, 40 min.; (**c**) DEA, 150 °C, overnight (**9a** and **9b**).

The preparation route of the designed compounds **9a** and **9b** is displayed in Scheme 2. Perchloropyrimido[5,4-d]pyrimidine **2** reacted with thiazolidine to afford 3-(2,6,8-trichloropyrimido[5,4-d]pyrimidin-4-yl)thiazolidine **7**. In the presence of THF, the intermediate **7** was substituted with different amines to generate corresponding intermediate **8a** and **8b**, and then reacted with diethanolamine to afford **9a** and **9b** in good yields.

### 2.2. Inhibitory Activity of PDE5 Inhibitors

The enzymatic assay of dipyridamole and its derivatives against PDE5 was performed in vitro, with sildenafil as a positive control. The inhibitory activities of the target compounds are summarized in Table 1, Table 2 and Table 3. Among those modified compounds, there were twelve compounds with the IC_50_ values below 500 nM and compound **4g** exhibited optimal inhibitory activity of 64 nM. In addition, **4b**, **4c**, **4d** and **(*S*)-4h** also showed good inhibition.

**Figure 2:**
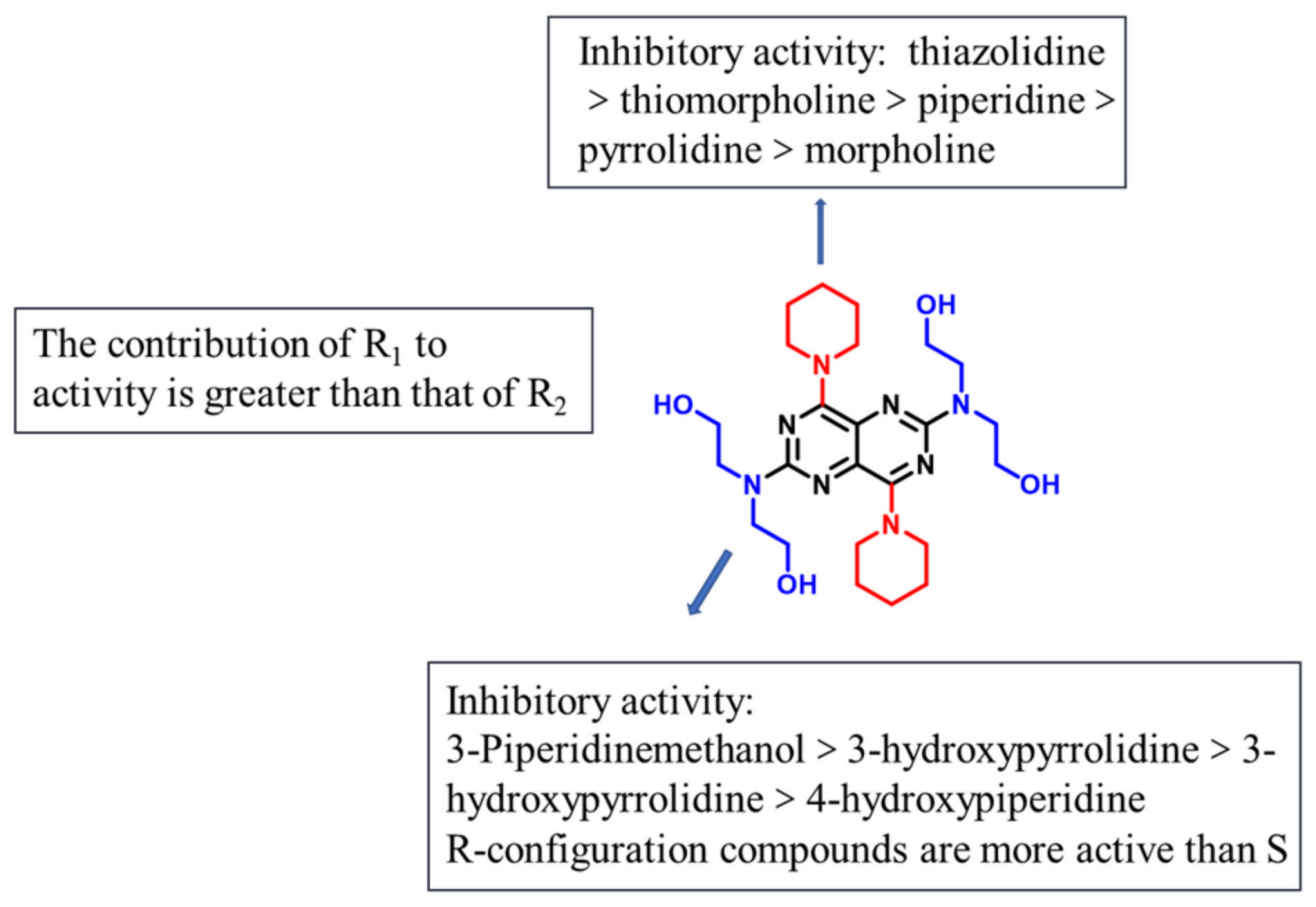
The structure–activity relationship of designed compounds.

Herein, we summarize the structure–activity relationship (see Figure 2). The first round of modifications concentrated on 4,8-positions of pyrimidopyrimidine core (R_1_ groups). Compounds **4b** and **4g** with lipophilic tetrahydrothiazole and thiomorpholine rings showed the best inhibitory activity with IC_50_ of 64 nM and 113 nM, respectively. But compound **4e** with smaller tetrahydropyrrole rings showed less than 50% inhibition at 500 nM. Compounds **4c** and **4d** containing fluorine-substituted piperidine rings exhibited a slightly decreased activity compared to DIP, with IC_50_ of 343 and 362 nM, respectively. In contrast, compounds **4h–i** containing fluorine-substituted tetrahydropyrrole rings exhibited increased activity compared to **4e**. The inhibitory activity of compound **(*S*)-4h** was slightly better than **(*R*)-4h**. Morpholine-substituted compound **4a** exhibited decreased activity compared to DIP (IC_50_ > 500 nM), suggesting that hydrophobic groups with certain volume at 4,8-positions of pyrimidopyrimidine core are advantageous to PDE5 inhibition.

In the second round of modifications, the designed compounds showed moderate activity against PDE5. Different heterocycles were used to replace the diethanolamine chains on the two representative compounds **4g** and **4c**. Although the PSA values were reduced, all modified compounds exhibited weaker inhibitory activities compared to **4g** and **4c**.

Considering that **4g** and **4b** had higher inhibitory activity against PDE5 and **(*S*)-4h** exhibited outstanding metabolic stability, structural modifications were also performed, in which R_1_ substitutions from **4g**, **4b** and **(*S*)-4h** were combined. Both of the designed compounds **9a** and **9b** showed high activity with IC_50_ of 100 nM and 94 nM, respectively (Table 3).

### 2.3. Metabolic Stability of Typical Compounds in Rat Liver Microsomes

We tested T_1/2_ in the RLM of dipyridamole and representative synthesized compounds with comparable activity to dipyridamole (Table 4). The test method was included in Supplementary Materials. Results showed that the metabolic stability of compounds **4b, 4g, 9a** and **9b** with sulfur-containing heterocycles were as poor as DIP, while compounds **4c**, **4d** and **(*S*)-4h** with fluorine-containing heterocycles had remarkable enhancement in metabolic stability [20]. Considering that the introduction of fluorine into a molecule could productively influence pKa and pharmacokinetic properties [21], the improved metabolic stability of **4c** and **4d** (with fluoro-substituted piperidine rings) might attribute to reduced basicity of the nitrogen atoms in piperidine rings compared to DIP. The replacement of the 4-fluoropiperidin-1-yl group with the 3-fluoropyrrolidin-1-yl one changed the lipophilicity profile, which further led to the improved metabolic stability of **(*S*)-4h** in comparison to **4b**. Among them, compound **(*S*)-4h** demonstrated optimal metabolic stability, with the T_1/2_ reaching 67 min.

### 2.4. Molecular Docking

**Figure 3:**
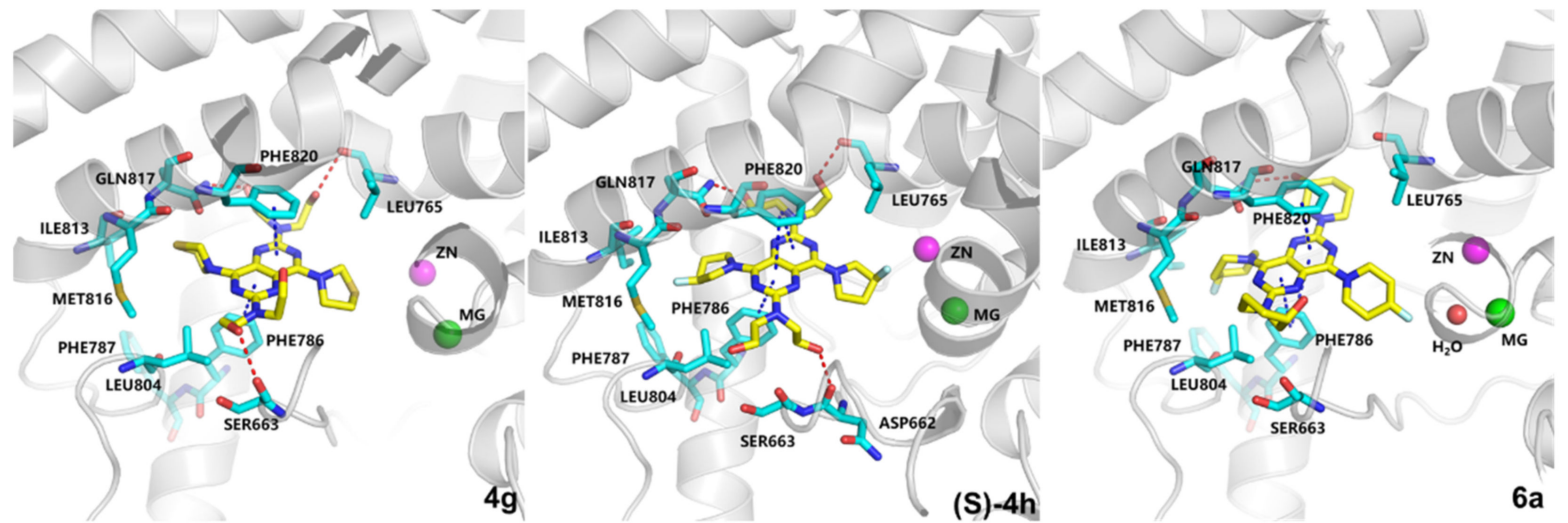
Putative binding modes of compounds **4g**, **(*S*)-4h** and **6a** with PDE5 in molecular docking studies.

The structure–activity relationship was further illustrated by molecular docking results for designed compounds (see Figure 3). Observed from the binding patterns of **4g** (with optimal inhibitory activity against PDE5), tetrahydrothiazole groups at 4,8-positions of the pyrimidopyrimidine core extended into and interacted with the hydrophobic pocket near Gln817, which is composed of Ile813, Met816, Phe787, Leu804 and Phe786. Therefore, substituents with hydrophobic properties at these positions should be preferable. The binding pattern of **(*S*)-4h** (with optimal metabolic stability) to PDE5 indicated that introduction of fluorine atoms to 4,8-positions resulted in enhanced interaction with Met816 and Leu765 in comparison to **4e**, which could explain the enhanced inhibition from **4e** to **(*S*)-4h** or **4i**. However, it is worth noting that the electron-withdrawing substituents in 4,8-positions are not necessarily better in potency. The introduction of fluorine atoms to piperidine rings in DIP might produce a weak Coulomb repulsion to the conserved coordinating water in the metal ion region of PDE5, since the 4-position carbon atom of the piperidine ring in dipyridamole and the oxygen atom in the coordinating water is close. This was in accordance with decreased inhibition from DIP to **4c** and **4d**.

Binding patterns between PDE5 and compounds with piperidine or pyrrolidine rings at 2,6-positions of the pyrimidopyrimidine core revealed that a hydrogen bond originally formed with Leu765 in DIP-PDE5 complex no longer exists. As can be seen from **6a**-PDE5 complex, the bulky cycloalkane substituents would force the entire molecule to shift outside the pocket, so that **6a** and other compounds might not form hydrogen bonds with Leu765 and Ser663. This was in accordance with the fact that most compounds in the second round of design showed more decreased activity than DIP.

## 3. Materials and Methods

### 3.1. Chemistry

Reagents and materials were commercially available (Bide Pharmatech Ltd., Shanghai, China; Energy Chemical, Shanghai, China; Sigma-Aldrich, St. Louis, MO, USA; Aladdin, Shanghai, China etc.) and used directly without further purification. The NMR spectra of intermediates and target compounds were recorded on a Bruker Avance III (^1^H NMR: 400 MHz, ^13^C NMR: 101 MHz) or Bruker Ascend TM (^1^H-NMR: 500 MHz, ^13^C-NMR, 126 MHz). The high-resolution mass spectra of pyrimidopyrimidine derivatives were analyzed using a Shimadzu LCMS-IT-TOF mass spectrometer.

#### 3.1.1. General Procedure for Synthesis of Intermediate Compounds **2**

The compound **1** pyrimido[5,4-d]pyrimidine-2,4,6,8-tetraol (2g, 10 mmol) was added to a solution of PCl_5_ (12 g, 58 mmol) in POCl_3_ (99 mL, 1.07 mol). The mixture was stirred at 120 °C overnight and the extra solvent was removed under reduced pressure to give the yellow-brown residue. Ice-water was added to the mixture, yellow solids were given, filtered and washed with water until acid-free. The solids were purified by pulping with dichloromethane/methanol (20:1) to afford compound **2** as pale-yellow solids.[22] ^13^C NMR (101 MHz, CDCl_3_) *δ* 164.62, 156.68, 140.75. 

#### 3.1.2. General Procedure for Synthesis of Intermediate Compounds **3a–i**

Compound **2** (1 mmol) was dissolved in tetrahydrofuran, and different R_2_ substituted amines (4 mmol) were added to react for 30 min at room temperature to afford corresponding intermediate compounds **3a–i** in good yields [19,22]. 

4,4′-(2,6-dichloropyrimido[5,4-d]pyrimidine-4,8-diyl)dimorpholine (**3a**). White solid. Yield, 60%. ^1^H NMR (400 MHz, CDCl_3_) δ 4.37 (s, 8H), 3.90–3.74 (m, 8H).

4,4′-(2,6-dichloropyrimido[5,4-d]pyrimidine-4,8-diyl)bis(thiomorpholine) (**3b**). White solid. Yield, 58%. ^1^H NMR (400 MHz, CDCl_3_) δ 4.57 (s, 8H), 3.36–2.46 (m, 8H).

2,6-dichloro-4,8-bis(4-fluoropiperidin-1-yl)pyrimido[5,4-d]pyrimidine (**3c**). White solid. Yield, 50%. ^1^H NMR (400 MHz, CDCl_3_) δ 5.09–4.95 (m, 1H), 4.96–4.82 (m, 1H), 4.66 (s, 4H), 4.08 (s, 4H), 2.19–1.80 (m, 8H).

2,6-dichloro-4,8-bis(4,4-difluoropiperidin-1-yl)pyrimido[5,4-d]pyrimidine (**3d**). White solid. Yield, 49%. ^1^H NMR (400 MHz, CDCl_3_) δ 4.42 (s, 8H), 2.30–2.10 (m, 8H).

2,6-dichloro-4,8-di(pyrrolidin-1-yl)pyrimido[5,4-d]pyrimidine (**3e**). White solid. Yield, 55%. ^1^H NMR (400 MHz, CDCl_3_) δ 4.31 (t, *J* = 6.7 Hz, 4H), 3.74 (t, *J* = 6.8 Hz, 4H), 2.07–1.99 (m, 4H), 1.97–1.89 (m, 4H).

2,6-dichloro-4,8-bis(2-methylpyrrolidin-1-yl)pyrimido[5,4-d]pyrimidine (**3f**). White solid. Yield, 50%. ^1^H NMR (400 MHz, CDCl_3_) δ 5.55 (s, 1H), 4.61 (s, 1H), 4.43 (s, 1H), 4.20 (s, 1H), 3.77 (d, *J* = 58.8 Hz, 2H), 2.30–1.90 (m, 6H), 1.84 (s, 1H), 1.67 (s, 1H), 1.30 (d, *J* = 6.2 Hz, 6H).

3,3′-(2,6-dichloropyrimido[5,4-d]pyrimidine-4,8-diyl)dithiazolidine (**3g**). White solid. Yield, 50%. ^1^H NMR (400 MHz, CDCl_3_) δ 5.42 (s, 2H), 4.91 (s, 2H), 4.67 (s, 2H), 4.12 (s, 2H), 3.12 (s, 4H).

2,6-dichloro-4,8-bis((R)-3-fluoropyrrolidin-1-yl)pyrimido[5,4-d]pyrimidine (**(R)-3h**). White solid. Yield, 50%.^1^H NMR (400 MHz, CDCl_3_) δ 5.56–5.17 (m, 2H), 4.99–4.59 (m, 2H), 4.55–3.59 (m, 6H), 2.68–1.84 (m, 4H).

2,6-dichloro-4,8-bis((S)-3-fluoropyrrolidin-1-yl)pyrimido[5,4-d]pyrimidine (**(S)-3h**). White solid. Yield, 42%. ^1^H NMR (400 MHz, CDCl_3_) δ 5.52–5.18 (m, 2H), 4.90–4.62 (m, 2H), 4.50–3.72 (m, 6H), 2.62–1.81 (m, 4H).

2,6-dichloro-4,8-bis(3,3-difluoropyrrolidin-1-yl)pyrimido[5,4-d]pyrimidine (**3i**). White solid. Yield, 45%. ^1^H NMR (400 MHz, CDCl_3_) δ 4.80–4.60 (m, 4H), 4.12 (t, *J* = 12.5 Hz, 2H), 4.03 (t, *J* = 7.1 Hz, 2H), 2.63–2.35 (m, 4H).

#### 3.1.3. General Procedure for Synthesis of Target Compounds **4a–i**

Different intermediate compounds **3a–i** were added to diethanolamine, stirred at 150 °C overnight. Then water was added to the mixture and yellow solids were given. The crude product was dissolved and purified on a silica gel column to yield **4a–i** as yellow solids.

2,2′,2″,2‴-((4,8-dimorpholinopyrimido[5,4-d]pyrimidine-2,6-diyl)bis(azanetriyl))tetrakis(ethan-1-ol) (**4a**). Yellow solid. Yield, 60%. ^1^H NMR (400 MHz, DMSO-*d*_6_) *δ* 4.67 (t, *J* = 5.1 Hz, 4H), 4.11 (s, 8H), 3.70 (t, 8H), 3.56 (s, 16H). ^13^C NMR (126 MHz, CDCl_3_) *δ* 160.24, 155.39, 132.21, 66.79, 62.68, 52.41, 48.11, 29.70. HRMS (ESI) calculated for C_22_H_36_N_8_O_6_ [M + H]^+^: 509.2831, found: 509.2831.

2,2′,2″,2‴-((4,8-dithiomorpholinopyrimido[5,4-d]pyrimidine-2,6-diyl)bis(azanetriyl))tetrakis(ethan-1-ol) (**4b**). Yellow solid. Yield, 64%. ^1^H NMR (500 MHz, DMSO-*d*_6_) *δ* 4.72–4.68 (m, 4H), 4.39 (s, 8H), 3.57 (s, 16H), 2.73–2.69 (m, 8H). ^13^C NMR (126 MHz, CDCl_3_) *δ* 160.13, 155.07, 132.21, 62.53, 52.55, 50.29, 27.38. HRMS (ESI) calculated for C_22_H_36_N_8_O_4_S_2_ [M + H]^+^: 541.2374, found: 541.2374.

2,2′,2″,2‴-((4,8-bis(4-fluoropiperidin-1-yl)pyrimido[5,4-d]pyrimidine-2,6-diyl)bis(azanetriyl))tetrakis(ethan-1-ol) (**4c**). Yellow solid. Yield, 41%. ^1^H NMR (400 MHz, DMSO-*d*_6_) *δ* 4.98 (s, 1H), 4.86 (s, 1H), 4.70 (s, 4H), 4.30–4.22 (m, 4H), 4.11–3.99 (m, 4H), 3.58 (s, 16H), 2.06–1.91 (m, 4H), 1.83–1.70 (m, 4H). ^13^C NMR (126 MHz, CDCl_3_) *δ* 160.36, 155.29, 132.14, 88.80, 87.44, 62.65, 52.48, 43.69 (d, *J* = 5.0 Hz), 31.38 (d, *J* = 19.8 Hz). HRMS (ESI) calculated for C_24_H_38_N_8_O_4_F_2_ [M + H]^+^: 541.3057, found: 541.3056.

2,2′,2″,2‴-((4,8-bis(4,4-difluoropiperidin-1-yl)pyrimido[5,4-d]pyrimidine-2,6-diyl)bis(azanetriyl))tetrakis(ethan-1-ol) (**4d**). Yellow solid. Yield, 90%. ^1^H NMR (500 MHz, DMSO-*d*_6_) *δ* 4.73 (t, *J* = 5.0 Hz, 4H), 4.22 (s, 8H), 3.58 (s, 16H), 2.12–2.03 (m, 8H). ^13^C NMR (126 MHz, CDCl_3_) *δ* 160.14, 155.18, 132.23, 121.76, 62.56, 52.60, 44.49, 34.11 (t, *J* = 22.9 Hz). HRMS (ESI) calculated for C_24_H_36_N_8_O_4_F_4_ [M + H]^+^: 577.2868, found: 577.2868.

2,2′,2″,2‴-((4,8-di(pyrrolidin-1-yl)pyrimido[5,4-d]pyrimidine-2,6-diyl)bis(azanetriyl))tetrakis(ethan-1-ol) (**4e**). Yellow solid. Yield, 43%. ^1^H NMR (500 MHz, DMSO-*d*_6_) *δ* 4.67 (s, 4H), 4.52–3.68 (m, 8H), 3.65–3.48 (m, 16H), 1.87 (s, 8H). HRMS (ESI) calculated for C_22_H_36_N_8_O_4_ [M + H]^+^: 477.2932, found: 477.2931.

2,2′,2″,2‴-((4,8-bis(2-methylpyrrolidin-1-yl)pyrimido[5,4-d]pyrimidine-2,6-diyl)bis(azanetriyl))tetrakis(ethan-1-ol) (**4f**). Yellow solid. Yield, 45%. ^1^H NMR (400 MHz, DMSO-*d*_6_) *δ* 4.66 (s, 4H), 3.68 (s, 4H), 3.58 (d, *J* = 4.3 Hz, 16H), 2.17–1.43 (m, 10H), 1.19 (t, *J* = 7.0 Hz, 6H). HRMS (ESI) calculated for C_24_H_40_N_8_O_4_ [M + H]^+^: 505.3245, found: 505.3248.

2,2′,2″,2‴-((4,8-bis((S)-2-methylpyrrolidin-1-yl)pyrimido[5,4-d]pyrimidine-2,6-diyl)bis(azanetriyl))tetrakis(ethan-1-ol) (**(*S*)-4f**). Yellow solid. Yield, 47%. ${\left[\alpha \right]}_{D}^{20}$ = 208.0 (c 1.0, Acetone). HRMS (ESI) calculated for C_24_H_40_N_8_O_4_ [M + H]^+^: 505.3245, found: 505.3245.

2,2′,2″,2‴-((4,8-bis((S)-2-methylpyrrolidin-1-yl)pyrimido[5,4-d]pyrimidine-2,6-diyl)bis(azanetriyl))tetrakis(ethan-1-ol) (**(*R*)-4f**). Yellow solid. Yield, 46%. ${\left[\alpha \right]}_{D}^{20}$ = −238.0 (c 1.0, Acetone). HRMS (ESI) calculated for C_24_H_40_N_8_O_4_ [M + H]^+^: 505.3245, found: 505.3245.

2,2′,2″,2‴-((4,8-di(thiazolidin-3-yl)pyrimido[5,4-d]pyrimidine-2,6-diyl)bis(azanetriyl))tetrakis(ethan-1-ol) (**4g**). Yellow solid. Yield, 60%. ^1^H NMR (400 MHz, DMSO-*d*_6_) δ 5.14 (s, 4H), 4.67 (s, 4H), 4.20 (s, 4H), 3.59 (s, 16H), 3.08 (t, *J* = 6.2 Hz, 4H). HRMS (ESI) calculated for C_20_H_32_N_8_O_4_S_2_ [M + H]^+^: 513.2061, found: 513.2061.

2,2′,2″,2‴-((4,8-bis((*S*)-3-fluoropyrrolidin-1-yl)pyrimido[5,4-d]pyrimidine-2,6-diyl)bis(azanetriyl))tetrakis(ethan-1-ol) (**(*S*)-4h**). Yellow solid. Yield, 60%. ${\left[\alpha \right]}_{D}^{20}$ = 77.0 (c 1.0, DMSO). ^1^H NMR (400 MHz, DMSO-*d*_6_) *δ* 5.47 (s, 1H), 5.34 (s, 1H), 4.79–4.55 (m, 4H), 4.27–3.78 (m, 4H), 3.60 (s, 16H), 2.28–2.13 (m, 4H). HRMS (ESI) calculated for C_22_H_34_N_8_O_4_F_2_ [M + H]^+^: 513.2744, found: 513.2742.

2,2′,2″,2‴-((4,8-bis((*R*)-3-fluoropyrrolidin-1-yl)pyrimido[5,4-d]pyrimidine-2,6-diyl)bis(azanetriyl))tetrakis(ethan-1-ol) (**(*R*)-4h**). Yellow solid. Yield, 62%. ${\left[\alpha \right]}_{D}^{20}$ = −50.0 (c 1.0, DMSO). ^1^H NMR (400 MHz, DMSO-*d*_6_) *δ* 5.47 (s, 1H), 5.34 (s, 1H), 4.68 (s, 4H), 4.30–3.79 (m, 4H), 3.74–3.43 (m, 16H), 2.30–2.15 (m, 4H). HRMS (ESI) calculated for C_22_H_34_N_8_O_4_F_2_ [M + H]^+^: 513.2744, found: 513.2742.

2,2′,2″,2‴-((4,8-bis(3,3-difluoropyrrolidin-1-yl)pyrimido[5,4-d]pyrimidine-2,6-diyl)bis(azanetriyl))tetrakis(ethan-1-ol) (**4i**) Yellow solid. Yield, 77%. ^1^H NMR (400 MHz, DMSO-*d*_6_) *δ* 4.71 (s, 4H), 4.36–3.54 (m, 28H). HRMS (ESI) calculated for C_22_H_32_N_8_O_4_F_4_ [M + H]^+^: 549.2555, found: 549.2551.

#### 3.1.4. General Procedure for Synthesis of Target Compounds **5a–c**, **6a–c**

Corresponding intermediate **3** was dissolved in N-Methyl-2-pyrrolidone, added another amine to the mixture and stirred at 100 °C overnight. Then water was added in the mixture to give yellow solids and filtered. The yellow crude product was dissolved and purified on a silica gel column to yield **5a–c**, **6a–c**.

1,1′-(4,8-di(thiazolidin-3-yl)pyrimido[5,4-d]pyrimidine-2,6-diyl)bis(piperidin-4-ol) (**5a**). Yellow solid. Yield, 43%. ^1^H NMR (400 MHz, DMSO-*d*_6_) δ 5.16 (s, 4H), 4.66 (d, *J* = 4.3 Hz, 2H), 4.33–4.06 (m, 8H), 3.72–3.63 (m, 2H), 3.21–2.94 (m, 8H), 1.82–1.70 (m, 4H), 1.40–1.27 (m, 4H). ^13^C NMR (126 MHz, CDCl_3_) δ 158.23, 154.97, 132.27, 68.70, 52.27, 42.48, 34.23. HRMS (ESI) calculated for C_22_H_32_N_8_O_2_S_2_ [M + H]^+^: 505.2162, found: 505.2159.

((4,8-di(thiazolidin-3-yl)pyrimido[5,4-d]pyrimidine-2,6-diyl)bis(piperidine-1,3-diyl))dimethanol (**5b**). Yellow solid. Yield, 55%. ^1^H NMR (400 MHz, DMSO-*d*_6_) δ 5.17 (s, 4H), 4.56–4.44 (m, 4H), 4.35 (d, *J* = 12.5 Hz, 2H), 4.21 (s, 4H), 3.27–3.24 (m, 2H), 3.08 (t, *J* = 6.1 Hz, 4H), 2.92–2.80 (m, 3H), 2.67–2.54 (m, 3H), 1.75–1.51 (m, 6H), 1.44–1.35 (m, 2H), 1.22–1.13 (m, 2H). HRMS (ESI) calculated for C_24_H_36_N_8_O_2_S_2_ [M + H]^+^: 533.2475, found: 533.2474.

((3*S*,3′*S*)-(4,8-di(thiazolidin-3-yl)pyrimido[5,4-d]pyrimidine-2,6-diyl)bis(piperidine-1,3-diyl))dimethanol (**(*S*)-5b**). Yellow solid. Yield, 50%. ${\left[\alpha \right]}_{D}^{20}$ = 27.0 (c 1.0, Acetone). HRMS (ESI) calculated for C_24_H_36_N_8_O_2_S_2_ [M + H]^+^: 533.2475, found: 533.2475.

((3*R*,3′*R*)-(4,8-di(thiazolidin-3-yl)pyrimido[5,4-d]pyrimidine-2,6-diyl)bis(piperidine-1,3-diyl))dimethanol (**(*R*)-5b**). Yellow solid. Yield, 52%. ${\left[\alpha \right]}_{D}^{20}$ = 32.0 (c 1.0, DMSO). HRMS (ESI) calculated for C_24_H_36_N_8_O_2_S_2_ [M + H]^+^: 533.2475, found: 533.2476.

(3S,3′S)-1,1′-(4,8-di(thiazolidin-3-yl)pyrimido[5,4-d]pyrimidine-2,6-diyl)bis(pyrrolidin-3-ol) (**(*S*)-5c**). Yellow solid. Yield, 50%. ${\left[\alpha \right]}_{D}^{20}$ = 60.0 (c 1.0, DMSO) ^1^H NMR (400 MHz, DMSO-*d*_6_) *δ* 5.26 (s, 4H), 4.89 (d, *J* = 3.1 Hz, 2H), 4.38–4.18 (m, 6H), 3.58–3.50 (m, 6H), 3.41 (s, 2H), 3.08 (t, *J* = 6.2 Hz, 4H), 2.08–1.91 (m, 2H), 1.91–1.71 (m, 2H). ^13^C NMR (126 MHz, DMSO) *δ* 158.00, 153.48, 131.94, 69.75, 55.18, 52.11, 51.83, 44.78, 34.10. HRMS (ESI) calculated for C_20_H_28_N_8_O_2_S_2_ [M + H]^+^: 477.1849, found: 477.1842.

(3R,3′R)-1,1′-(4,8-di(thiazolidin-3-yl)pyrimido[5,4-d]pyrimidine-2,6-diyl)bis(pyrrolidin-3-ol) (**(*R*)-5c**). Yellow solid. Yield, 60%. ${\left[\alpha \right]}_{D}^{20}$ = −60.0 (c 1.0, DMSO). ^1^H NMR (400 MHz, DMSO-*d*_6_) *δ* 5.26 (s, 4H), 4.88 (d, *J* = 3.6 Hz, 2H), 4.39–4.13 (m, 6H), 3.60–3.47 (m, 6H), 3.41 (s, 2H), 3.08 (t, *J* = 6.3 Hz, 4H), 2.02–1.94 (m, 2H), 1.88–1.80 (m, 2H). ^13^C NMR (126 MHz, DMSO) *δ* 157.98, 153.46, 131.93, 69.75, 55.17, 52.11, 51.82, 44.77, 34.10. HRMS (ESI) calculated for C_20_H_28_N_8_O_2_S_2_ [M + H]^+^: 477.1849, found: 477.1849.

1,1′-(4,8-bis(4-fluoropiperidin-1-yl)pyrimido[5,4-d]pyrimidine-2,6-diyl)bis(piperidin-3-ol) (**6a**). Yellow solid. Yield, 58%. ^1^H NMR (500 MHz, DMSO-*d*_6_) *δ* 5.09–4.66 (m, 4H), 4.32 (s, 2H), 4.28–4.01 (m, 8H), 3.44 (s, 2H), 2.85 (s, 2H), 2.70 (s, 2H), 2.07–1.94 (m, 4H), 1.93–1.84 (m, 2H), 1.84–1.72 (m, 4H), 1.73–1.63 (m, 2H), 1.47–1.28 (m, 4H).^13^C NMR (126 MHz, CDCl_3_) *δ* 160.32, 155.09, 132.70, 89.43, 88.07, 66.79, 51.32, 45.00, 43.73, 32.97, 31.59 (d, *J* = 19.2 Hz), 21.98. HRMS (ESI) calculated for C_26_H_38_N_8_O_2_F_2_ [M + H]^+^: 533.3159, found: 533.3157.

(3R,3′R)-1,1′-(4,8-bis(4-fluoropiperidin-1-yl)pyrimido[5,4-d]pyrimidine-2,6-diyl)bis(piperidin-3-ol) (**(*R*)-6a**). Yellow solid. Yield, 49%. ${\left[\alpha \right]}_{D}^{20}$ = 40.0 (c 1.0, DMSO). HRMS (ESI) calculated for C_26_H_38_N_8_O_2_F_2_ [M + H]^+^: 533.3159, found: 533.3159.

((4,8-bis(4-fluoropiperidin-1-yl)pyrimido[5,4-d]pyrimidine-2,6-diyl)bis(piperidine-1,3-diyl))dimethanol (**6b**). Yellow solid. Yield, 57%. ^1^H NMR (500 MHz, DMSO) *δ* 4.97 (s, 1H), 4.87 (s, 1H), 4.48 (s, 4H), 4.34 (d, *J* = 10.5 Hz, 2H), 4.24 (s, 4H), 4.07 (s, 4H), 3.28–3.23 (m, 2H), 2.89–2.77 (m, 2H), 2.62–2.53 (m, 2H), 2.05–1.95 (m, 4H), 1.84–1.69 (m, 6H), 1.65 (d, *J* = 11.1 Hz, 2H), 1.56 (s, 2H), 1.45–1.35 (m, 2H), 1.22–1.11 (m, 2H). HRMS (ESI) calculated for C_28_H_42_N_8_O_2_F_2_ [M + H]^+^: 561.3472, found: 561.3474.

1,1′-(4,8-bis(4-fluoropiperidin-1-yl)pyrimido[5,4-d]pyrimidine-2,6-diyl)bis(pyrrolidin-3-ol) (**6c**). Yellow solid. Yield, 46%. ${\left[\alpha \right]}_{D}^{20}$ = 55.0 (c 1.0, Acetone). ^1^H NMR (400 MHz, DMSO-*d*_6_) *δ* 4.86 (s, 2H), 4.38–4.30 (m, 6H), 4.13 (s, 4H), 3.55–3.48 (m, 6H), 2.06–1.91 (m, 8H), 1.89–1.71 (m, 8H). HRMS (ESI) calculated for C_24_H_34_N_8_O_2_F_2_ [M + H]^+^: 505.2846, found: 505.2846.

#### 3.1.5. General Procedure for Synthesis of Intermediate Compounds **7**, **8a** and **8b**

To compound **2** (0.10 g, 0.37 mmol) and potassium carbonate (0.10 g, 0.74 mmol) in THF (6 mL), stirred under nitrogen at −78 °C, thiazolidine (0.04 g, 0.42 mmol) in THF (6 mL) was added dropwise from a syringe at a rate of 1 mL per min. After warming up for 10 min, a cloudy yellow solution was obtained. Water was added in the mixture, crude products were gained, filtered, dissolved and purified by silica gel column to yield **7**. To a stirred solution of **7** in dry THF at 0 °C a solution of the appropriate amine (2.0 mol equiv.) in THF was added, stirring for 10–30 min. Water was added and extracted with ethyl acetate. The crude product was dried and purified by silica column chromatography.

3-(2,6,8-trichloropyrimido[5,4-d]pyrimidin-4-yl)thiazolidine (**7**). A pale-yellow solid. Yield, 60%. ^1^H NMR (400 MHz, CDCl_3_) δ 5.43 (s, 1H), 4.97 (s, 1H), 4.66 (t, *J* = 6.2 Hz, 1H), 4.22 (t, *J* = 6.3 Hz, 1H), 3.28 (t, *J* = 6.2 Hz, 1H), 3.14 (t, *J* = 6.3 Hz, 1H).

4-(2,6-dichloro-8-(thiazolidin-3-yl)pyrimido[5,4-d]pyrimidin-4-yl)thiomorpholine (**8a**). White solid. Yield, 55%. ^1^H NMR (400 MHz, CDCl_3_) δ 5.41 (s, 2H), 4.59 (m, 4H), 4.13 (s, 2H), 3.11 (m, 2H), 2.92–2.65 (m, 4H).

3-(2,6-dichloro-8-((3S)-3-fluorocyclopentyl)pyrimido[5,4-d]pyrimidin-4-yl)thiazolidine (**8b**). White solid. Yield, 50%. ^1^H NMR (400 MHz, CDCl_3_) δ 5.47 (s, 1H), 5.41–5.24 (m, 2H), 4.89–4.70 (m, 2H), 4.36–3.97 (m, 4H), 3.13 (s, 2H), 2.58–2.09 (m, 2H).

#### 3.1.6. General Procedure for Synthesis of Target Compounds **9a** and **9b**

The synthesis method was the same as that used for **4a–i**.

2,2′,2″,2‴-((4-(thiazolidin-3-yl)-8-thiomorpholinopyrimido[5,4-d]pyrimidine-2,6-diyl)bis(azanetriyl))tetrakis(ethan-1-ol) (**9a**). Yellow solid. Yield, 45%. ^1^H NMR (500 MHz, CDCl_3_) δ 5.15 (s, 2H), 4.40–4.27 (m, 4H), 4.27–4.15 (m, 2H), 3.97–3.84 (m, 8H), 3.82–3.72 (m, 8H), 3.67 (s, 2H), 3.49 (s, 2H), 3.14–3.03 (m, 2H), 2.85–2.62 (m, 4H). ^13^C NMR (126 MHz, DMSO-*d*_6_) δ 158.73, 158.35, 157.83, 154.10, 153.94, 153.60, 132.20, 132.01, 131.77, 59.82, 52.37, 52.31, 52.10, 52.02, 51.96, 51.88, 50.0, 27.00. HRMS (ESI) calculated for C_21_H_34_N_8_O_4_S_2_ [M + H]^+^: 527.2217, found: 527.2218.

(*S*)-2,2′,2″,2‴-((4-(3-fluoropyrrolidin-1-yl)-8-(thiazolidin-3-yl)pyrimido[5,4-d]pyrimidine-2,6-diyl)bis(azanetriyl))tetrakis(ethan-1-ol) (**9b**). Yellow solid. Yield, 46%. ${\left[\alpha \right]}_{D}^{20}$ = 68.0 (c 1.0, Acetone). ^1^H NMR (400 MHz, CDCl_3_) δ 5.40 (s, 1H), 5.30–5.07 (m, 4H), 4.28–4.10 (m, 4H), 4.05–3.99 (m, 2H), 3.89 (s, 16H), 3.79–3.66 (m, 2H), 3.08 (t, *J* = 5.7 Hz, 2H), 2.43–2.24 (m, 2H). ^13^C NMR (101 MHz, DMSO-*d*6) δ 157.69, 157.50, 154.49, 154.17, 132.11, 131.07, 60.01 (d, *J* = 9.7 Hz), 52.00 (d, *J* = 20.5 Hz), 51.75, 49.06, 29.95. HRMS (ESI) calculated for C_21_H_33_N_8_O_4_FS [M + H]^+^: 513.2402, found: 513.2406.

### 3.2. In Vitro Assay for PDE5 Inhibitors

#### 3.2.1. Protein Expression and Purification

The purification and expression of PDE5A protein is the same as the experimental protocols in our previous publication [23,24]. In brief, the catalytic structural domain encoding PDE5A (535-860) was cloned into the vector pET-15b, and then the cDNA was transferred to the E. coli strain BL21 (CodonPlus, Stratagene) for overexpression. When the cell carrying the plasmid was cultivated in LB medium at 37 °C to OD600 = 0.7, 0.1 mM isopropyl *β*-Dthiogalactopyranoside was added to induce PDE5A expression for an additional 40 h of growth at 15 °C. The PDE5A protein was purified through a Ni-NTA column (∅ = 2.5 cm, 15 mL QIAGEN agarose beads), Q-column (∅ 2.5 cm × 8 cm, GE Healthcare) and Superdex 200 column (∅ 2.5 cm × 45 cm, GE Healthcare). A typical batch yielded about 15 mg of the PDE5A protein from 2 L of cell culture with a purity >95% based on SDS-PAGE.

#### 3.2.2. Enzymatic Assay of PDE5A

Using a 100-fold dilution of ^3^H-cGMP as the substrate, the assay buffer contained 50 mM Tris-HCl (pH 8.0), 10 mM MgCl_2_ and 1 mM DTT. Different solutions of tested compounds were added to the test group, DMSO was added to the negative control and blank control groups and sildenafil was added to the positive control group. Then protein solutions diluted with assay buffer were added and the blank group was added to assay buffer with no protein. The enzymatic reaction was performed at room temperature for 15 min, and then 0.2 M ZnSO_4_ was added to halt the reaction and 0.2 N Ba(OH)_2_ solution was added to precipitate the reaction product, and unreacted ^3^H-cGMP remained in the supernatant. The radioactivity of the supernatant was measured in 2.5 mL of Ultima Gold liquid scintillation cocktails using a liquid scintillation counter and the inhibitory activity of the compound against PDE5A was calculated. The above tests were performed three times.

### 3.3. Molecular Docking

The Accelrys Discovery Studio 2.5.5 software was used for molecular docking studies. Hydrogen atoms and partial charges were added to the crystal structure of PDE5 (PDB entry code 2H42) with the sildenafil bound using the CHARMM force field and the Momany–Rone partial charge method. All ionizable residues’ protonation states in the systems were determined at the pH value of 7.4. The zinc and magnesium ions were assigned with a charge of +2. Original ligand in the complex was used to define the docking site of PDE5, and the radius of the input sphere was set to 10 Å from the center of the site. To determine the optimal parameters for a reliable docking method, the original inhibitor was extracted from the crystal structures and redocked back into the crystal structure. The CDOCKER method embedded in Accelrys Discovery Studio (version 2.5.5, Accelrys Inc., San Diego, CA, USA) was suitable for PDE5. Average RSMD (root-mean-square deviation) values were less than 1.0 Å for the docking poses compared to the original X-ray pose. Then, the default parameters were used for the docking of the designed molecules. Twenty conformations were prepared for each molecule.

## 4. Conclusions

In conclusion, in this study, a series of novel pyrimidopyrimidine derivatives have been designed, synthesized and evaluated as PDE5 inhibitors with the assistance of molecular docking. Most of the designed compounds showed good inhibition ratios against PDE5. Compounds with comparable activity to dipyridamole exhibited excellent metabolic stability, among which compound **(*S*)-4h** was stable in rat liver microsomes with a T_1/2_ of 67 min. The SAR of target compounds was rationally explained by binding patterns, providing evidence for the further rational design of novel PDE5 inhibitors for the treatment of IPF.

## Figures and Tables

**Table 1:** The inhibitory activities of the target compounds **4a–i** against PDE5.

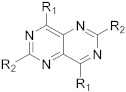					
Comp.	R_1_	R_2_	Inhibition Ratio (%)		IC_50_ (nM) ^a^
			0.5 μM	0.1 μM	
DIP	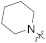	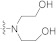	84	17	235 ± 35
**4a**	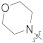	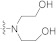	40	0	-
**4b**	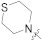	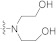	75	53	113 ± 11
**4c**	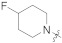	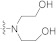	62	31	343 ± 18
**4d**	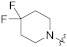	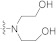	72	41	362 ± 81
**4e**	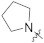	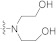	47	7	-
**4f**	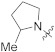	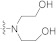	60	14	478 ± 3
**(*S*)-4f**	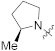	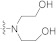	42	16	-
**(*R*)-4f**	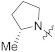	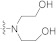	44	27	-
**4g**	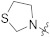	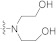	93	60	64 ± 16
**(*S*)-4h**	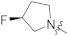	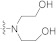	55	34	332 ± 56
**(*R*)-4h**	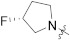	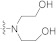	44	27	-
**4i**	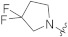	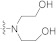	61	24	353 ± 57

^a^ The SD values are presented as the means of three independent experiments. Sildenafil was used as the reference compound with an IC_50_ of 1.3 nM.

**Table 2:** The inhibitory activities of target compounds **5a–c**, **6a–c** against PDE5.

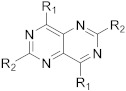					
Comp.	R_1_	R_2_	Inhibition Ratio (%)		IC_50_ (nM) ^a^
			0.5 μM	0.1 μM	
**5a**	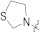	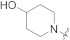	32	29	-
**5b**	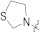	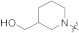	79	52	167 ± 41
**(*S*)-5b**	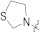	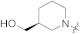	34	13	-
**(*R*)-5b**	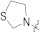	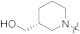	-	-	109 ± 20
**(*S*)-5c**	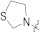	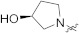	49	36	-
**(*R*)-5c**	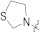	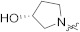	74	45	198 ± 45
**6a**	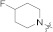	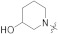	48	7	-
**(*R*)-6a**	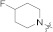	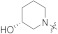	36	8	-
**6b**	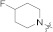	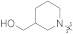	7	0	-
**6c**	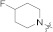	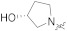	20	5	-

^a^ The SD values are presented as the means of three independent experiments. Sildenafil was used as the reference compound with an IC_50_ of 1.3 nM.

**Table 3:** The inhibitory activities of target compounds **9a** and **9b** against PDE5.

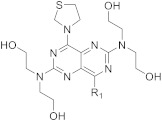				
Comp.	R_1_	Inhibition Ratio (%)		IC_50_ (nM) ^a^
		0.5 μM	0.1 μM	
**9a**	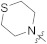	91	45	100 ± 1
**9b**	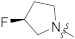	81	33	94 ± 9

^a^ The SD values are presented as the means of three independent experiments. Sildenafil was used as the reference compound with an IC_50_ of 1.3 nM.

**Table 4:** The metabolic stability of typical compounds in RLM.

Comp.	PDE5 IC_50_ (nM)	T_1/2_ in RLM (min)
DIP	235	7
**4b**	113	7
**4c**	343	44
**4d**	362	19
**4g**	64	5
**(*S*)-4h**	332	67
**9a**	100	4
**9b**	94	10

## Data Availability

The data presented in this study are available within the article and Supplementary Materials.

## Supplementary Materials

The following supporting information can be downloaded at: https://www.mdpi.com/article/10.3390/molecules27113452/s1. The spectra of ^1^H-NMR, ^13^C-NMR and HRMS for the target compounds are available online.

## Sample Availability

Samples of the compounds are not available from the authors.
