# Supplementary material for: Discovery of Dipyridamole Analogues with Enhanced Metabolic Stability for the Treatment of Idiopathic Pulmonary Fibrosis
Source: Molecules. 2022 May 26;27(11):3452. doi: 10.3390/molecules27113452 (PMC9182104; doi:10.3390/molecules27113452)
Supplement: Supplementary file 1 [file molecules-27-03452-s001.zip › molecules-1685224-supplementary.pdf]

Discovery of Dipyridamole Analogues with Enhanced Metabolic Stability for the Treatment of Idiopathic Pulmonary Fibrosis.

Meng-Xing Huang [1], Yan-Quan Chen [1], Run-Duo Liu [1], Yue Huang [1] and Chen Zhang [1,2,*]

[1]*School of Pharmaceutical Sciences, Sun Yat-sen University, Guangzhou 510006, P. R. China;*

[2]*School of Chemistry and Chemical Engineering, Guangdong Pharmaceutical University, Zhongshan 528458, P. R. China*

* Corresponding author at: **zhangch328@mail.sysu.edu.cn**

Table of Contents:

1. Figure S1. Predicted binding modes of representative compounds.......................S2
2. IC_{50} curves of designed compounds...S3
3. Rat liver microsomal stability...S4
4. ^{1}H NMR and ^{13}C NMR spectrums of the target compounds................................S5
5. HRMS spectra of target compounds...S19
6. References...S42

1. Figure S1. Predicted binding modes of representative compounds.

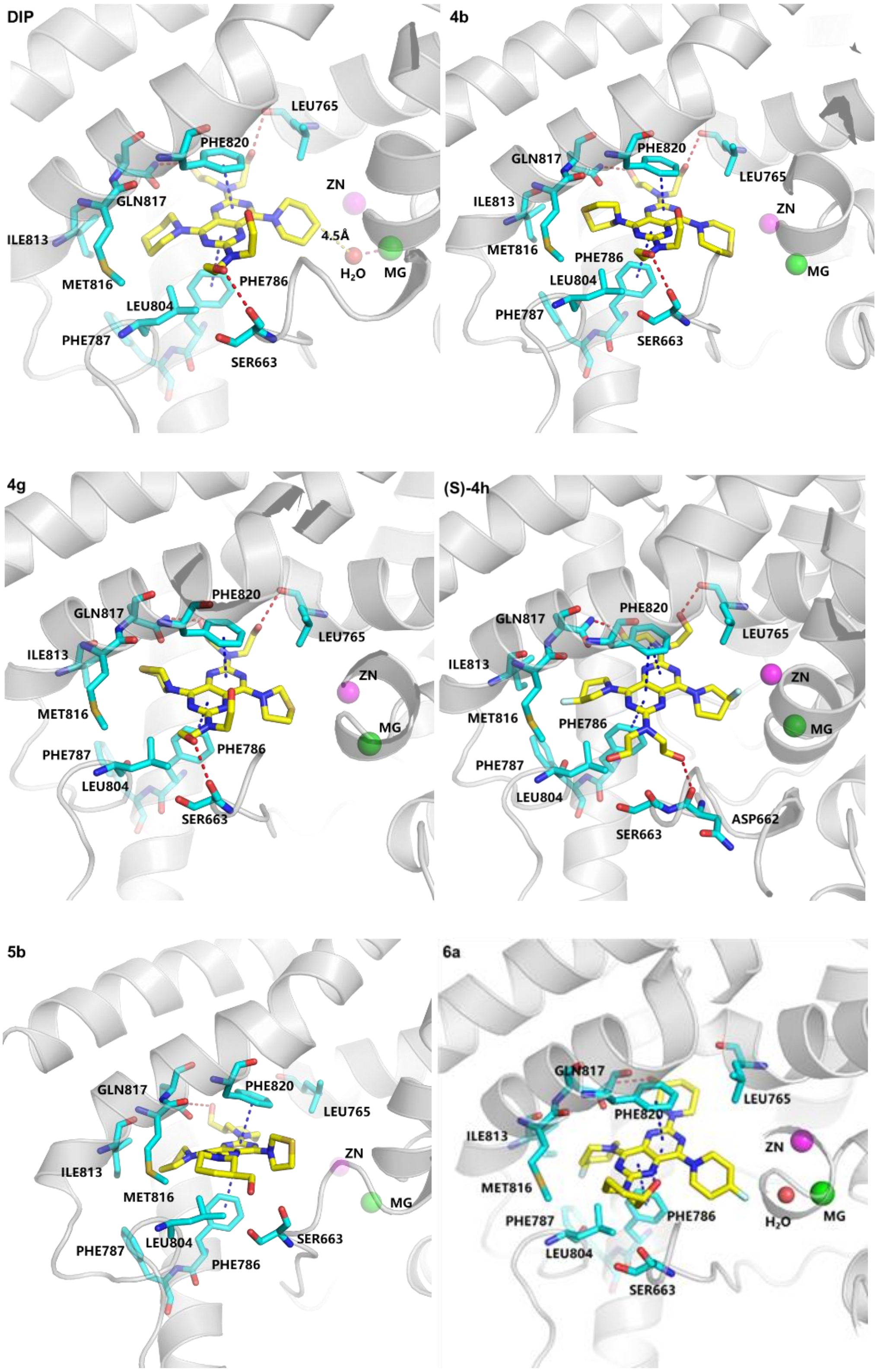

2. IC$_{50}$ curves of designed compounds.

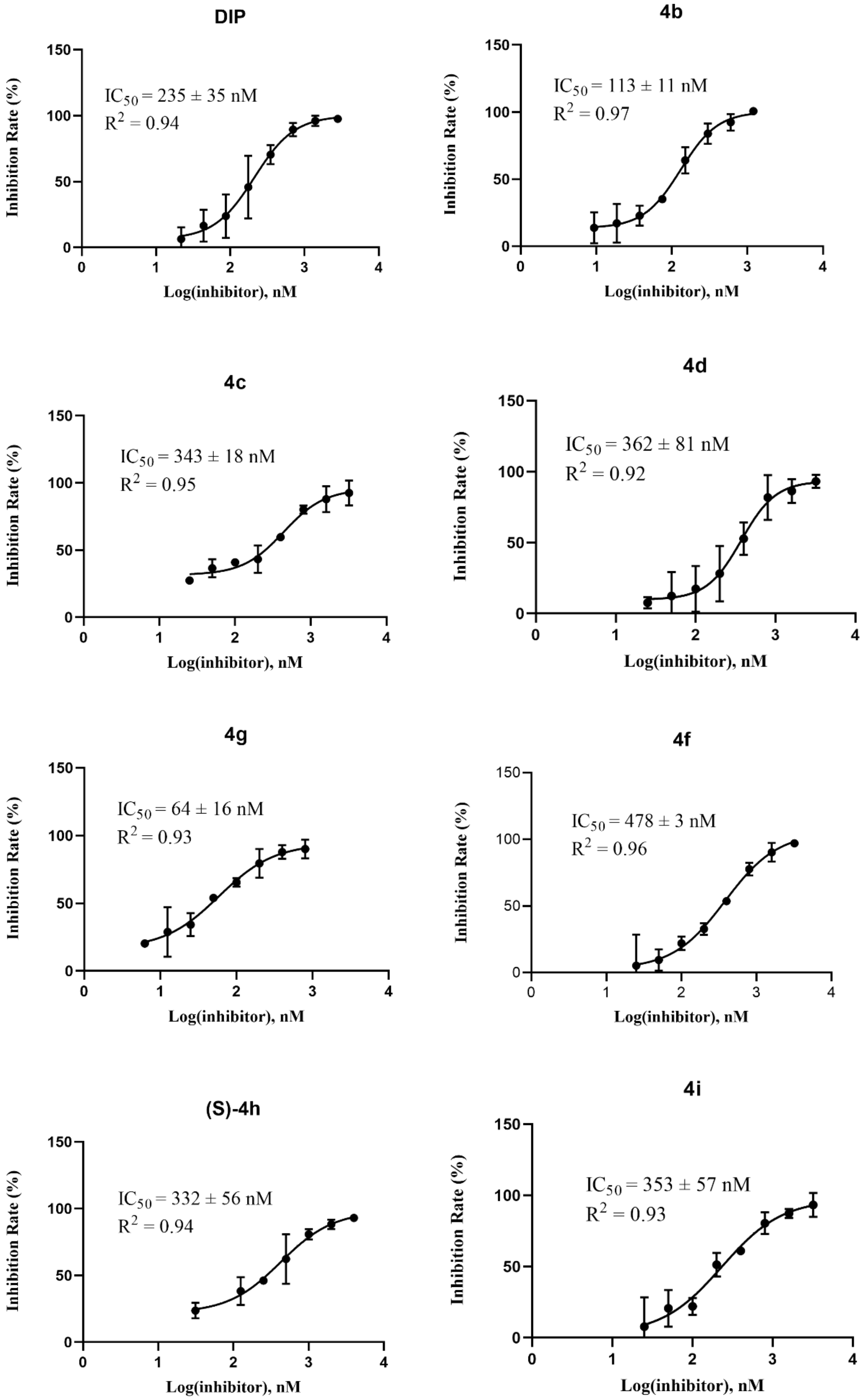

5b

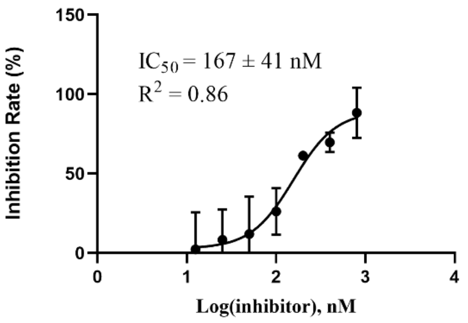

(R)-5b

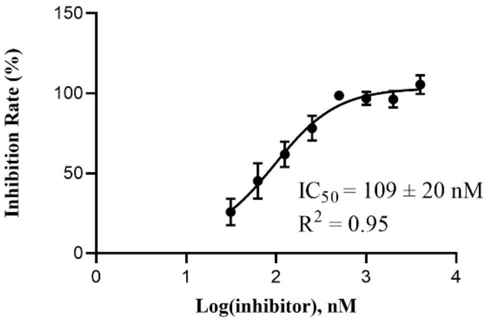

(R)-5c

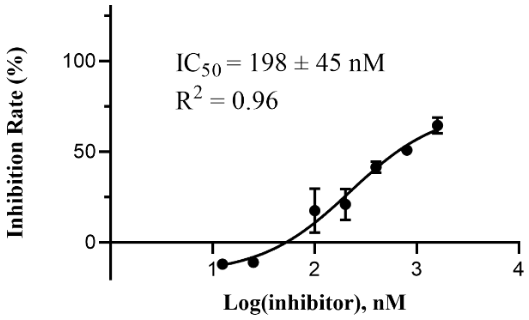

9a

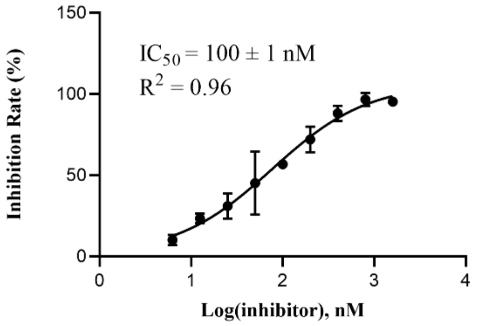

9b

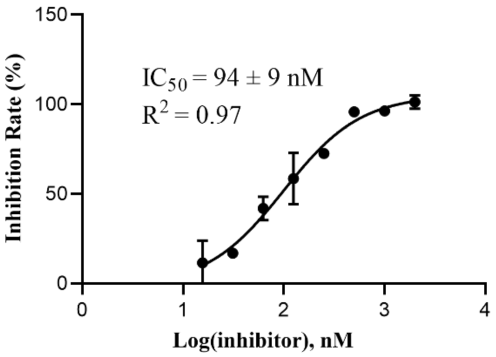

3. Rat liver microsomal stability.

The assays were performed at the Medicilon Company, Shanghai, China and PreceDo Pharmaceuticals Co.Ltd. Hefei, China. The experimental procedures were similar to those in our previous study. Each tested compound was dissolved in 100% DMSO to prepare a 10 mM stock solution and diluted to a final concentration of 0.5 μM for the experiments.

4. ^{1}H NMR and ^{13}C NMR spectrums of the target compounds.

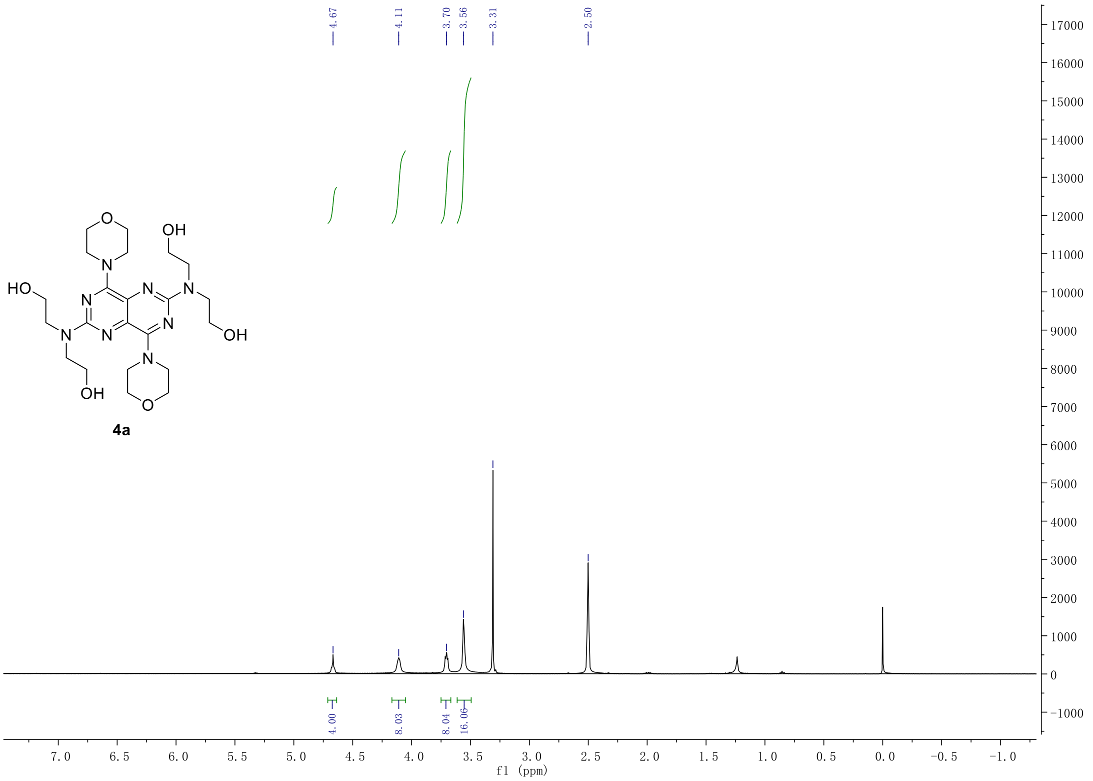

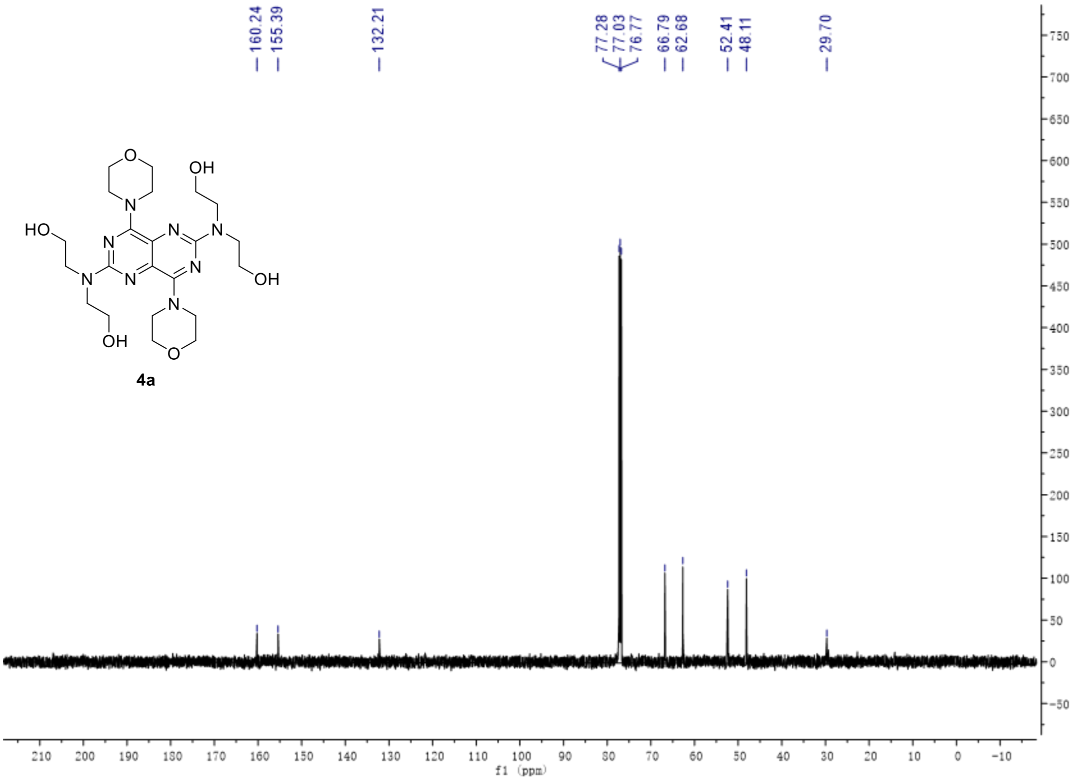

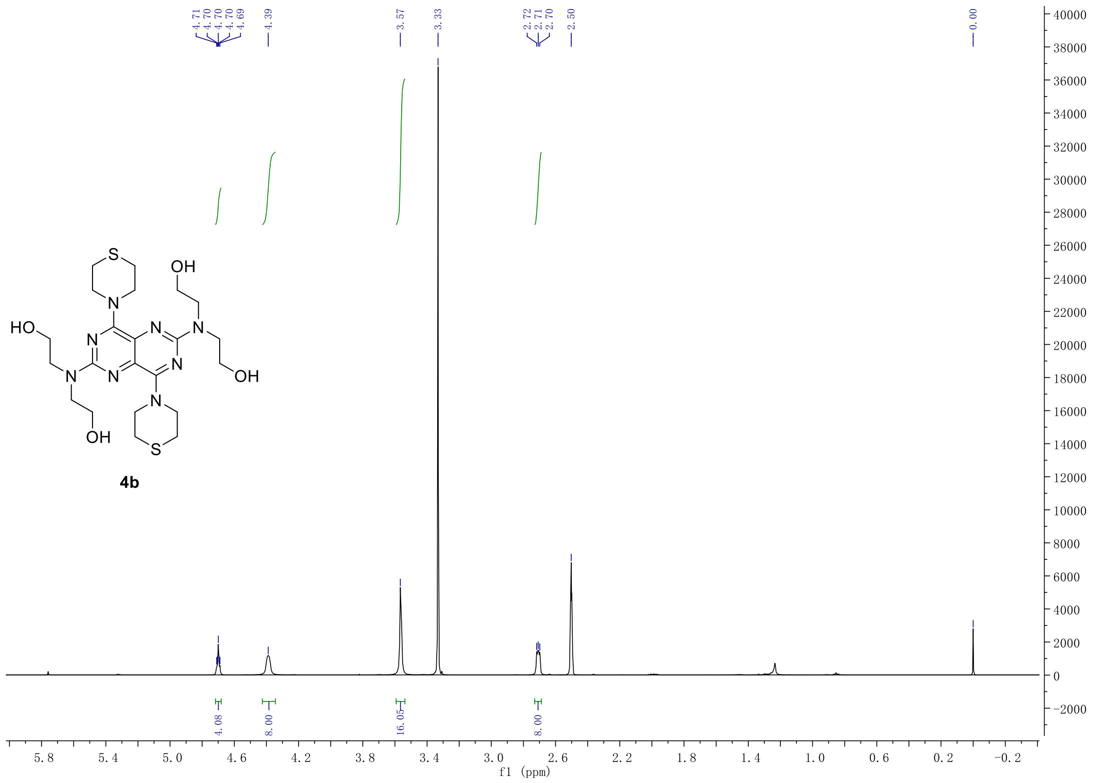
4.71
4.70
4.70
4.70
4.69
4.39
3.57
3.33
2.72
2.71
2.70
2.50
0.00
4b
4.08
8.00
16.05
8.00
f1 (ppm)

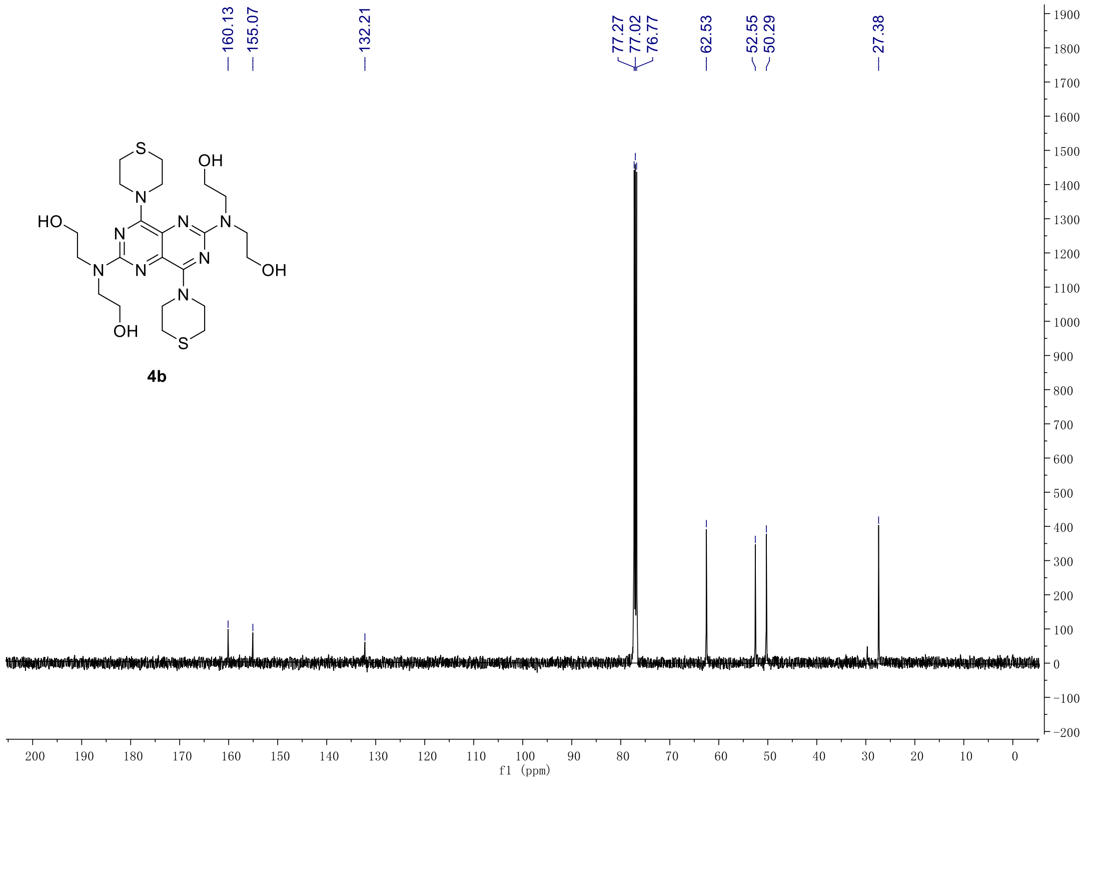
160.13
155.07
132.21
77.27
77.02
76.77
62.53
52.55
50.29
27.38
4b
f1 (ppm)

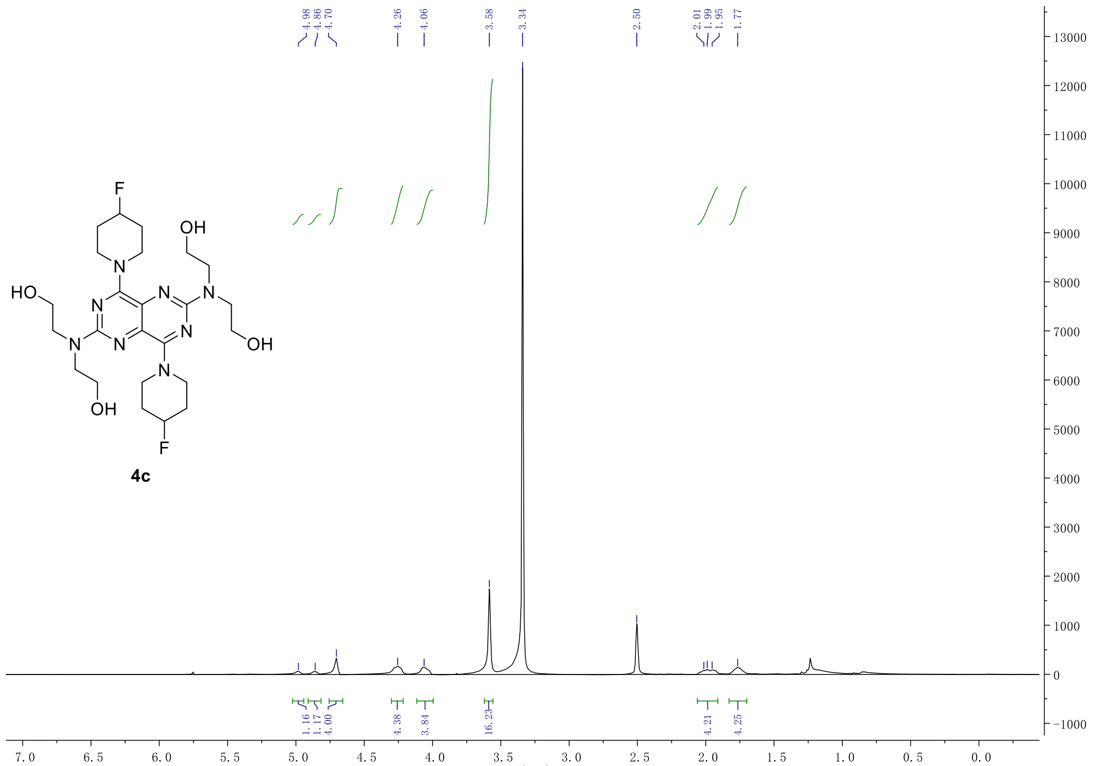
4.98
4.86
4.70
4.26
4.06
3.58
3.34
2.50
2.01
1.99
1.95
1.77
1.16
1.17
4.00
4.38
3.84
16.23
4.21
4.25
F
OH
HO
N
OH
OH
4c
f1 (ppm)

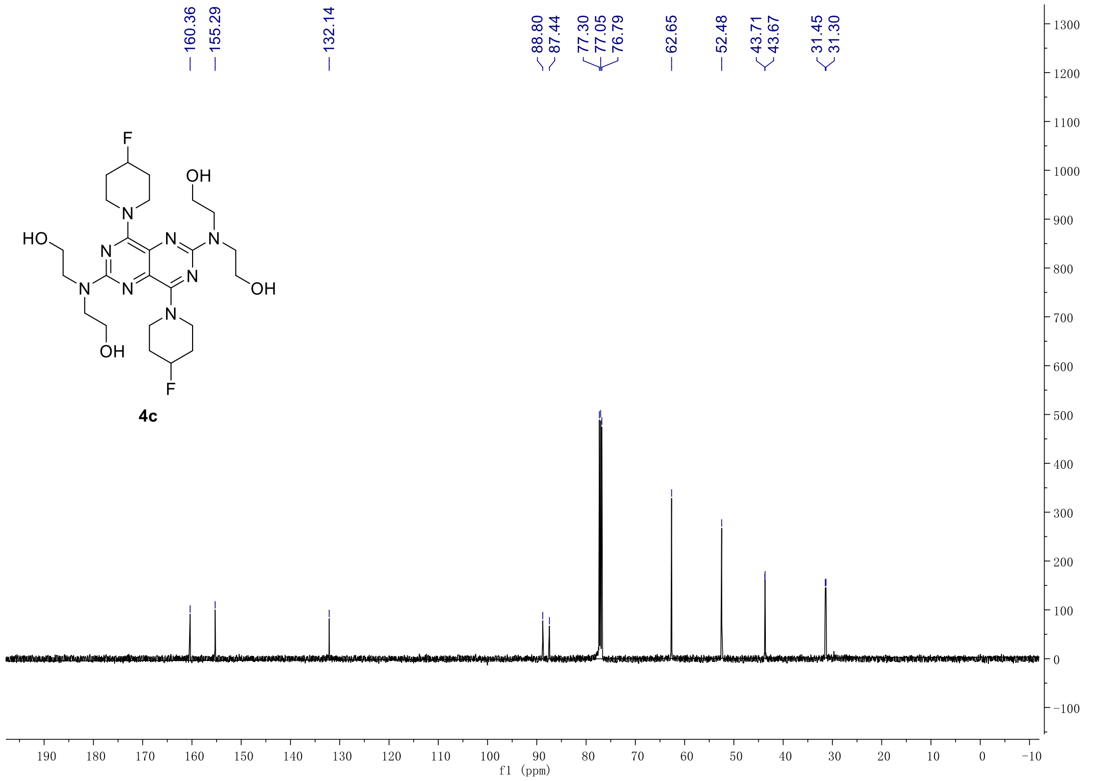
160.36
155.29
132.14
88.80
87.44
77.30
77.05
76.79
62.65
52.48
43.71
43.67
31.45
31.30
F
OH
HO
N
OH
OH
4c
f1 (ppm)

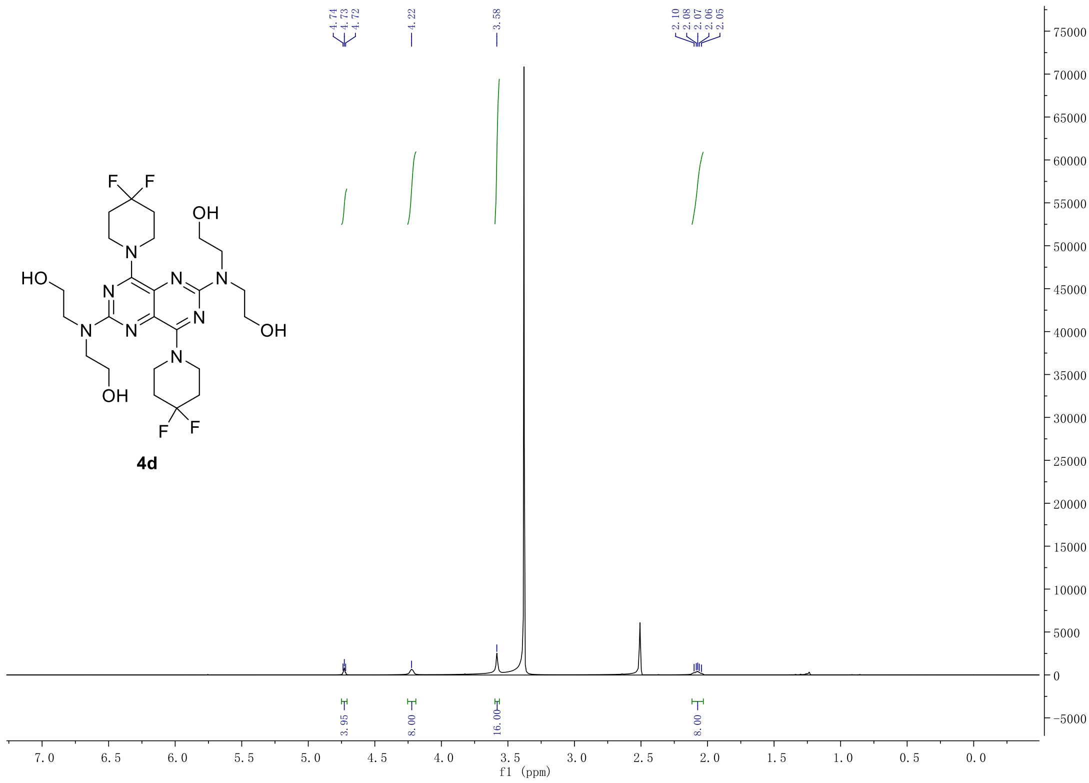
4.74
4.73
4.72
4.22
3.58
2.10
2.08
2.07
2.06
2.05
3.95
8.00
16.00
8.00
F
F
OH
HO
N
N
N
N
N
N
OH
OH
F
F
4d
f1 (ppm)

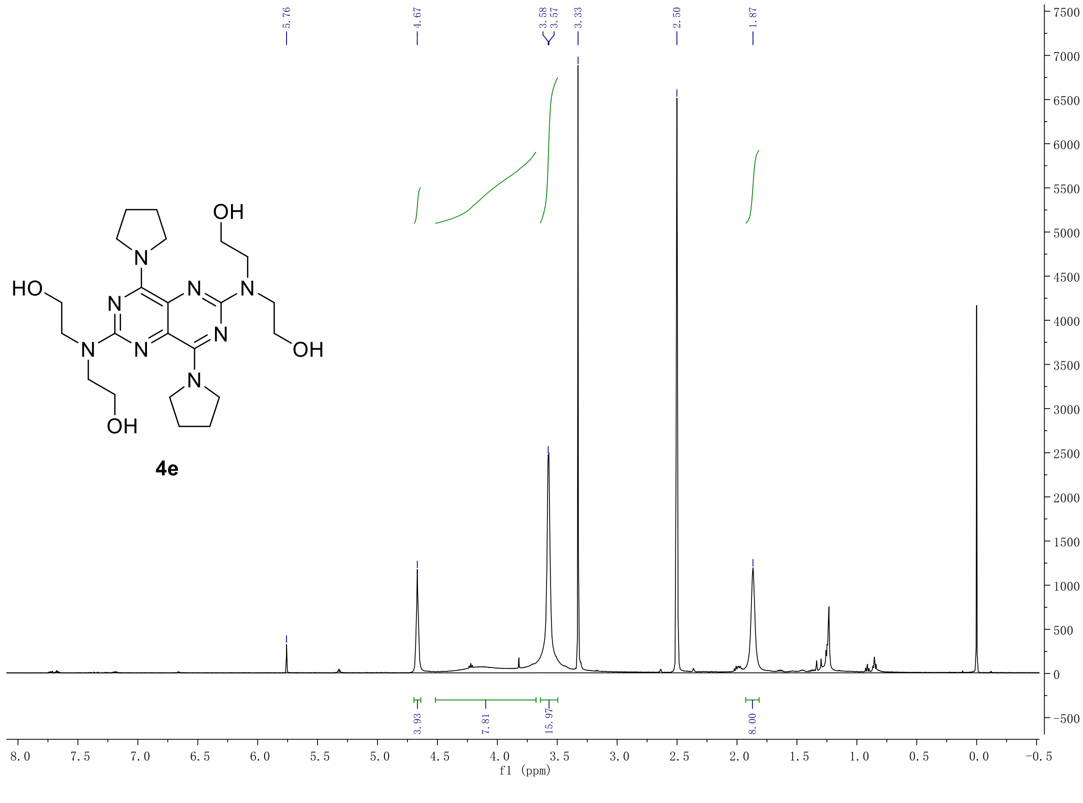
5.76
4.67
3.58
3.57
3.33
2.50
1.87
3.93
7.81
15.97
8.00
OH
HO
N
N
N
N
N
OH
OH
4e
f1 (ppm)

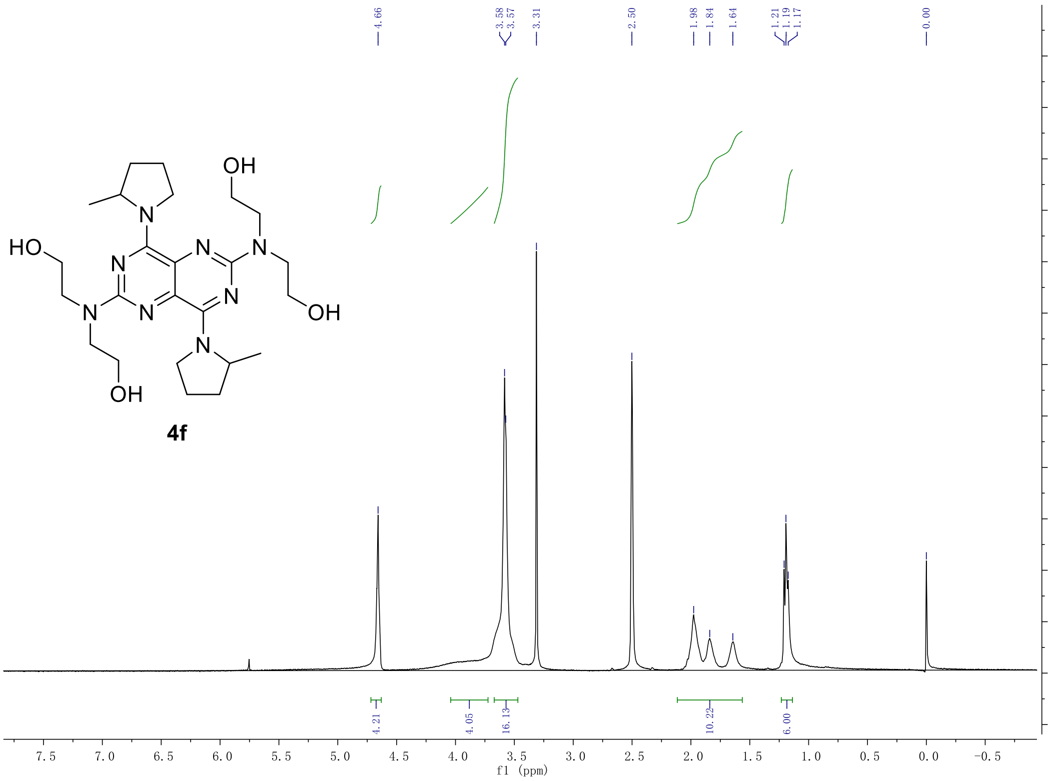
4.66
3.58
3.57
3.31
2.50
1.98
1.84
1.64
1.21
1.19
1.17
0.00
4.21
4.05
16.13
10.22
6.00
4f
f1 (ppm)

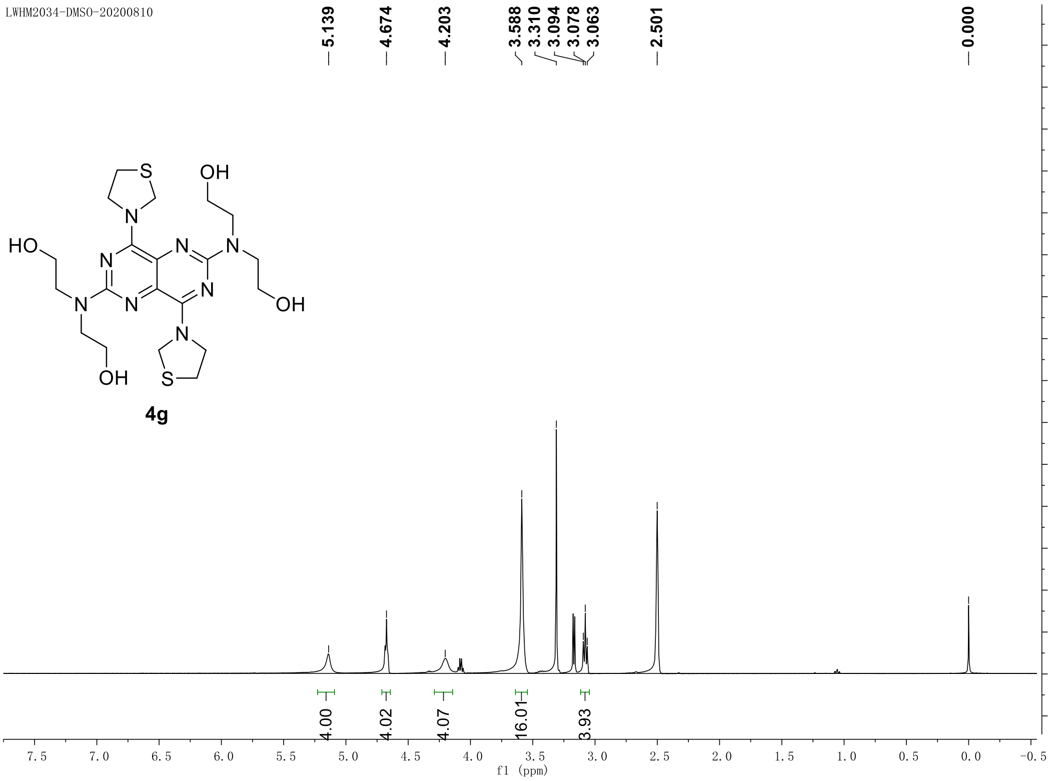
LWHM2034-DMSO-20200810
5.139
4.674
4.203
3.588
3.310
3.094
3.078
3.063
2.501
0.000
4.00
4.02
4.07
16.01
3.93
4g
f1 (ppm)

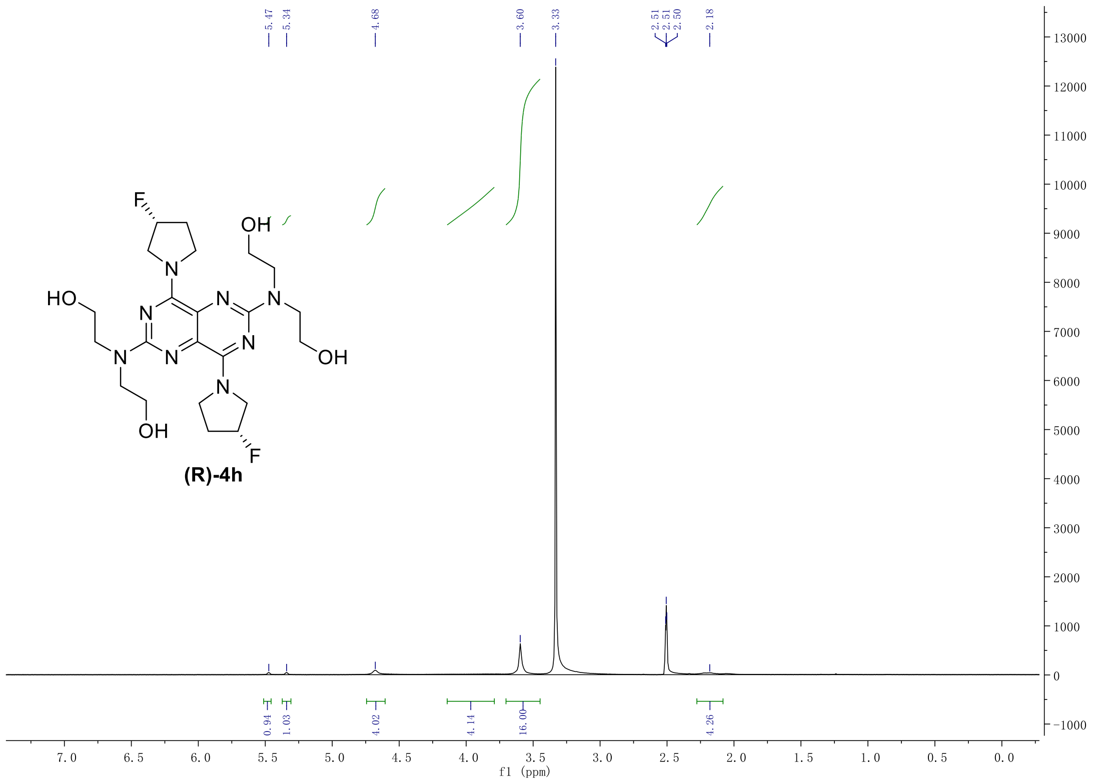
(R)-4h
5.47
5.34
4.68
3.60
3.33
2.51
2.51
2.50
2.18
0.94
1.03
4.02
4.14
16.00
4.26
f1 (ppm)

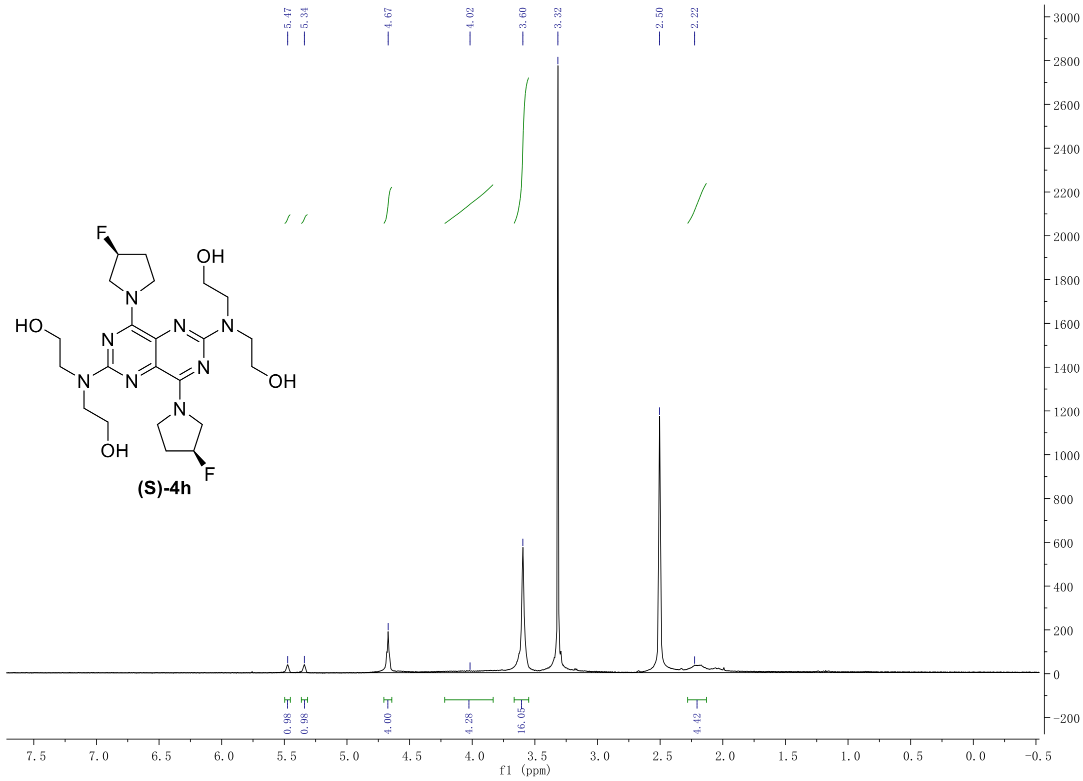
(S)-4h
5.47
5.34
4.67
4.02
3.60
3.32
2.50
2.22
0.98
0.98
4.00
4.28
16.05
4.42
f1 (ppm)

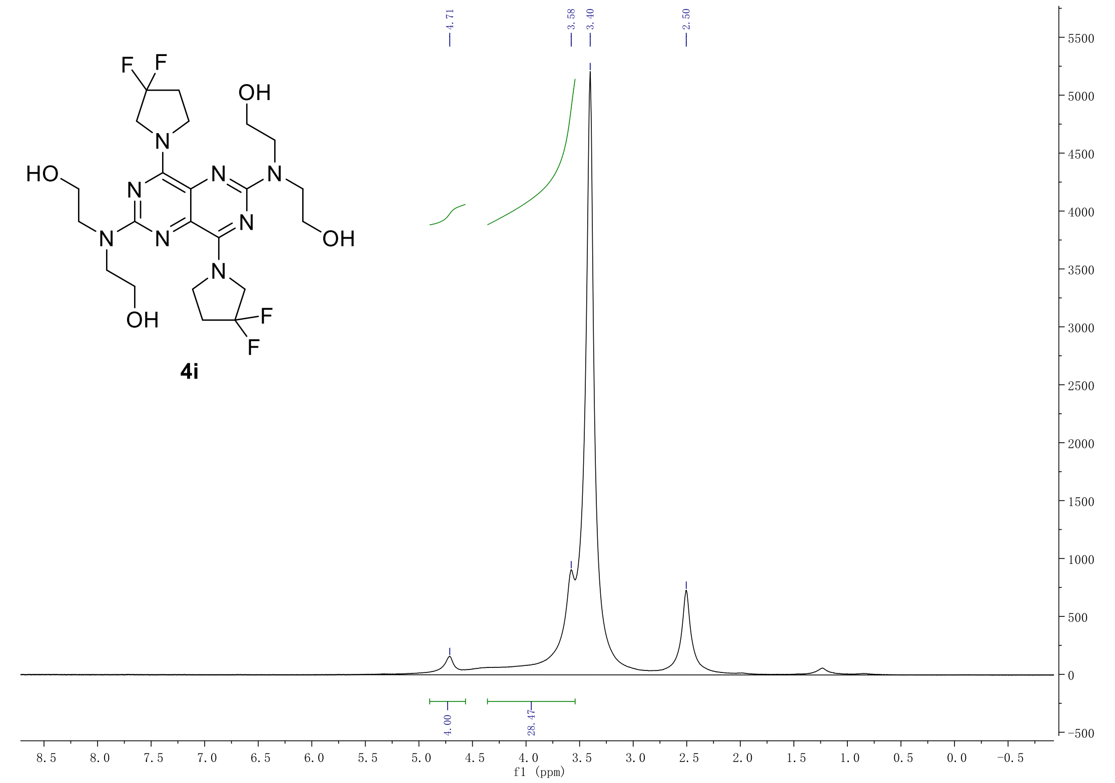
4i
4.71
3.58
3.40
2.50
4.00
28.47
f1 (ppm)

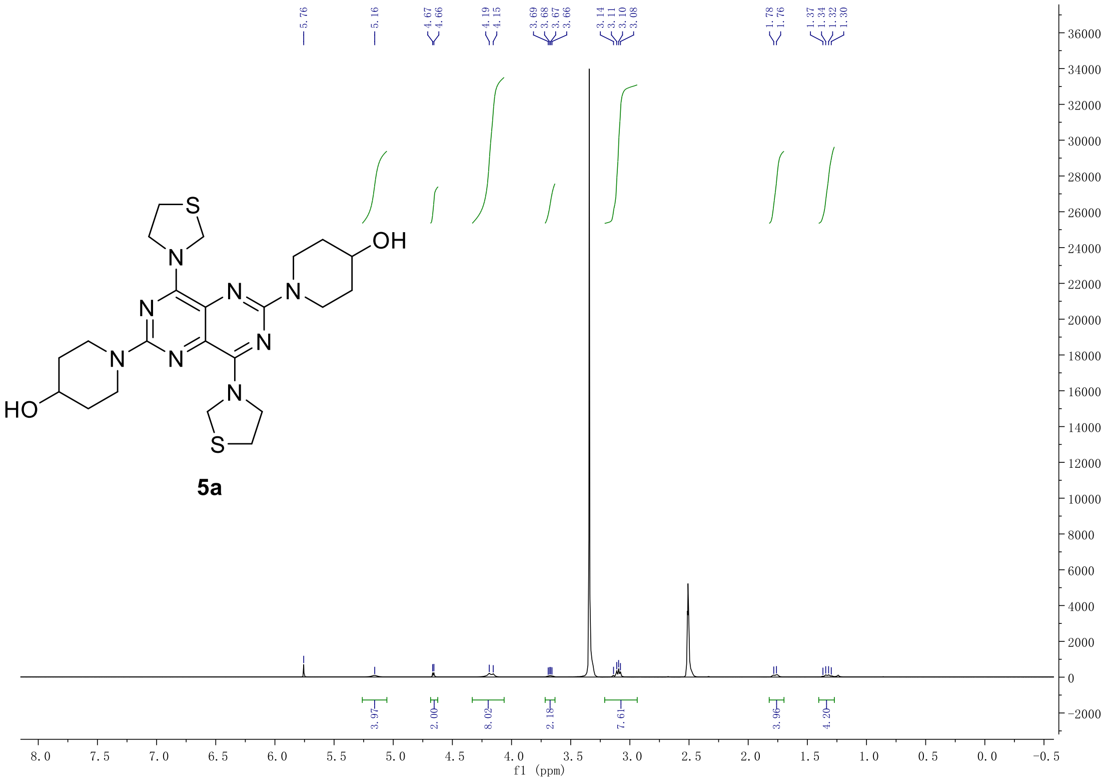
5a
5.76
5.16
4.67
4.66
4.19
4.15
3.69
3.68
3.67
3.66
3.14
3.11
3.10
3.08
1.78
1.76
1.37
1.34
1.32
1.30
3.97
2.00
8.02
2.18
7.61
3.96
4.20
f1 (ppm)

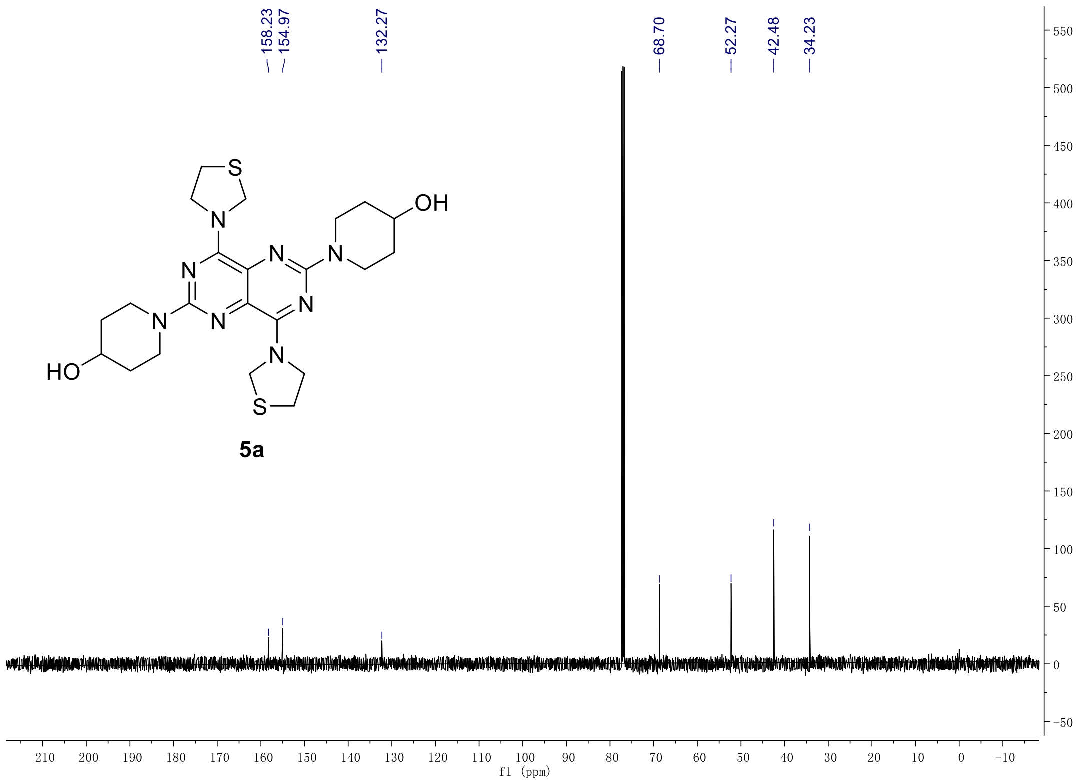
158.23
154.97
132.27
68.70
52.27
42.48
34.23
5a
f1 (ppm)

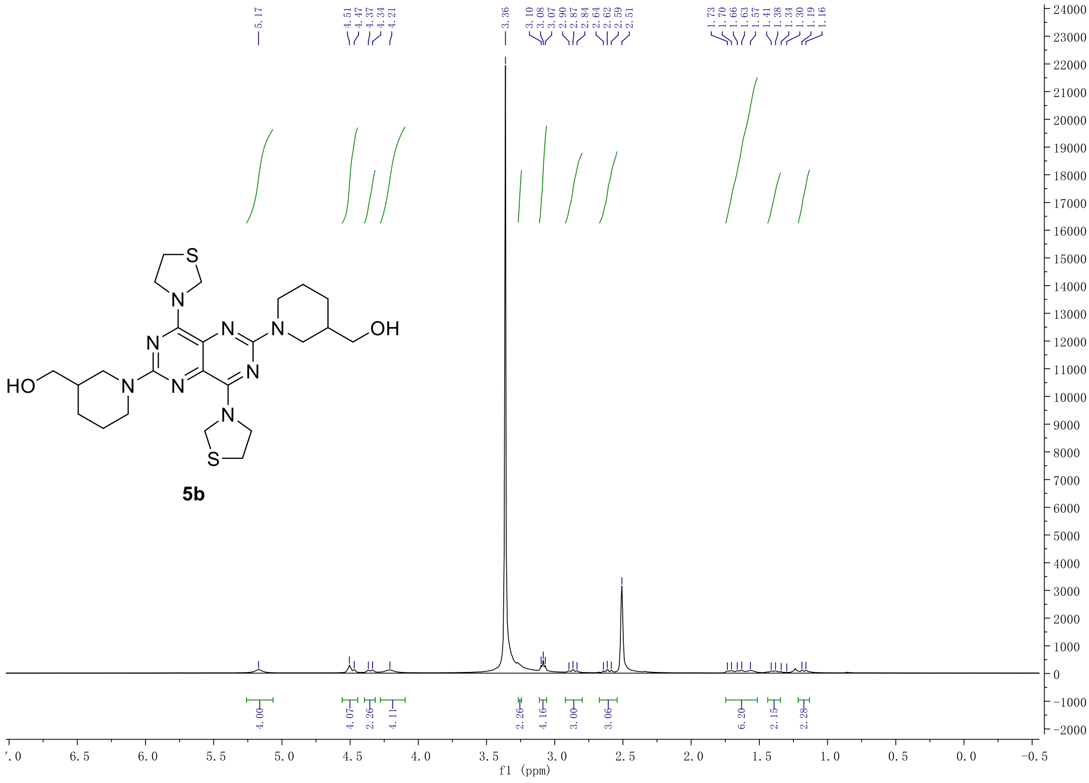
5.17
4.51 4.47 4.37 4.34 4.21
3.36
3.10 3.08 3.07 2.90 2.87 2.84 2.64 2.62 2.59 2.51
1.73 1.70 1.66 1.63 1.57 1.41 1.38 1.34 1.30 1.19 1.16
4.00
4.07
2.26
4.11
2.26
4.16
3.00
3.06
6.20
2.15
2.28
5b
f1 (ppm)

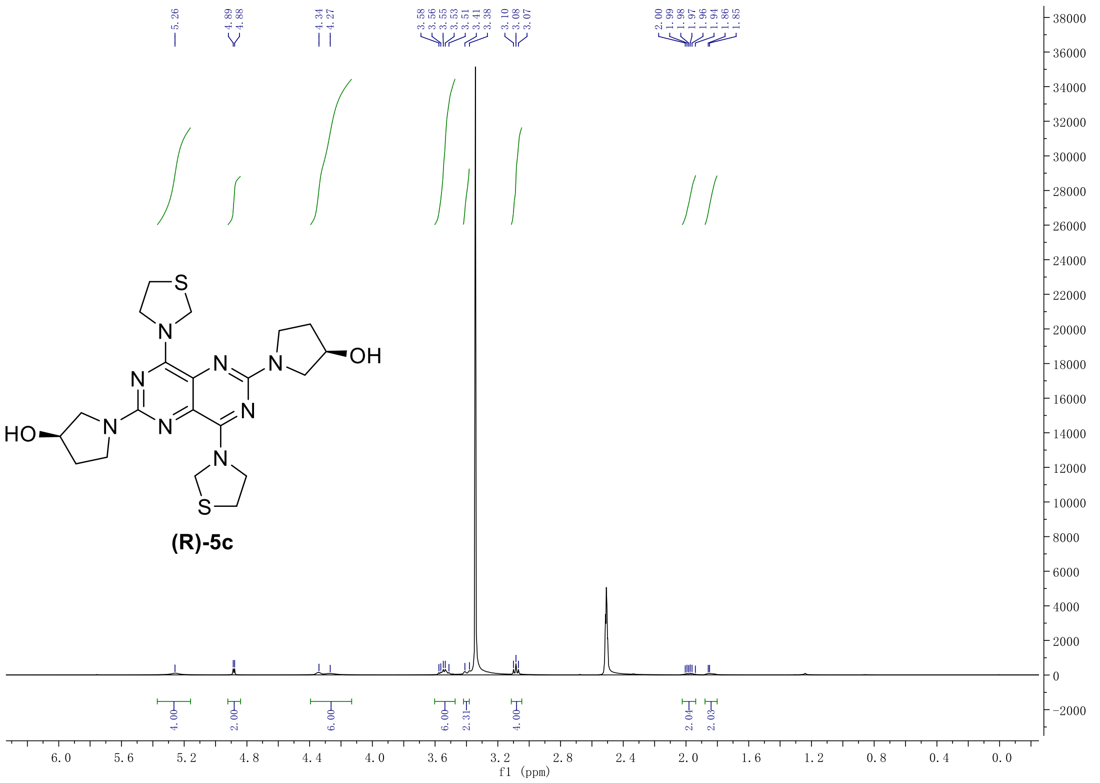

5.26
4.89
4.88
4.34
4.27
3.58
3.56
3.55
3.53
3.51
3.41
3.38
3.10
3.08
3.07
2.00
1.99
1.98
1.97
1.96
1.94
1.86
1.85
4.00
2.00
6.00
6.00
2.31
4.00
2.04
2.03
(R)-5c
f1 (ppm)

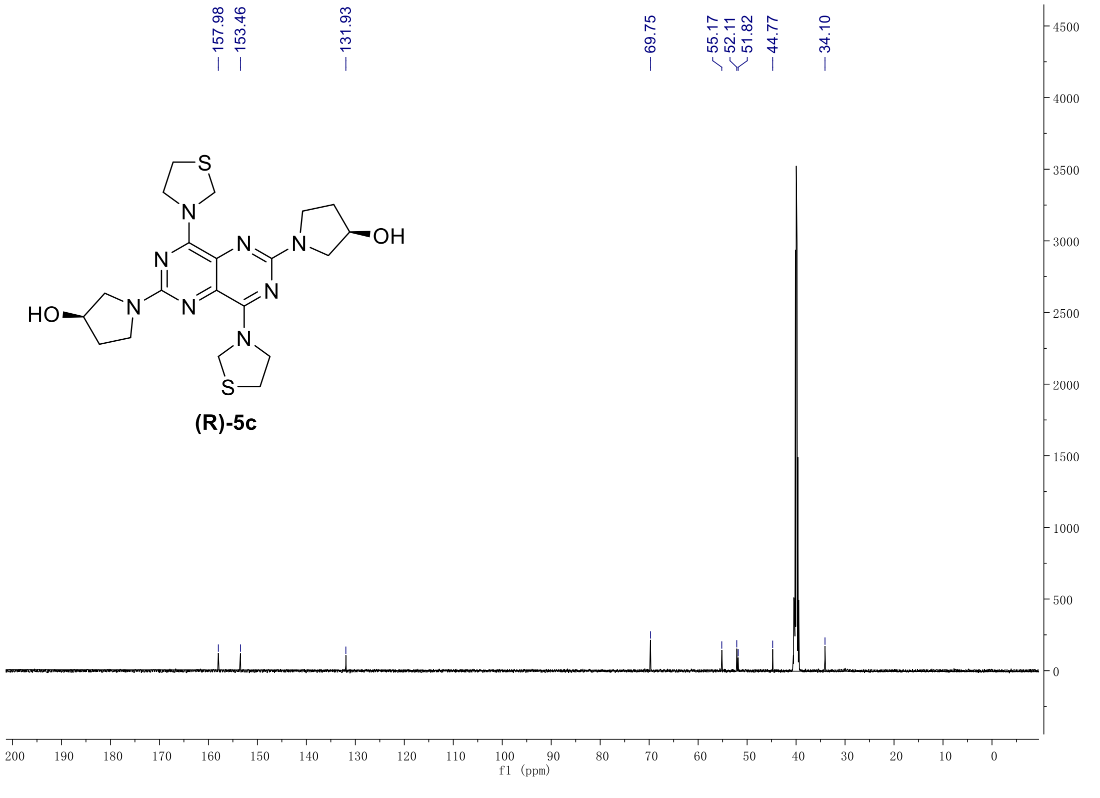

157.98
153.46
131.93
69.75
55.17
52.11
51.82
44.77
34.10
(R)-5c
f1 (ppm)

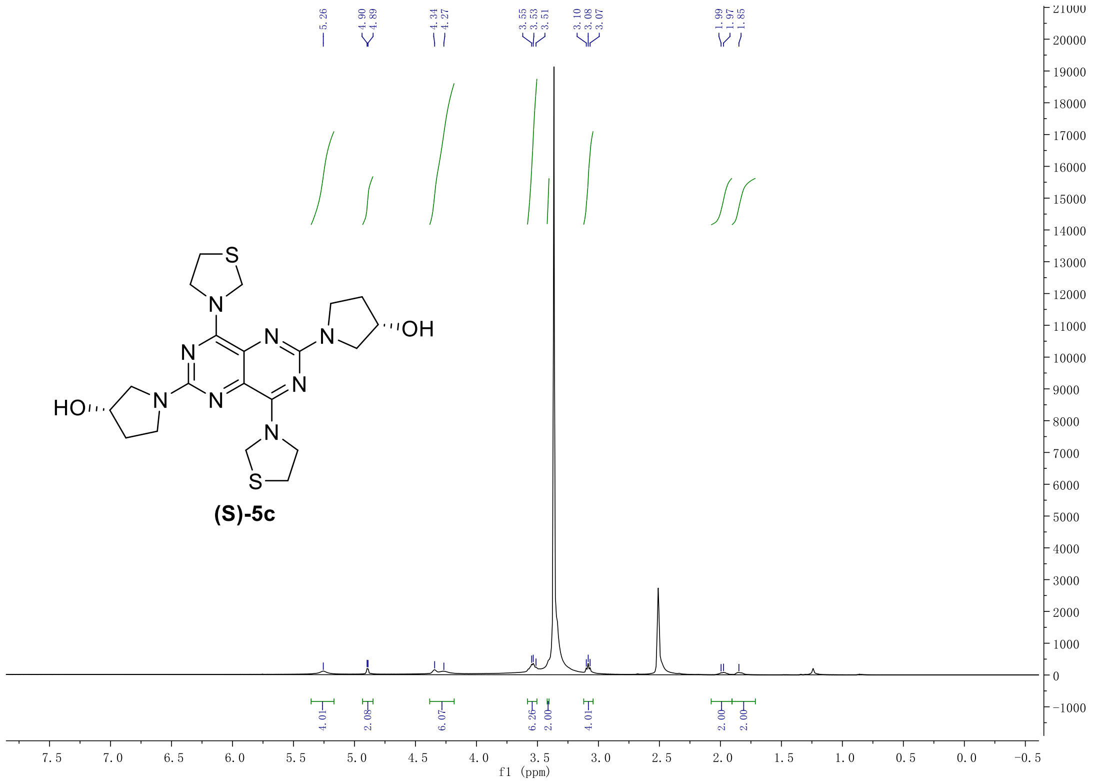

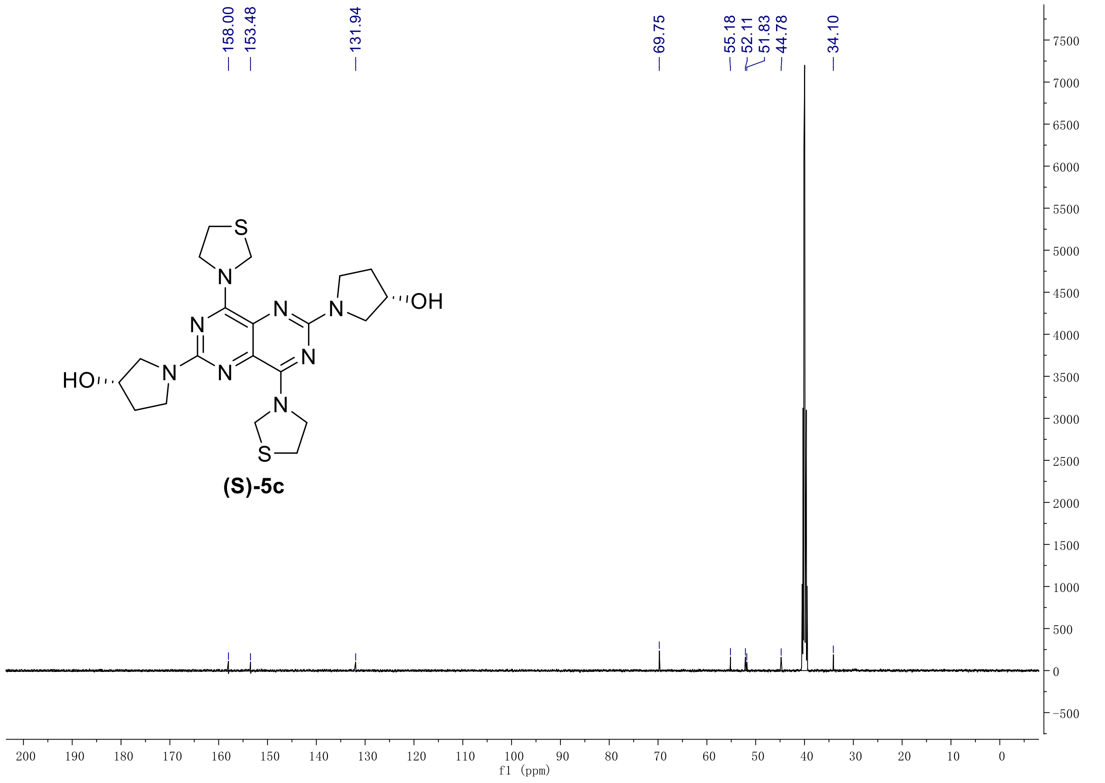

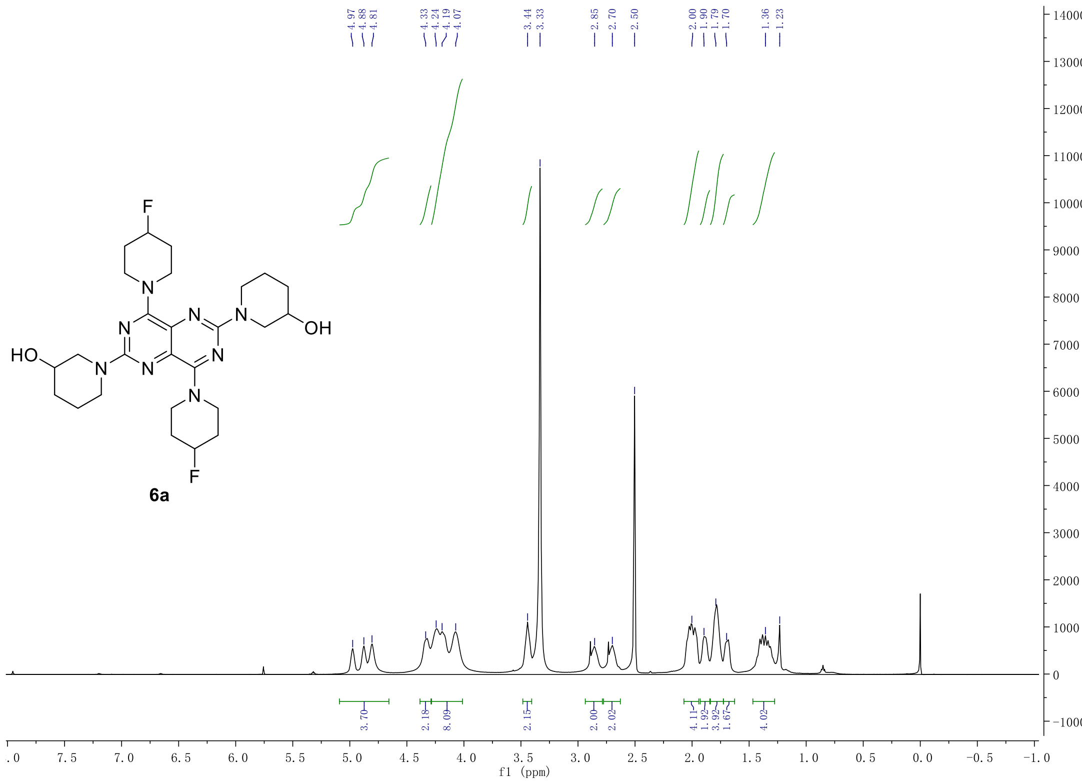
4.97
4.88
4.81
4.33
4.24
4.19
4.07
3.44
3.33
2.85
2.70
2.50
2.00
1.90
1.79
1.70
1.36
1.23
3.70
2.18
8.09
2.15
2.00
2.02
4.11
1.92
3.92
1.67
4.02
F
N
HO
OH
6a
f1 (ppm)

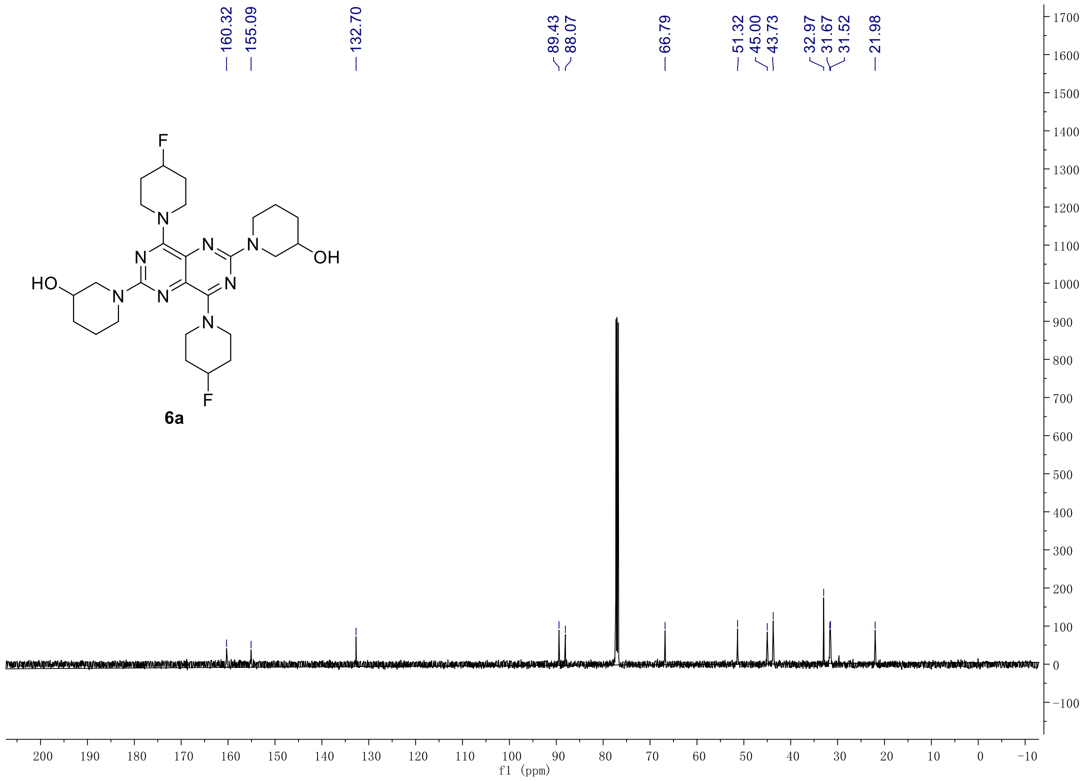
160.32
155.09
132.70
89.43
88.07
66.79
51.32
45.00
43.73
32.97
31.67
31.52
21.98
F
N
HO
OH
6a
f1 (ppm)

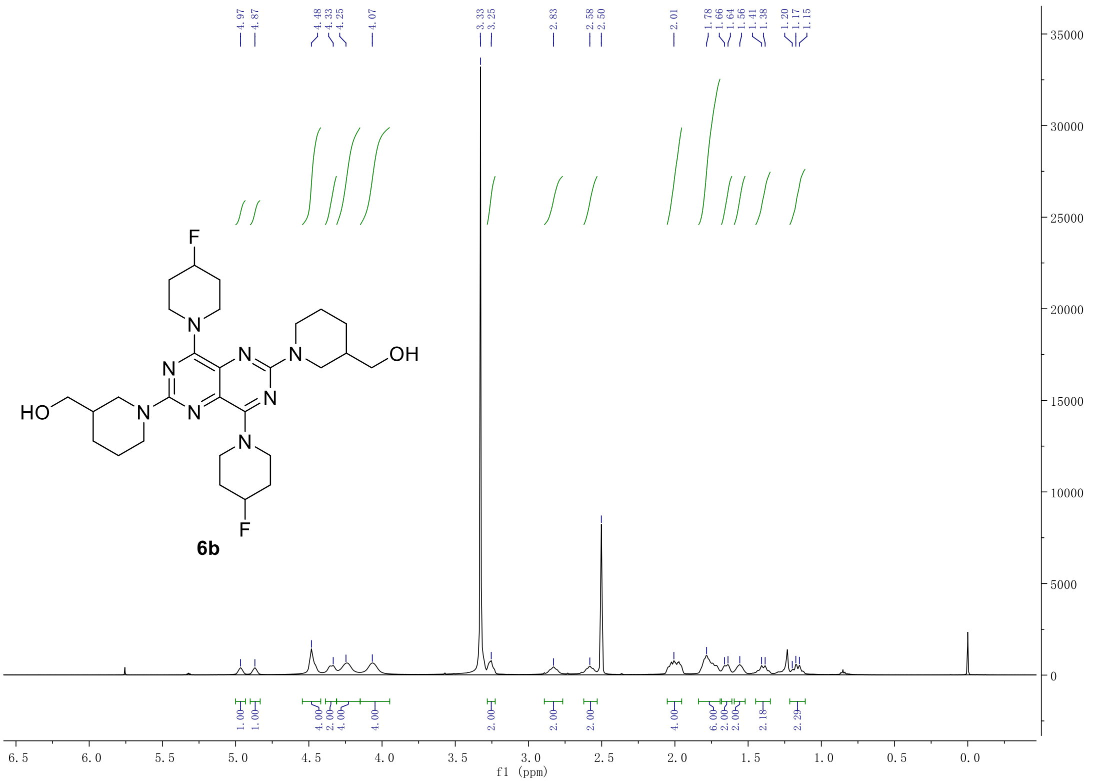

HO
OH
F
F
N
6b
f1 (ppm)

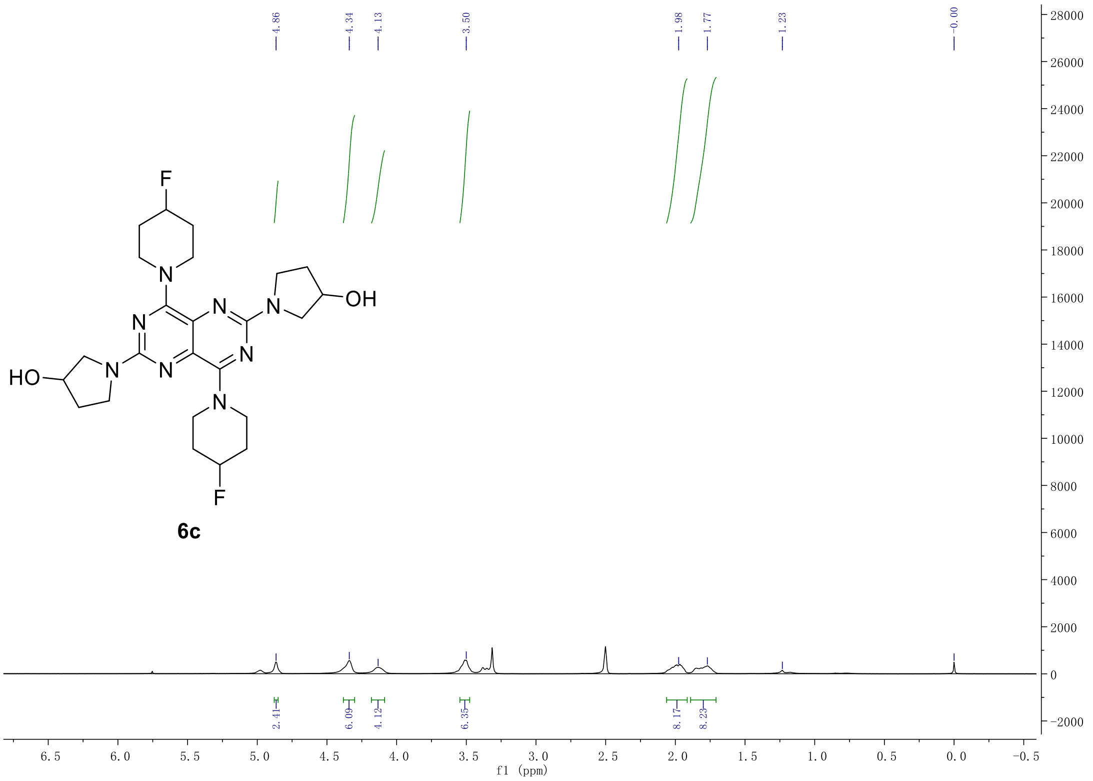

HO
OH
F
F
N
6c
f1 (ppm)

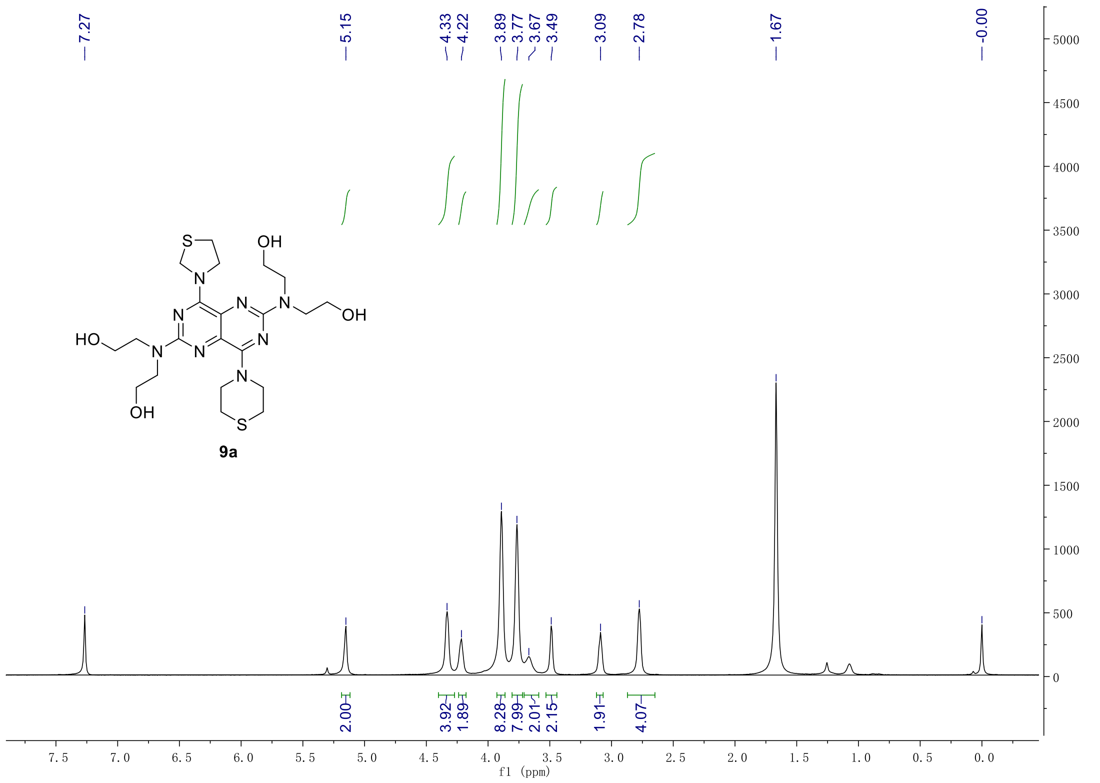
7.27
5.15
4.33
4.22
3.89
3.77
3.67
3.49
3.09
2.78
1.67
-0.00
2.00
3.92
1.89
8.28
7.99
2.01
2.15
1.91
4.07
9a
f1 (ppm)

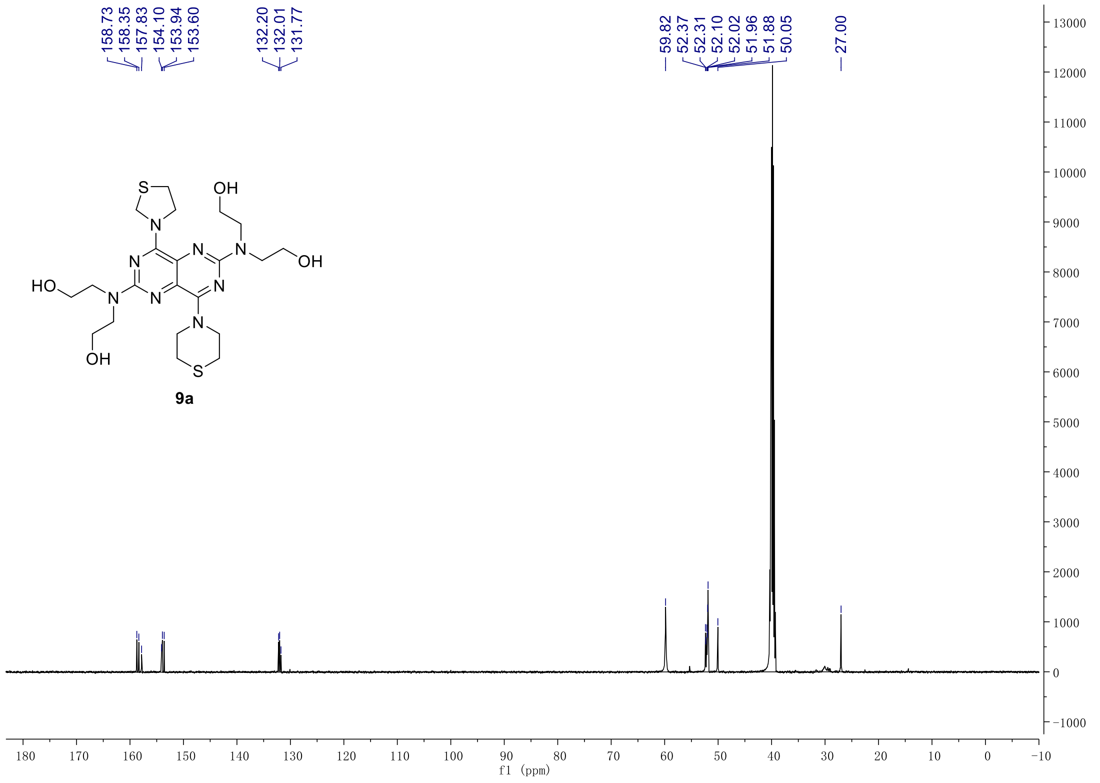
158.73
158.35
157.83
154.10
153.94
153.60
132.20
132.01
131.77
59.82
52.37
52.31
52.10
52.02
51.96
51.88
50.05
27.00
9a
f1 (ppm)

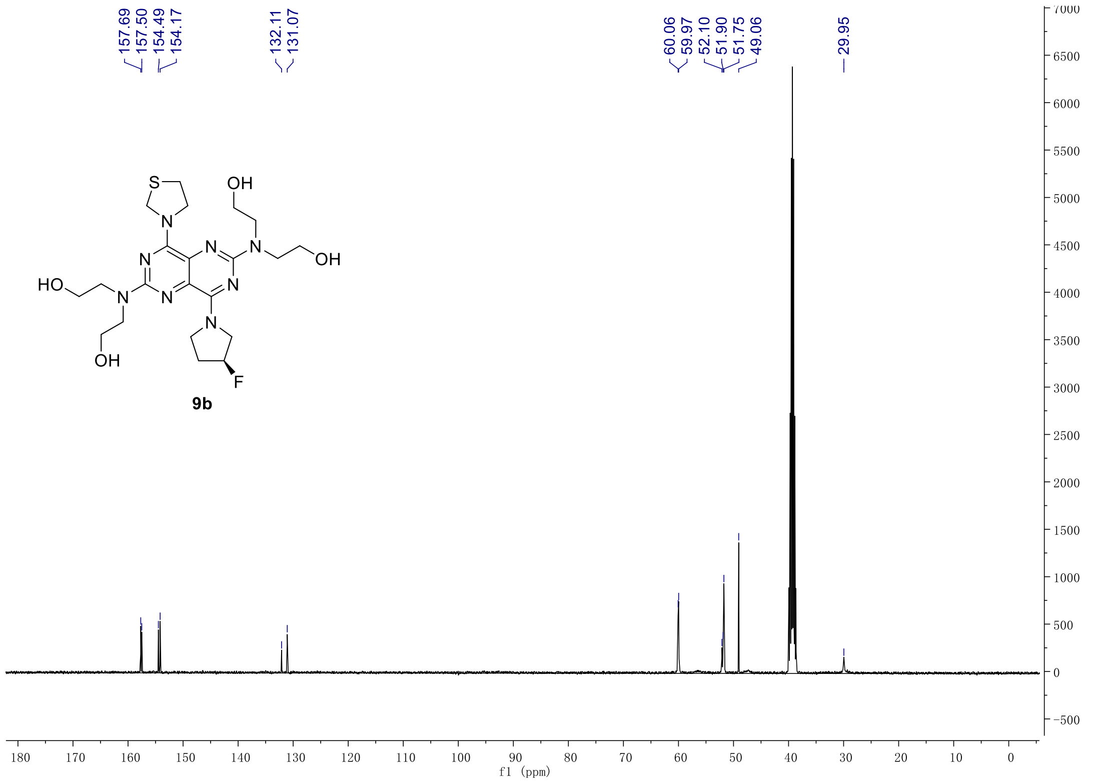

157.69
157.50
154.49
154.17
132.11
131.07
60.06
59.97
52.10
51.90
51.75
49.06
29.95
9b
f1 (ppm)

5. HRMS spectra of the target compounds.

Elmt	Val.	Min	Max	Elmt	Val.	Min	Max	Use Adduct
H	1	0	40	F	1	0	0	H
C	4	0	26	S	2	0	0	Na
N	3	0	10	Cl	1	0	0	K
O	2	1	6					NH4

Error Margin (ppm):	20	DBE Range:	-100.0 - 200.0	Electron Ions:	odd
HC Ratio:	0.0 - 100.0	Apply N Rule:	yes	Use MSn Info:	no
Max Isotopes:	all	Isotope RI (%):	1.00	Isotope Res:	10000
MSn Iso RI (%):	75.00	MSn Logic Mode:	OR	Max Results:	1000

Event#: 1 MS(E+) Ret. Time : 0.890 Scan# : 133

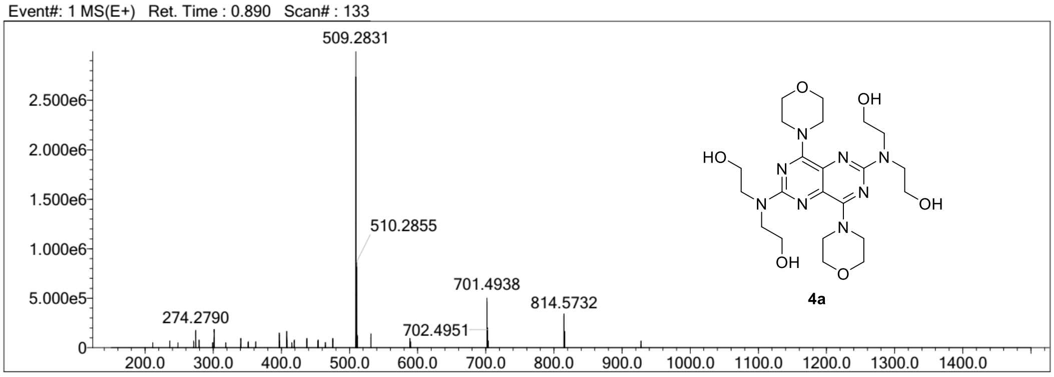

4a

Measured region for 509.2831 m/z

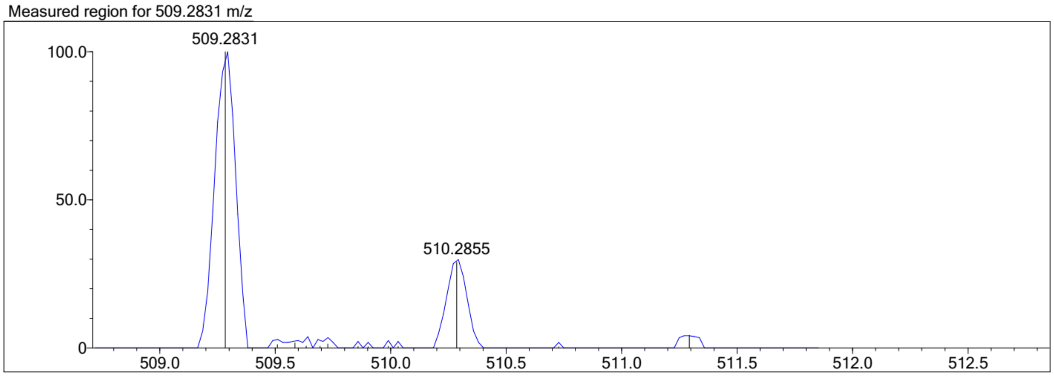

C22 H36 N8 O6 [M+H]+ : Predicted region for 509.2831 m/z

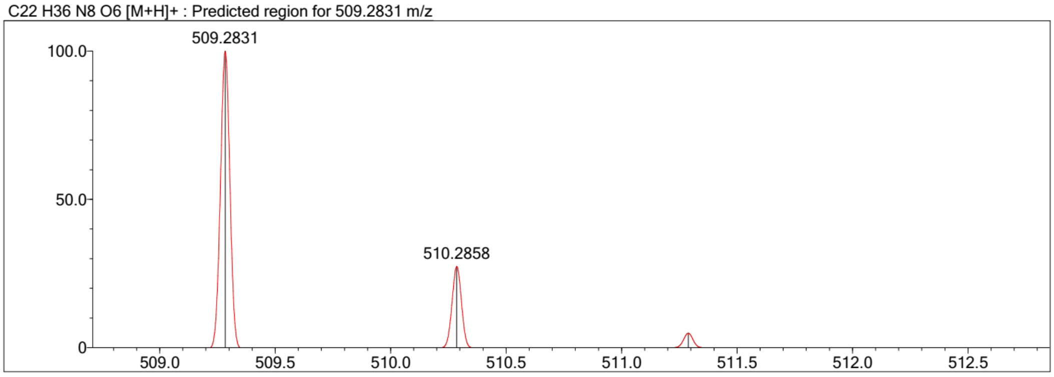

Rank	Score	Formula (M)	Ion	Meas. m/z	Pred. m/z	Df. (mDa)	Df. (ppm)	Iso	DBE
1	96.03	C22 H36 N8 O6	[M+H]+	509.2831	509.2831	0.0	0.00	96.03	9.0

Elmt	Val.	Min	Max	Elmt	Val.	Min	Max	Use Adduct
H	1	0	40	F	1	0	0	H
C	4	0	26	S	2	0	4	Na
N	3	0	10	Cl	1	0	0	K
O	2	1	6					NH4

Error Margin (ppm):	20	DBE Range:	-100.0 - 200.0	Electron Ions:	odd
HC Ratio:	0.0 - 100.0	Apply N Rule:	yes	Use MSn Info:	no
Max Isotopes:	all	Isotope RI (%):	1.00	Isotope Res:	10000
MSn Iso RI (%):	75.00	MSn Logic Mode:	OR	Max Results:	1000

Event#: 1 MS(E+) Ret. Time : 0.810 Scan# : 121

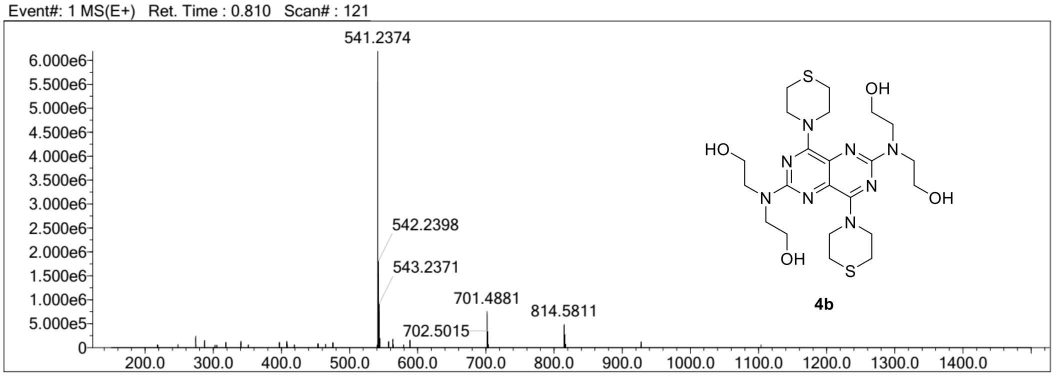

Measured region for 541.2374 m/z

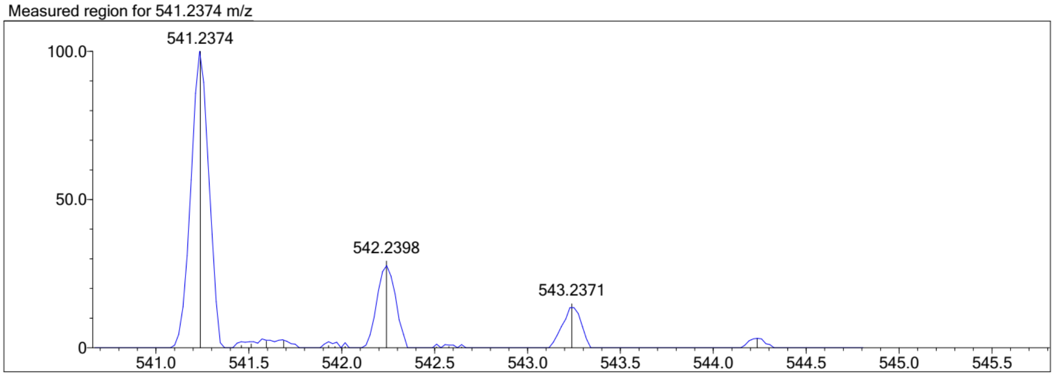

C22 H36 N8 O4 S2 [M+H]+ : Predicted region for 541.2374 m/z

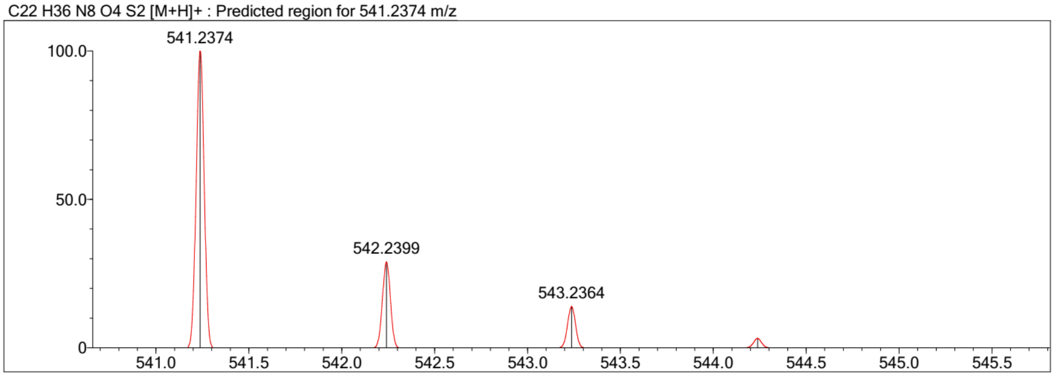

Rank	Score	Formula (M)	Ion	Meas. m/z	Pred. m/z	Df. (mDa)	Df. (ppm)	Iso	DBE
1	93.93	C22 H36 N8 O4 S2	[M+H]+	541.2374	541.2374	0.0	0.00	93.93	9.0

Elmt	Val.	Min	Max	Elmt	Val.	Min	Max	Use Adduct
H	1	0	45	F	1	0	5	H
C	4	0	26	S	2	0	0	Na
N	3	0	10	Cl	1	0	0	K
O	2	0	8					NH4

Error Margin (ppm):	20	DBE Range:	-100.0 - 200.0	Electron Ions:	odd
HC Ratio:	0.0 - 100.0	Apply N Rule:	yes	Use MSn Info:	no
Max Isotopes:	all	Isotope RI (%):	1.00	Isotope Res:	10000
MSn Iso RI (%):	75.00	MSn Logic Mode:	OR	Max Results:	1000

Event#: 1 MS(E+) Ret. Time : 0.517 Scan# : 77

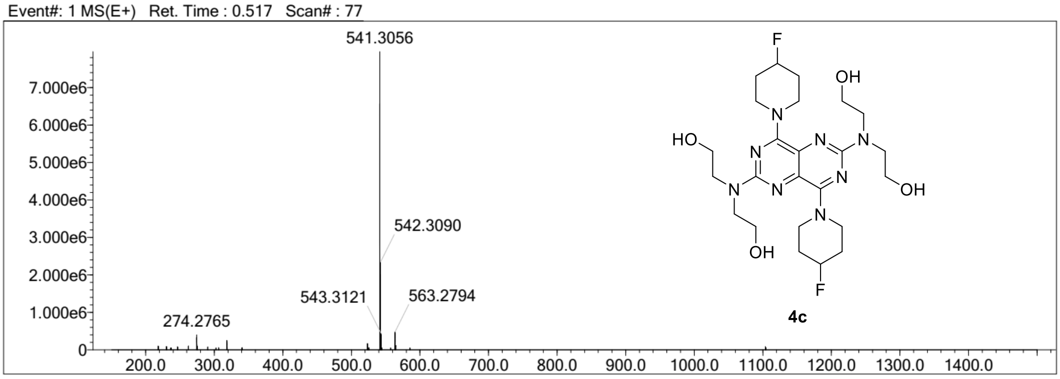

Measured region for 541.3056 m/z

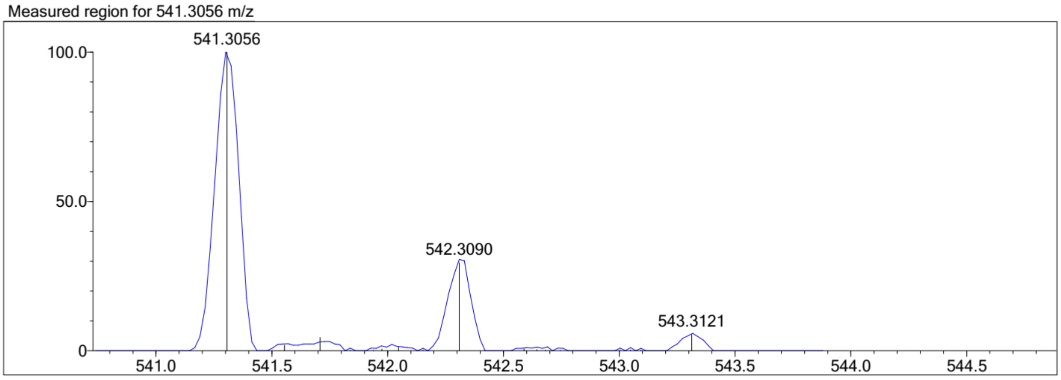

C24 H38 N8 O4 F2 [M+H]+ : Predicted region for 541.3057 m/z

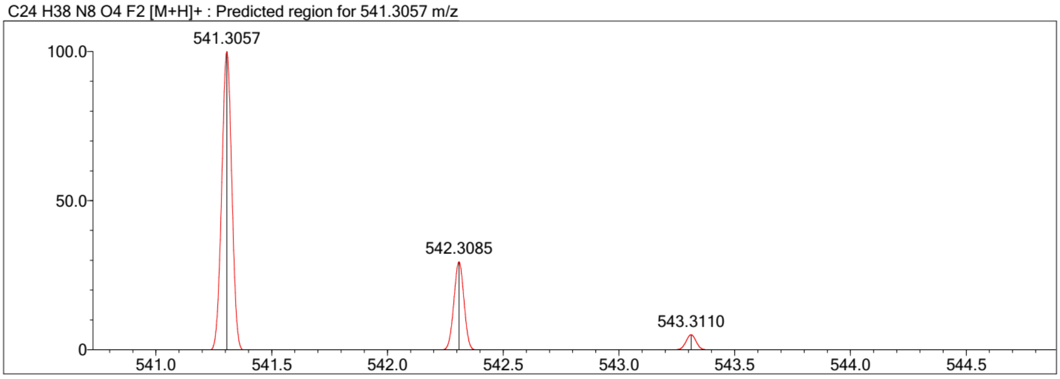

Rank	Score	Formula (M)	Ion	Meas. m/z	Pred. m/z	Df. (mDa)	Df. (ppm)	Iso	DBE
1	82.08	C24 H38 N8 O4 F2	[M+H]+	541.3056	541.3057	-0.1	-0.18	82.08	9.0

Elmt	Val.	Min	Max	Elmt	Val.	Min	Max	Use Adduct
H	1	0	45	F	1	2	5	H
C	4	0	26	S	2	0	0	Na
N	3	0	10	Cl	1	0	0	K
O	2	0	6					NH4

Error Margin (ppm):	20	DBE Range:	-100.0 - 200.0	Electron Ions:	odd
HC Ratio:	0.0 - 100.0	Apply N Rule:	yes	Use MSn Info:	no
Max Isotopes:	all	Isotope RI (%):	1.00	Isotope Res:	10000
MSn Iso RI (%):	75.00	MSn Logic Mode:	OR	Max Results:	1000

Event#: 1 MS(E+) Ret. Time : 1.037 Scan# : 155

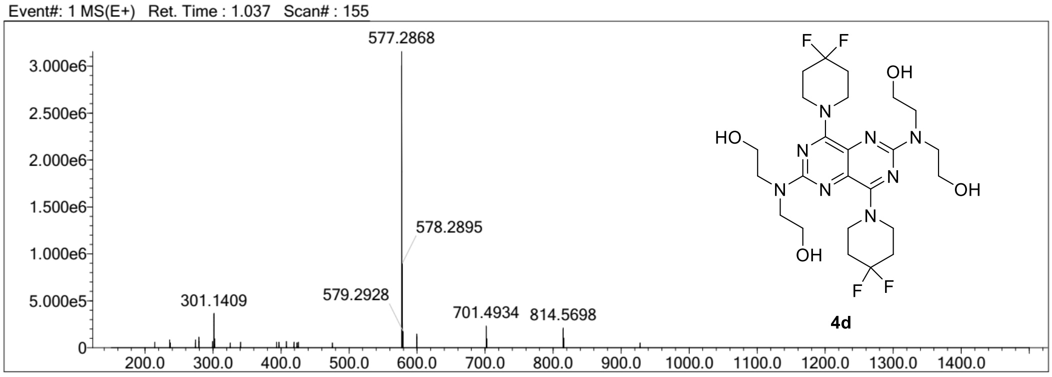

Measured region for 577.2868 m/z

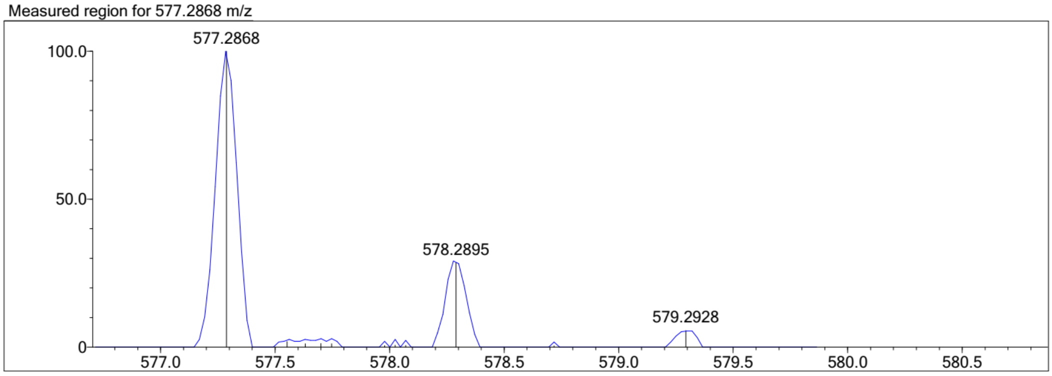

C24 H36 N8 O4 F4 [M+H]+ : Predicted region for 577.2868 m/z

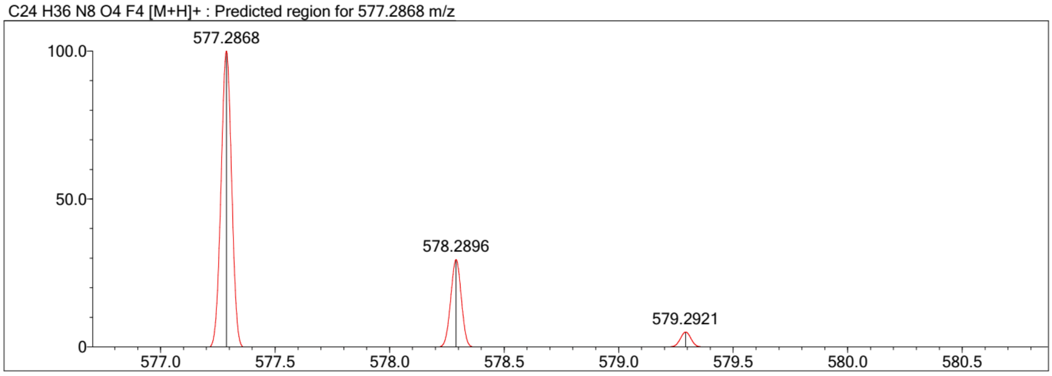

Rank	Score	Formula (M)	Ion	Meas. m/z	Pred. m/z	Df. (mDa)	Df. (ppm)	Iso	DBE
4	89.19	C24 H36 N8 O4 F4	[M+H]+	577.2868	577.2868	-0.0	0.00	89.19	9.0

Elmt	Val.	Min	Max	Elmt	Val.	Min	Max	Use Adduct
H	1	0	40	F	1	0	0	H
C	4	0	26	S	2	0	0	Na
N	3	0	10	Cl	1	0	0	K
O	2	1	6					NH4

Error Margin (ppm):	20	DBE Range:	-100.0 - 200.0	Electron Ions:	odd
HC Ratio:	0.0 - 100.0	Apply N Rule:	yes	Use MSn Info:	no
Max Isotopes:	all	Isotope RI (%):	1.00	Isotope Res:	10000
MSn Iso RI (%):	75.00	MSn Logic Mode:	OR	Max Results:	1000

Event#: 1 MS(E+) Ret. Time : 1.570 Scan# : 235

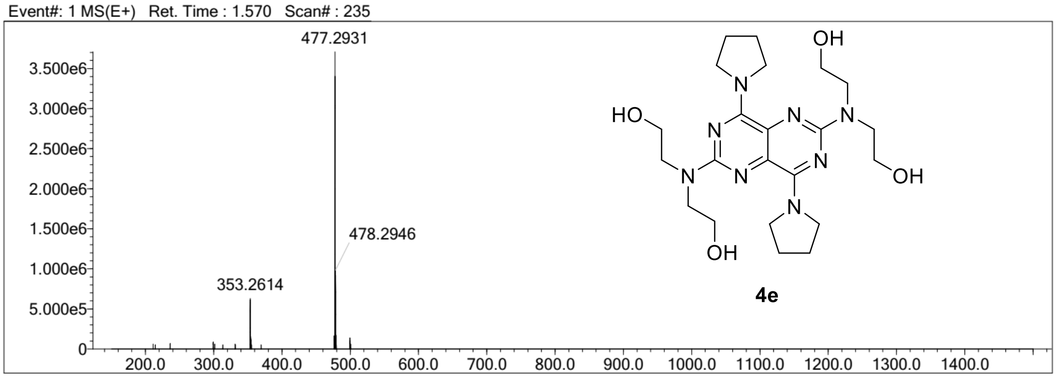

Measured region for 477.2931 m/z

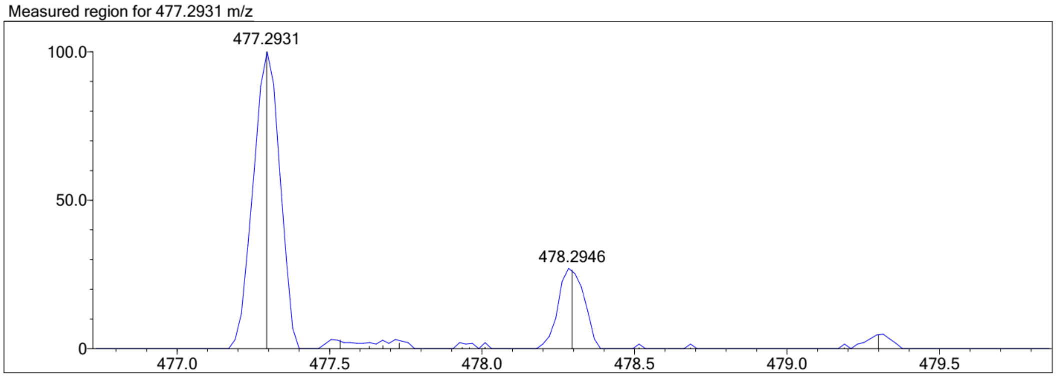

C22 H36 N8 O4 [M+H]+ : Predicted region for 477.2932 m/z

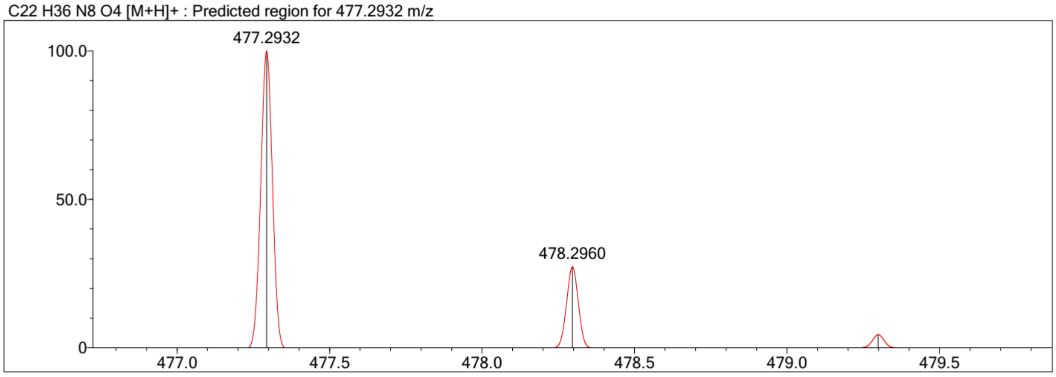

Rank	Score	Formula (M)	Ion	Meas. m/z	Pred. m/z	Df. (mDa)	Df. (ppm)	Iso	DBE
1	88.12	C22 H36 N8 O4	[M+H]+	477.2931	477.2932	-0.1	-0.21	88.12	9.0

Elmt	Val.	Min	Max
H	1	0	40
C	4	0	26
N	3	0	10
O	2	1	6

Elmt	Val.	Min	Max
F	1	0	0
S	2	0	0
Cl	1	0	0

Use Adduct
H
Na
K
NH4

Error Margin (ppm): 20
HC Ratio: 0.0 - 100.0
Max Isotopes: all
MSn Iso RI (%): 75.00

DBE Range: -100.0 - 200.0
Apply N Rule: yes
Isotope RI (%): 1.00
MSn Logic Mode: OR

Electron Ions: odd
Use MSn Info: no
Isotope Res: 10000
Max Results: 1000

Event#: 1 MS(E+) Ret. Time : 1.330 Scan# : 199

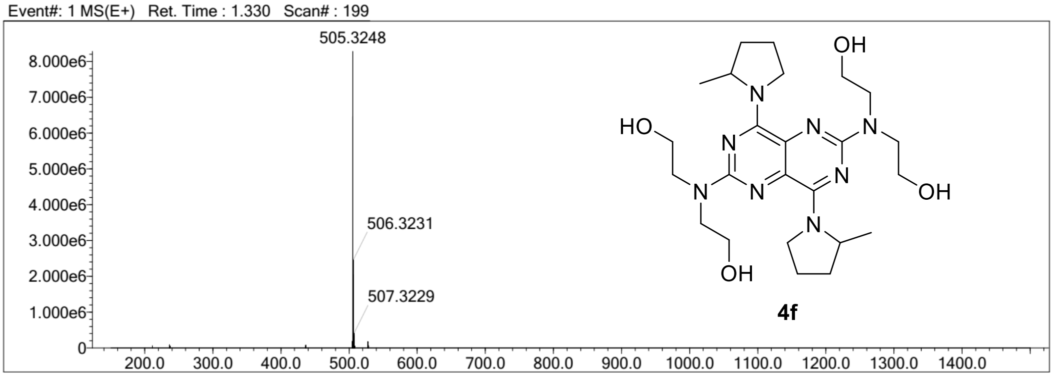

Measured region for 505.3248 m/z

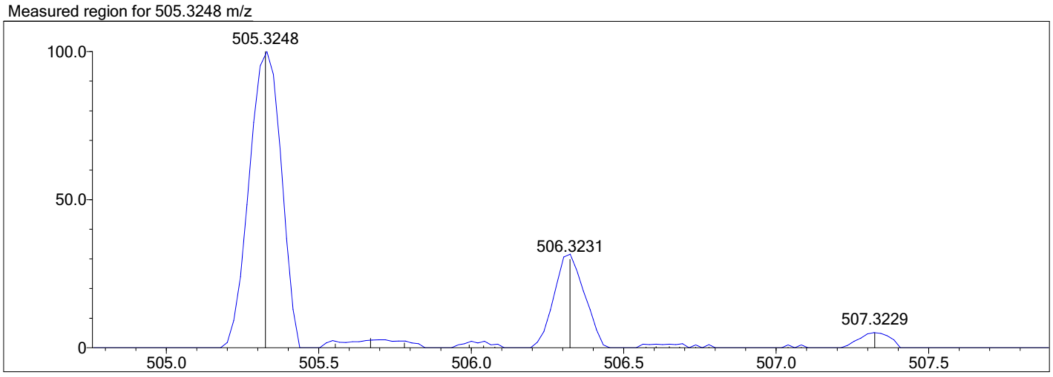

C24 H40 N8 O4 [M+H]+ : Predicted region for 505.3245 m/z

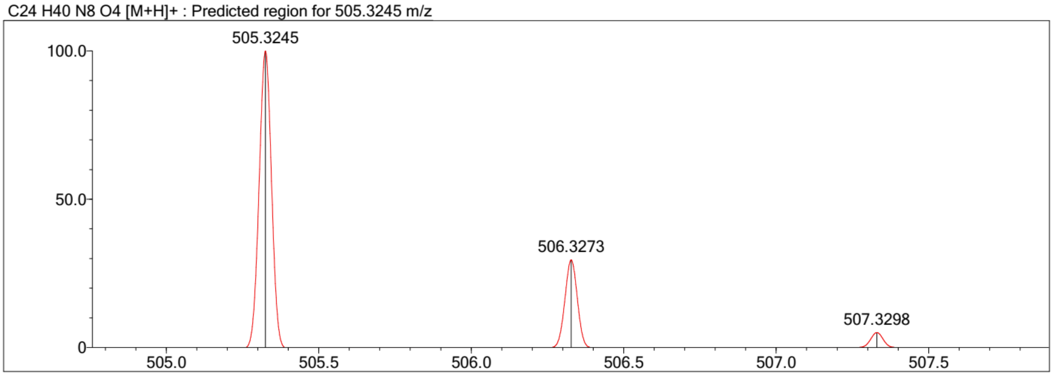

Rank	Score	Formula (M)	Ion	Meas. m/z	Pred. m/z	Df. (mDa)	Df. (ppm)	Iso	DBE
1	80.68	C24 H40 N8 O4	[M+H]+	505.3248	505.3245	0.3	0.59	80.68	9.0

Elmt	Val.	Min	Max	Elmt	Val.	Min	Max	Elmt	Val.	Min	Max	Use Adduct
H	1	0	50	O	2	0	10	Cl	1	0	0	H
C	4	0	32	F	1	0	0	Br	1	0	0	Na
N	3	0	10	S	2	0	0					

Error Margin (ppm):	100	DBE Range:	not fixed	Electron Ions:	both
HC Ratio:	unlimited	Apply N Rule:	no	Use MSn Info:	no
Max Isotopes:	all	Isotope RI (%):	1.00	Isotope Res:	10000
MSn Iso RI (%):	75.00	MSn Logic Mode:	AND	Max Results:	100

Event#: 1 MS(E+) Ret. Time : 0.717 Scan# : 107

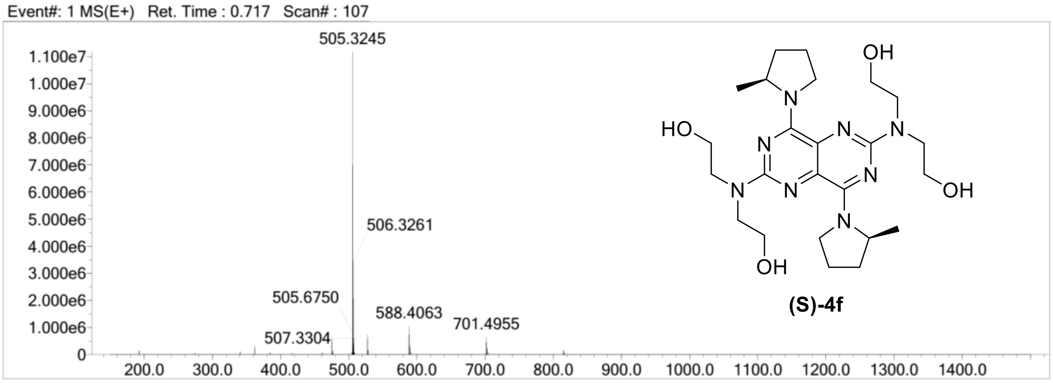

Measured region for 505.3245 m/z

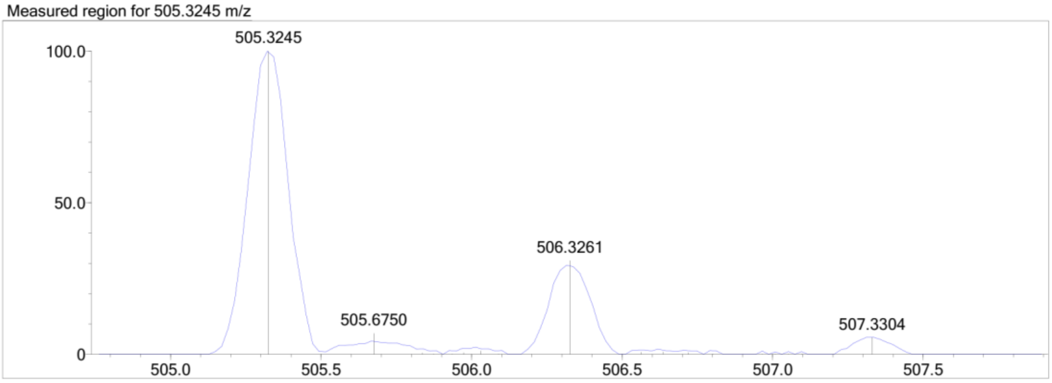

C24 H40 N8 O4 [M+H]+ : Predicted region for 505.3245 m/z

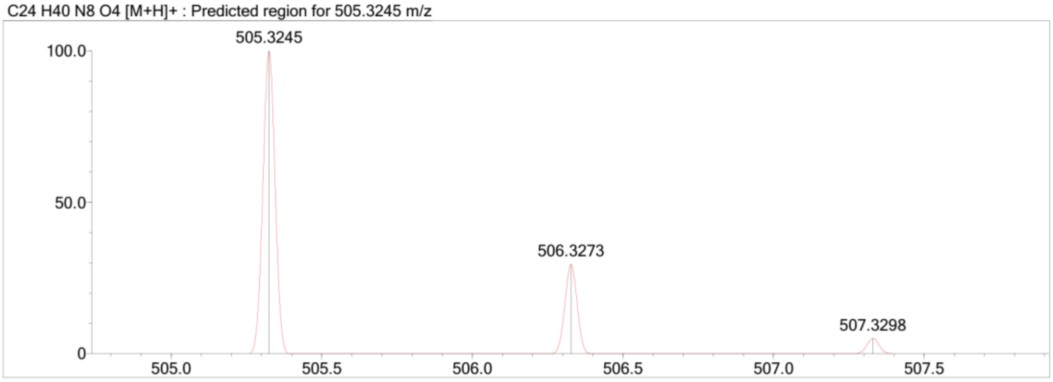

Rank	Score	Formula (M)	Ion	Meas. m/z	Pred. m/z	Df. (mDa)	Df. (ppm)	Iso	DBE
1	71.84	C24 H40 N8 O4	[M+H]+	505.3245	505.3245	-0.0	0.00	71.84	9.0

Elmt	Val.	Min	Max	Elmt	Val.	Min	Max	Elmt	Val.	Min	Max	Use Adduct
H	1	0	50	O	2	0	8	Cl	1	0	0	H
C	4	0	32	F	1	0	0	Br	1	0	0	Na
N	3	0	10	S	2	0	0					

Error Margin (ppm):	100	DBE Range:	not fixed	Electron Ions:	both
HC Ratio:	unlimited	Apply N Rule:	no	Use MSn Info:	no
Max Isotopes:	all	Isotope RI (%):	1.00	Isotope Res:	10000
MSn Iso RI (%):	75.00	MSn Logic Mode:	AND	Max Results:	100

Event#: 1 MS(E+) Ret. Time : 0.610 Scan# : 91

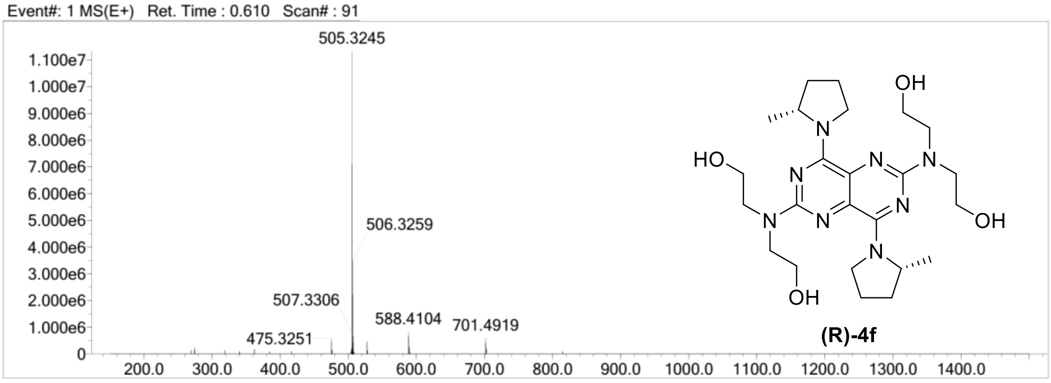

(R)-4f

Measured region for 505.3245 m/z

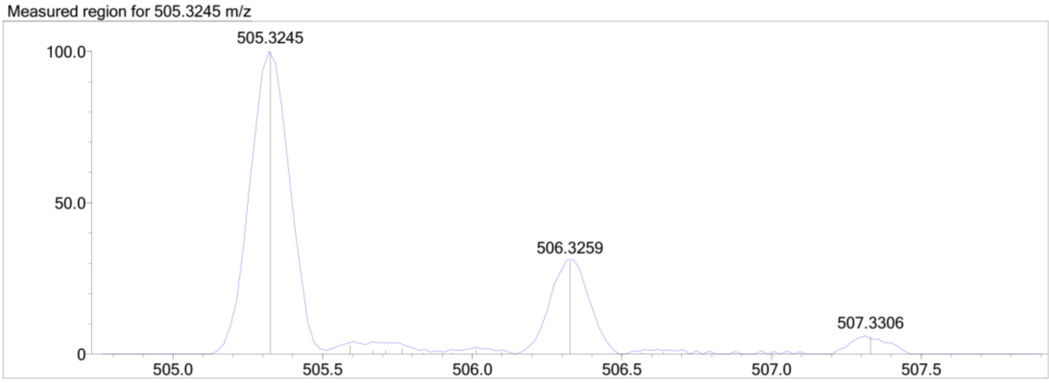

C24 H40 N8 O4 [M+H]+ : Predicted region for 505.3245 m/z

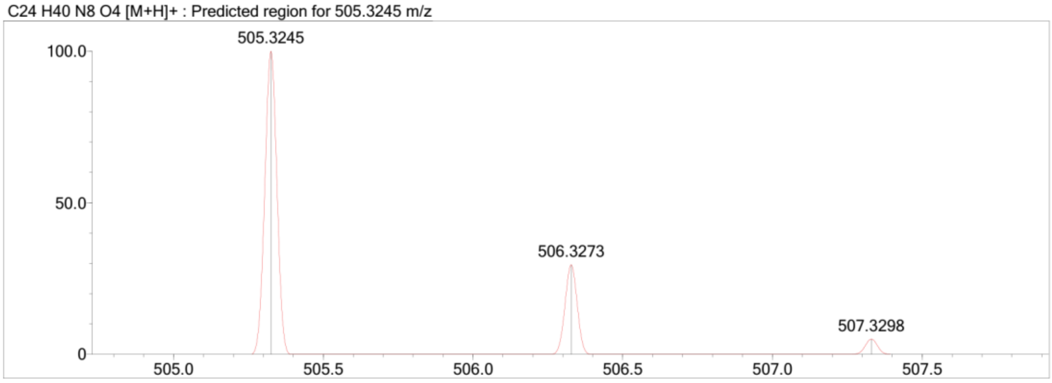

Rank	Score	Formula (M)	Ion	Meas. m/z	Pred. m/z	Df. (mDa)	Df. (ppm)	Iso	DBE
1	70.29	C24 H40 N8 O4	[M+H]+	505.3245	505.3245	-0.0	0.00	70.29	9.0

Elmt	Val.	Min	Max
H	1	0	45
C	4	0	26
N	3	0	10
O	2	0	6

Elmt	Val.	Min	Max
F	1	0	0
S	2	0	5
Cl	1	0	0

Use Adduct
H
Na
K
NH4

Error Margin (ppm):	20	DBE Range:	-100.0 - 200.0	Electron Ions:	odd
HC Ratio:	0.0 - 100.0	Apply N Rule:	yes	Use MSn Info:	no
Max Isotopes:	all	Isotope RI (%):	1.00	Isotope Res:	10000
MSn Iso RI (%):	75.00	MSn Logic Mode:	OR	Max Results:	1000

Event#: 1 MS(E+) Ret. Time : 0.850 Scan# : 127

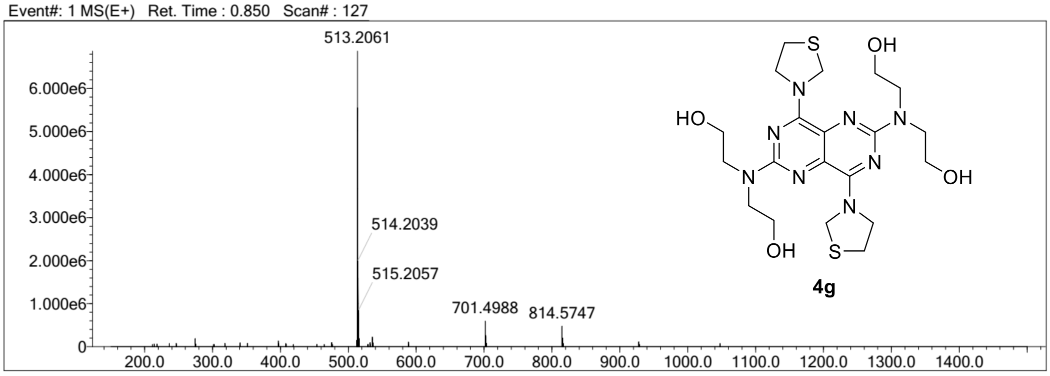

Measured region for 513.2061 m/z

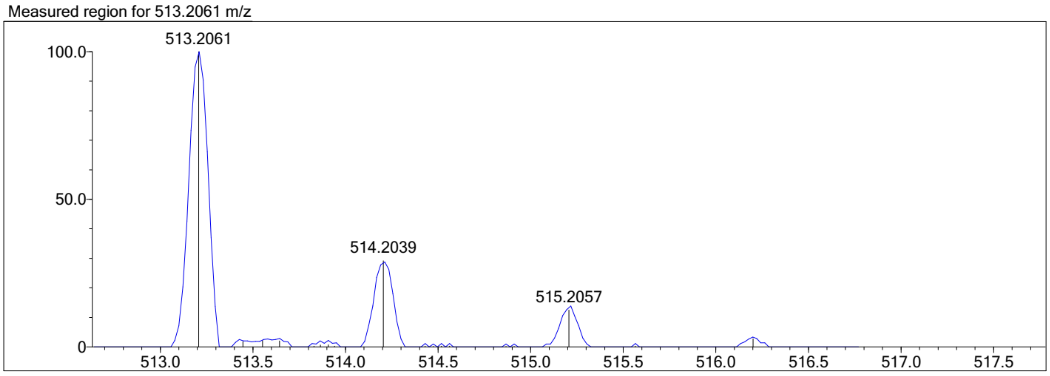

C20 H32 N8 O4 S2 [M+H]+ : Predicted region for 513.2061 m/z

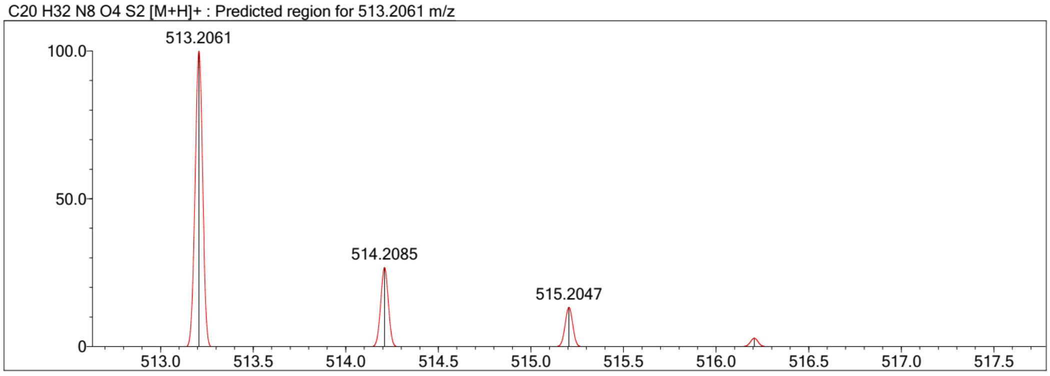

Rank	Score	Formula (M)	Ion	Meas. m/z	Pred. m/z	Df. (mDa)	Df. (ppm)	Iso	DBE
1	86.91	C20 H32 N8 O4 S2	[M+H]+	513.2061	513.2061	0.0	0.00	86.91	9.0

Elmt	Val.	Min	Max	Elmt	Val.	Min	Max	Use Adduct
H	1	0	45	F	1	0	5	H
C	4	0	26	S	2	0	0	Na
N	3	0	10	Cl	1	0	0	K
O	2	0	8					NH4

Error Margin (ppm):	20	DBE Range:	-100.0 - 200.0	Electron Ions:	odd
HC Ratio:	0.0 - 100.0	Apply N Rule:	yes	Use MSn Info:	no
Max Isotopes:	all	Isotope RI (%):	1.00	Isotope Res:	10000
MSn Iso RI (%):	75.00	MSn Logic Mode:	OR	Max Results:	1000

Event#: 1 MS(E+) Ret. Time : 0.930 Scan# : 139

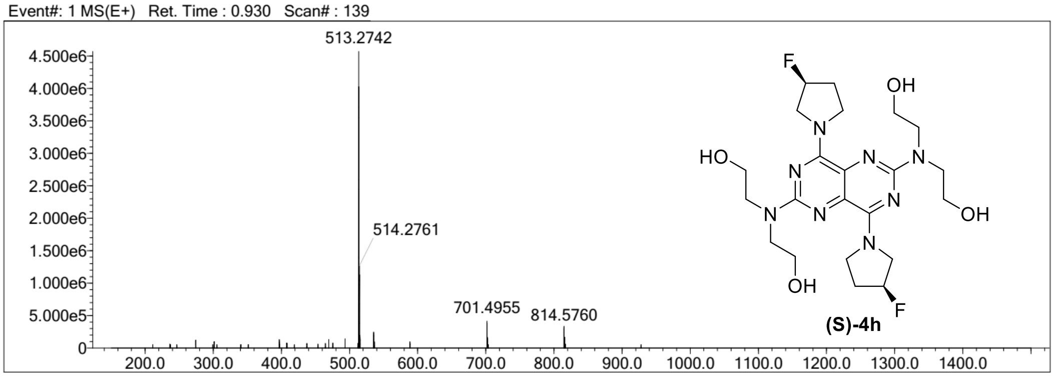

Measured region for 513.2742 m/z

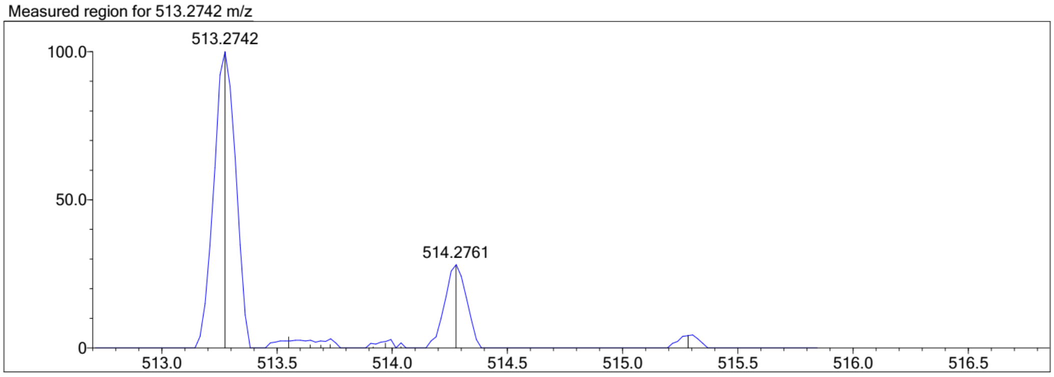

C22 H34 N8 O4 F2 [M+H]+ : Predicted region for 513.2744 m/z

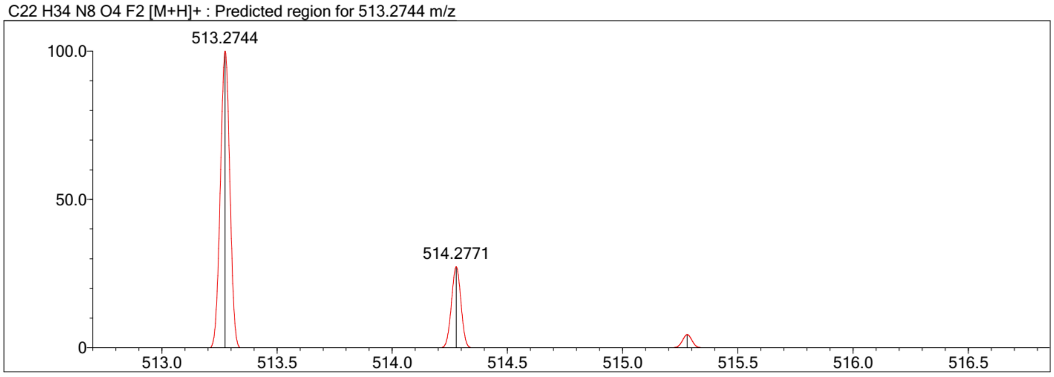

Rank	Score	Formula (M)	Ion	Meas. m/z	Pred. m/z	Df. (mDa)	Df. (ppm)	Iso	DBE
1	83.55	C22 H34 N8 O4 F2	[M+H]+	513.2742	513.2744	-0.2	-0.39	83.55	9.0

Elmt	Val.	Min	Max
H	1	0	45
C	4	0	26
N	3	0	10
O	2	0	8

Elmt	Val.	Min	Max
F	1	0	5
S	2	0	0
Cl	1	0	0

Use Adduct
H
Na
K
NH4

Error Margin (ppm):	20	DBE Range:	-100.0 - 200.0	Electron Ions:	odd
HC Ratio:	0.0 - 100.0	Apply N Rule:	yes	Use MSn Info:	no
Max Isotopes:	all	Isotope RI (%):	1.00	Isotope Res:	10000
MSn Iso RI (%):	75.00	MSn Logic Mode:	OR	Max Results:	1000

Event#: 1 MS(E+) Ret. Time : 1.130 Scan# : 169

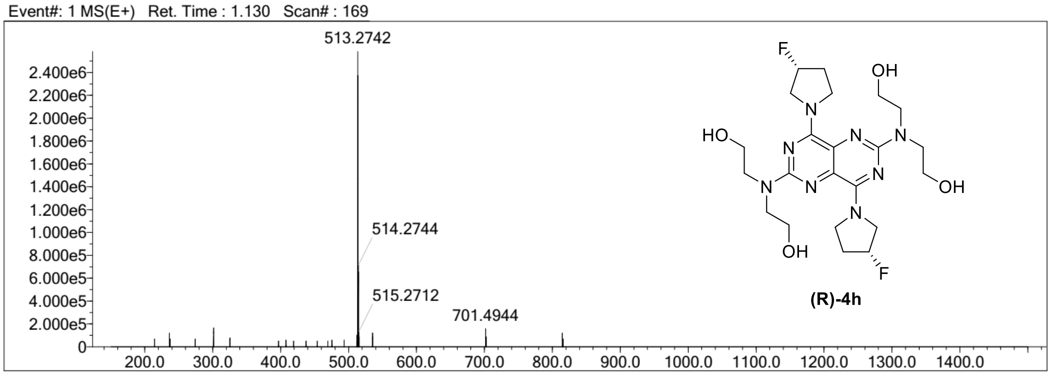

Measured region for 513.2742 m/z

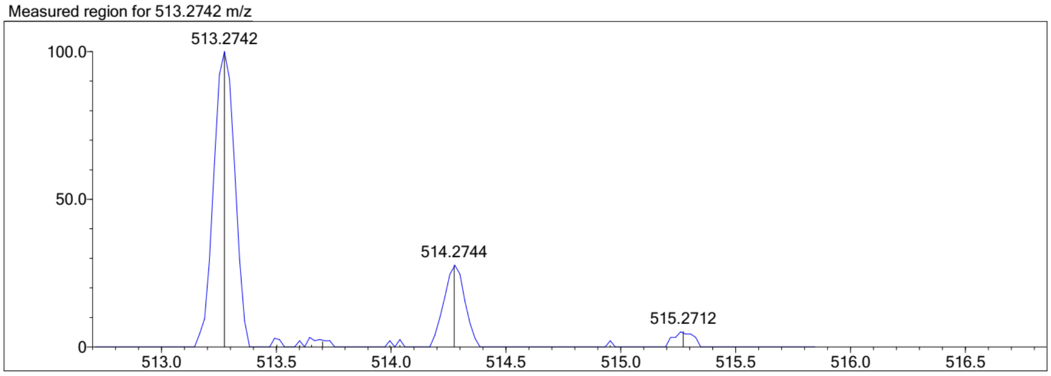

C22 H34 N8 O4 F2 [M+H]+ : Predicted region for 513.2744 m/z

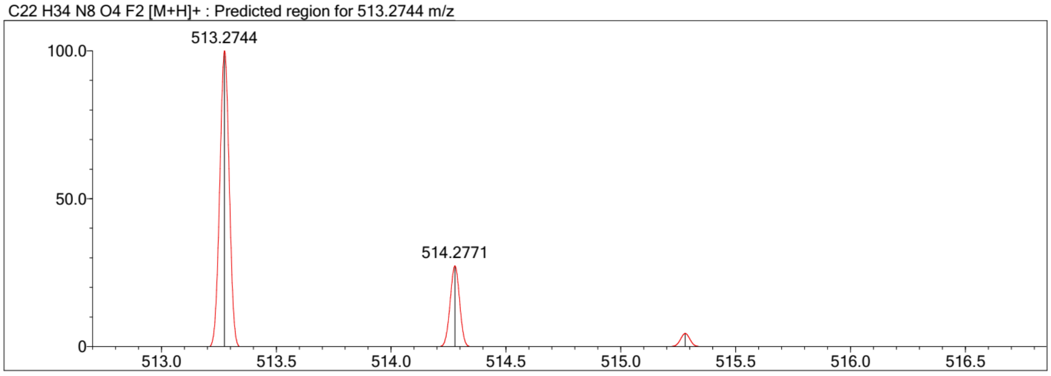

Rank	Score	Formula (M)	Ion	Meas. m/z	Pred. m/z	Df. (mDa)	Df. (ppm)	Iso	DBE
1	100.00	C22 H34 N8 O4 F2	[M+H]+	513.2742	513.2744	-0.2	-0.39	100.00	9.0

Elmt	Val.	Min	Max	Elmt	Val.	Min	Max	Use Adduct
H	1	0	45	F	1	2	5	H
C	4	0	26	S	2	0	0	Na
N	3	0	10	Cl	1	0	0	K
O	2	0	8					NH4

Error Margin (ppm):	20	DBE Range:	-100.0 - 200.0	Electron Ions:	odd
HC Ratio:	0.0 - 100.0	Apply N Rule:	yes	Use MSn Info:	no
Max Isotopes:	all	Isotope RI (%):	1.00	Isotope Res:	10000
MSn Iso RI (%):	75.00	MSn Logic Mode:	OR	Max Results:	1000

Event#: 1 MS(E+) Ret. Time : 0.783 Scan# : 117

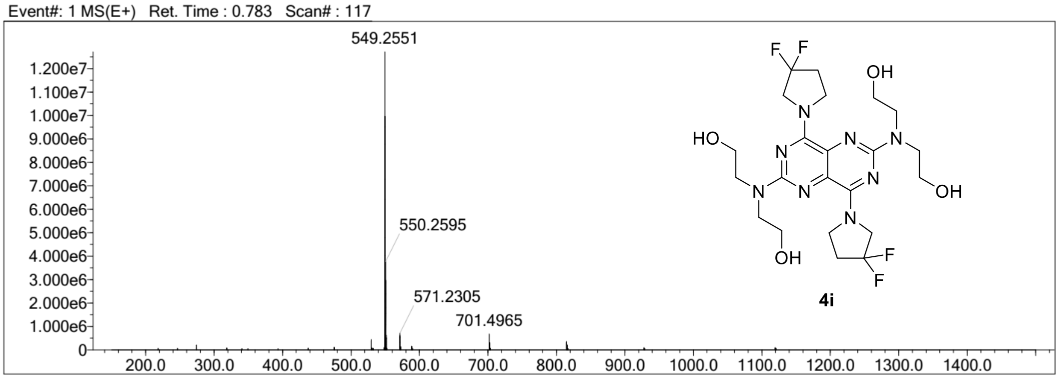

Measured region for 549.2551 m/z

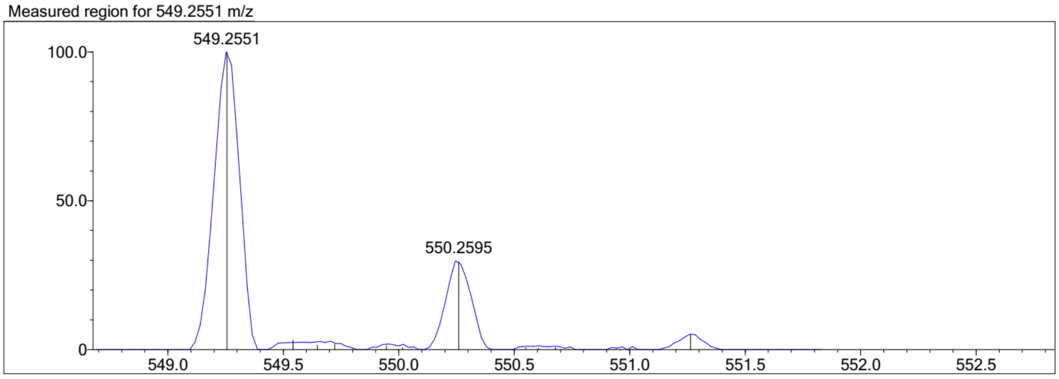

C22 H32 N8 O4 F4 [M+H]+ : Predicted region for 549.2555 m/z

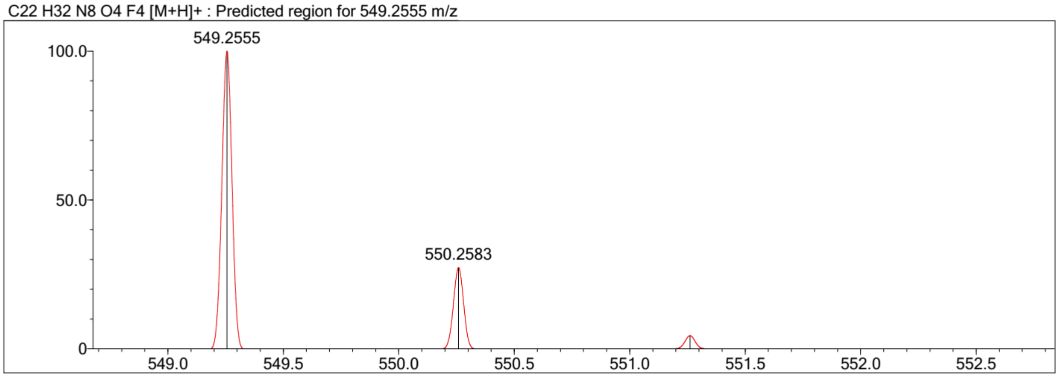

Rank	Score	Formula (M)	Ion	Meas. m/z	Pred. m/z	Df. (mDa)	Df. (ppm)	Iso	DBE
1	83.69	C22 H32 N8 O4 F4	[M+H]+	549.2551	549.2555	-0.4	-0.73	83.69	9.0

Elmt	Val.	Min	Max
H	1	0	45
C	4	0	26
N	3	0	10
O	2	0	8

Elmt	Val.	Min	Max
F	1	0	0
S	2	0	5
Cl	1	0	0

Use Adduct
H
Na
K
NH4

Error Margin (ppm):	20	DBE Range:	-100.0 - 200.0	Electron Ions:	odd
HC Ratio:	0.0 - 100.0	Apply N Rule:	yes	Use MSn Info:	no
Max Isotopes:	all	Isotope RI (%):	1.00	Isotope Res:	10000
MSn Iso RI (%):	75.00	MSn Logic Mode:	OR	Max Results:	1000

Event#: 1 MS(E+) Ret. Time : 1.357 Scan# : 203

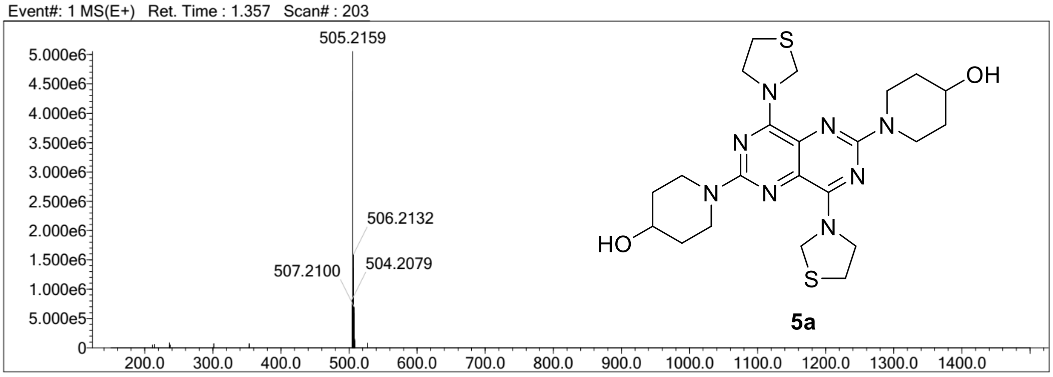

Measured region for 505.2159 m/z

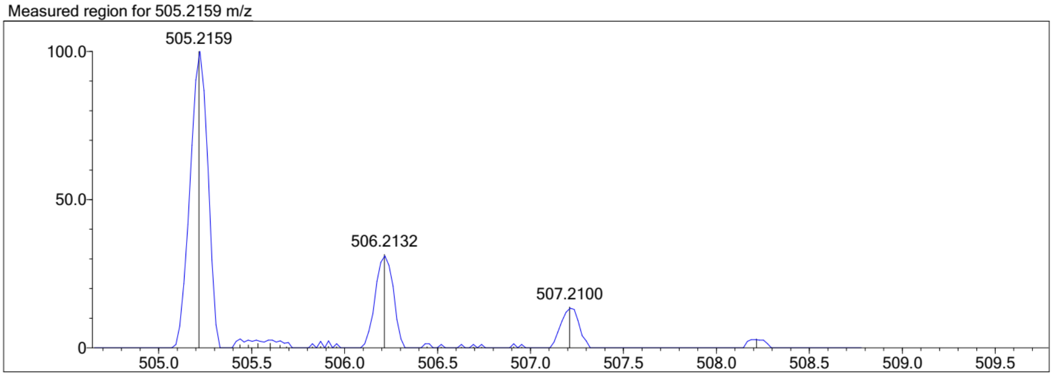

C22 H32 N8 O2 S2 [M+H]+ : Predicted region for 505.2162 m/z

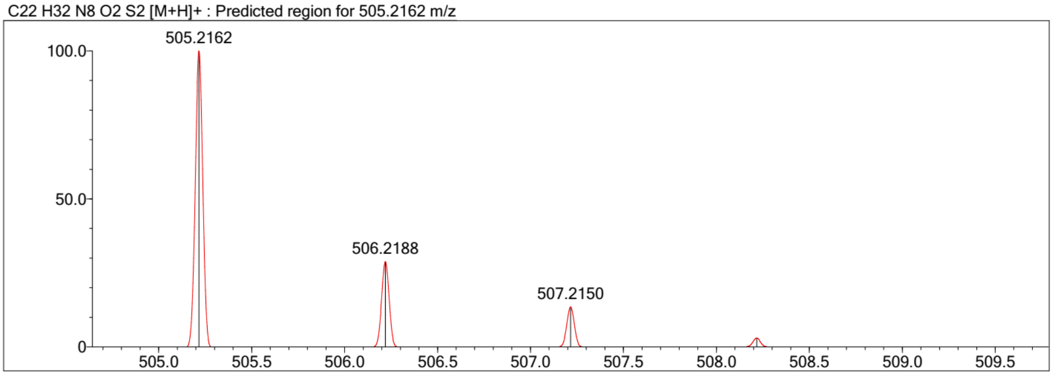

Rank	Score	Formula (M)	Ion	Meas. m/z	Pred. m/z	Df. (mDa)	Df. (ppm)	Iso	DBE
1	96.28	C22 H32 N8 O2 S2	[M+H]+	505.2159	505.2162	-0.3	-0.59	96.28	11.0

Elmt	Val.	Min	Max
H	1	0	45
C	4	0	26
N	3	0	10
O	2	0	8

Elmt	Val.	Min	Max
F	1	0	0
S	2	0	5
Cl	1	0	0

Use Adduct
H
Na
K
NH4

Error Margin (ppm):	20	DBE Range:	-100.0 - 200.0	Electron Ions:	odd
HC Ratio:	0.0 - 100.0	Apply N Rule:	yes	Use MSn Info:	no
Max Isotopes:	all	Isotope RI (%):	1.00	Isotope Res:	10000
MSn Iso RI (%):	75.00	MSn Logic Mode:	OR	Max Results:	1000

Event#: 1 MS(E+) Ret. Time : 1.463 Scan# : 219

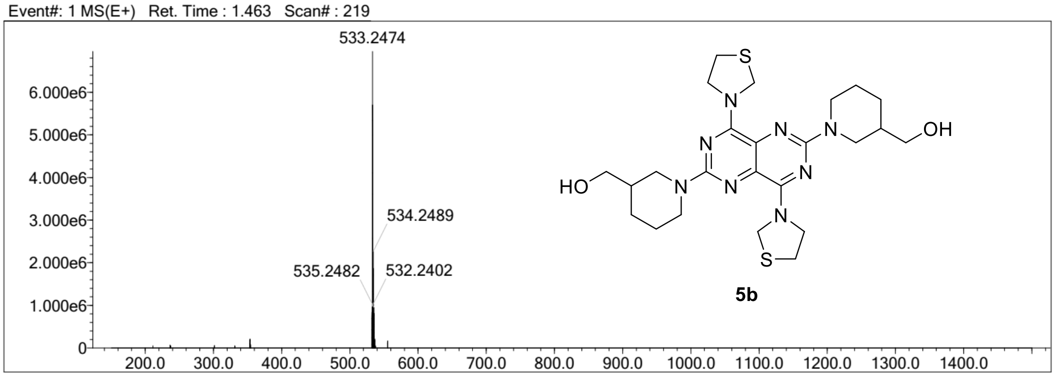

Measured region for 533.2474 m/z

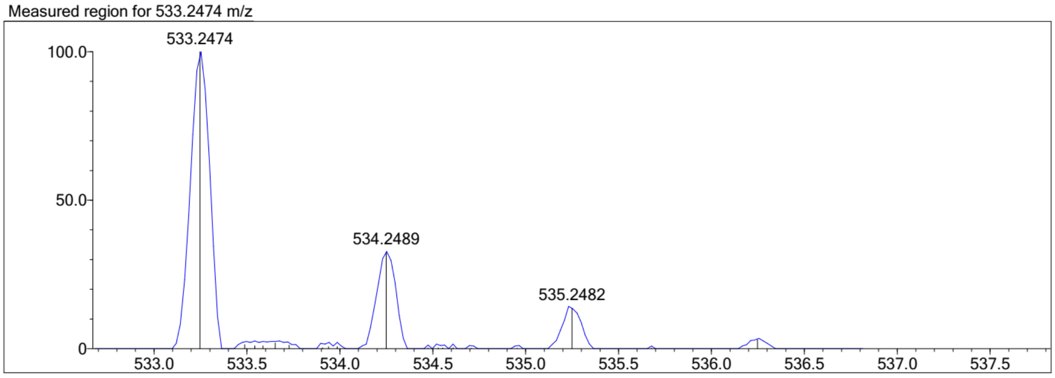

C24 H36 N8 O2 S2 [M+H]+ : Predicted region for 533.2475 m/z

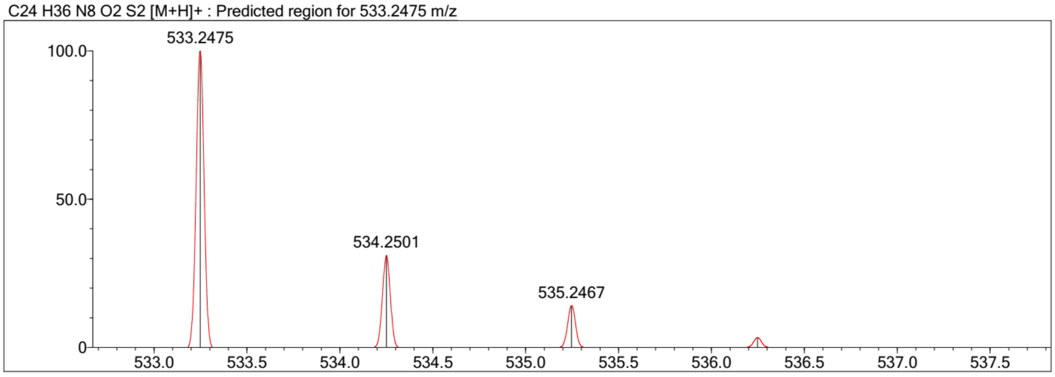

Rank	Score	Formula (M)	Ion	Meas. m/z	Pred. m/z	Df. (mDa)	Df. (ppm)	Iso	DBE
1	86.02	C24 H36 N8 O2 S2	[M+H]+	533.2474	533.2475	-0.1	-0.19	86.02	11.0

Elmt	Val.	Min	Max	Elmt	Val.	Min	Max	Elmt	Val.	Min	Max	Use Adduct
H	1	13	55	O	2	0	8	Br	1	0	0	H
B	3	0	0	F	1	0	0					Na
C	4	14	45	S	2	0	4					K
N	3	0	10	Cl	1	0	0					NH4

Error Margin (ppm):	100	DBE Range:	not fixed	Electron Ions:	both
HC Ratio:	unlimited	Apply N Rule:	no	Use MSn Info:	no
Max Isotopes:	all	Isotope RI (%):	1.00	Isotope Res:	10000
MSn Iso RI (%):	75.00	MSn Logic Mode:	AND	Max Results:	100

Event#: 1 MS(E+) Ret. Time : 1.503 Scan# : 225

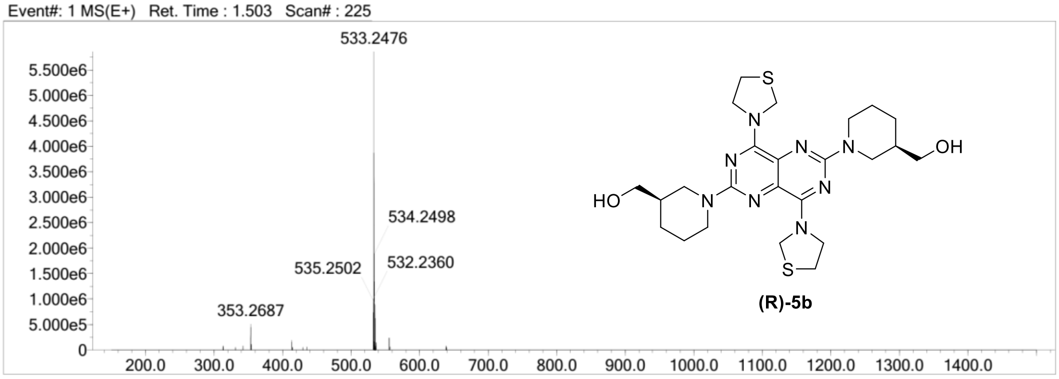

Measured region for 533.2476 m/z

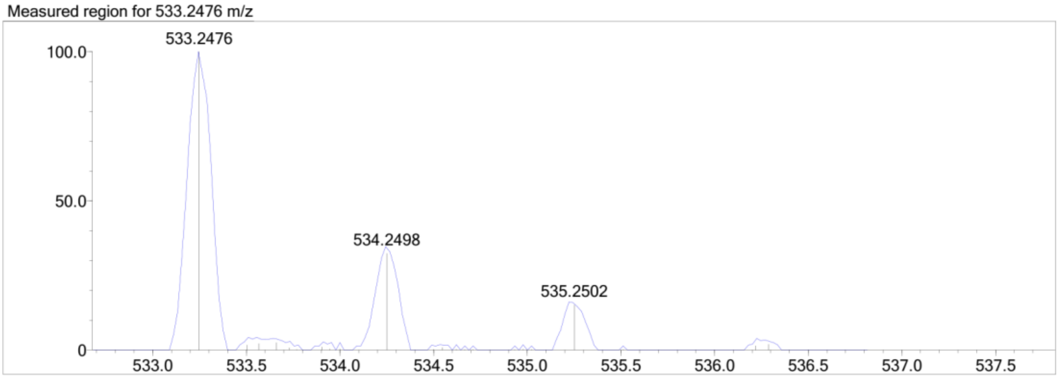

C24 H36 N8 O2 S2 [M+H]+ : Predicted region for 533.2475 m/z

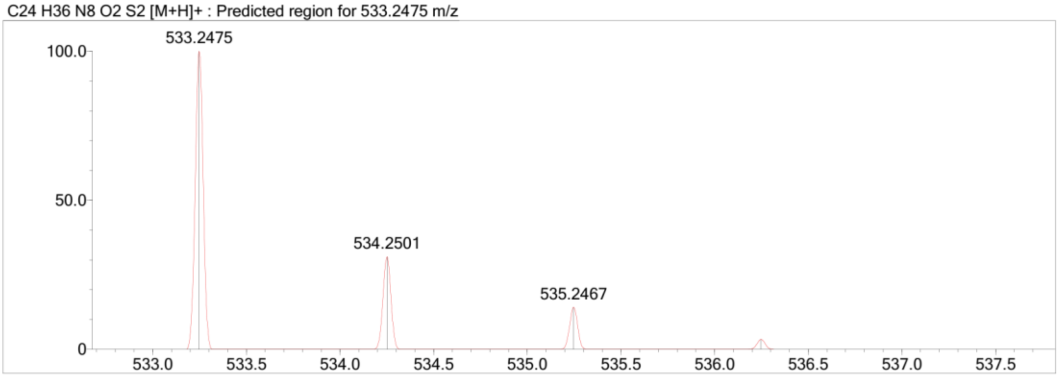

Rank	Score	Formula (M)	Ion	Meas. m/z	Pred. m/z	Df. (mDa)	Df. (ppm)	Iso	DBE
1	87.58	C24 H36 N8 O2 S2	[M+H]+	533.2476	533.2475	0.1	0.19	87.58	11.0

Elmt	Val.	Min	Max	Elmt	Val.	Min	Max	Elmt	Val.	Min	Max	Use Adduct
H	1	13	40	O	2	0	8	Br	1	0	0	H
B	3	0	0	F	1	0	0					Na
C	4	14	45	S	2	0	4					K
N	3	6	10	Cl	1	0	0					NH4

Error Margin (ppm):	100	DBE Range:	not fixed	Electron Ions:	both
HC Ratio:	unlimited	Apply N Rule:	no	Use MSn Info:	no
Max Isotopes:	all	Isotope RI (%):	1.00	Isotope Res:	10000
MSn Iso RI (%):	75.00	MSn Logic Mode:	AND	Max Results:	100

Event#: 1 MS(E+) Ret. Time : 1.397 Scan# : 209

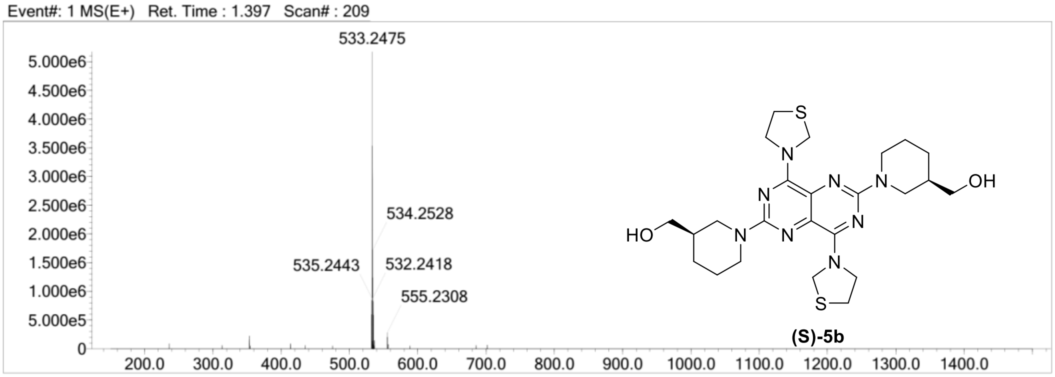

Measured region for 533.2475 m/z

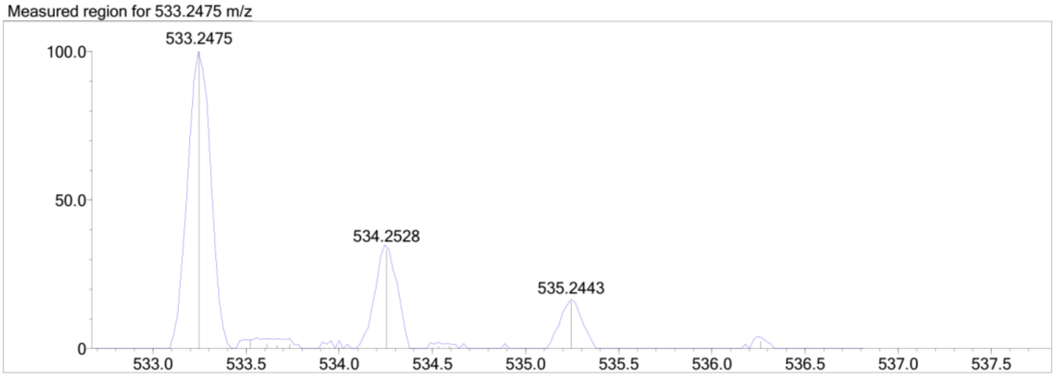

C24 H36 N8 O2 S2 [M+H]+ : Predicted region for 533.2475 m/z

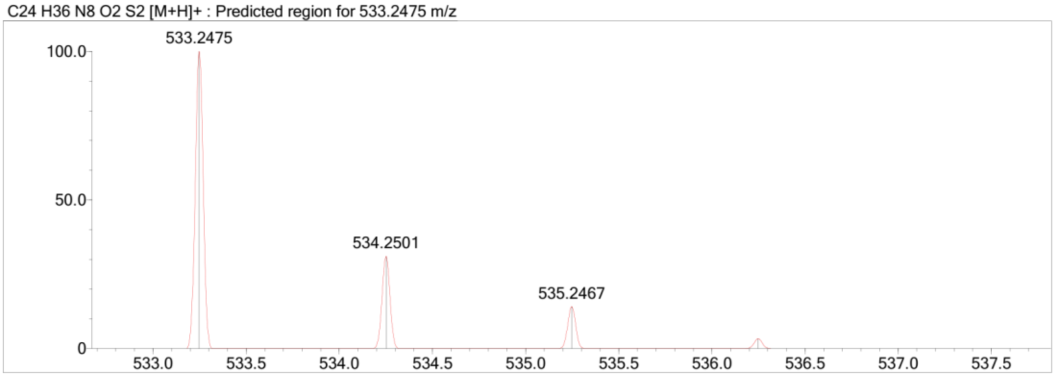

Rank	Score	Formula (M)	Ion	Meas. m/z	Pred. m/z	Df. (mDa)	Df. (ppm)	Iso	DBE
1	80.25	C24 H36 N8 O2 S2	[M+H]+	533.2475	533.2475	-0.0	0.00	80.25	11.0

Elmt	Val.	Min	Max
H	1	0	45
C	4	0	26
N	3	0	10
O	2	0	8

Elmt	Val.	Min	Max
F	1	0	0
S	2	0	5
Cl	1	0	0

Use Adduct
H
Na
K
NH4

Error Margin (ppm):	20	DBE Range:	-100.0 - 200.0	Electron Ions:	odd
HC Ratio:	0.0 - 100.0	Apply N Rule:	yes	Use MSn Info:	no
Max Isotopes:	all	Isotope RI (%):	1.00	Isotope Res:	10000
MSn Iso RI (%):	75.00	MSn Logic Mode:	OR	Max Results:	1000

Event#: 1 MS(E+) Ret. Time : 1.210 Scan# : 181

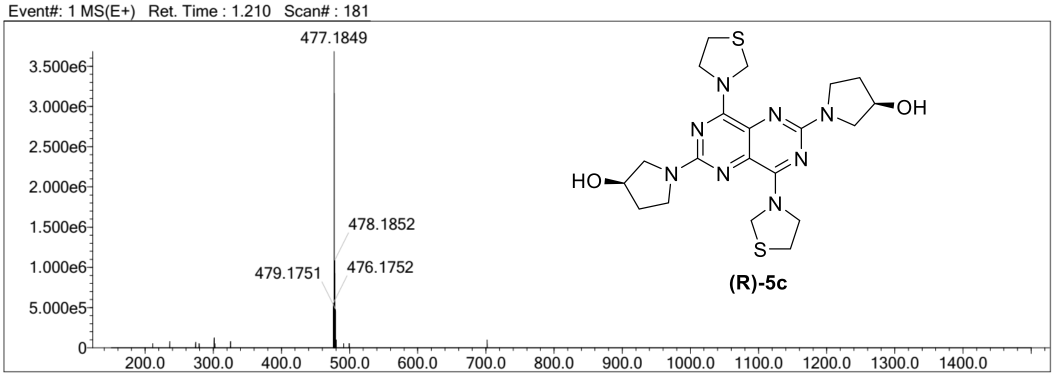

Measured region for 477.1849 m/z

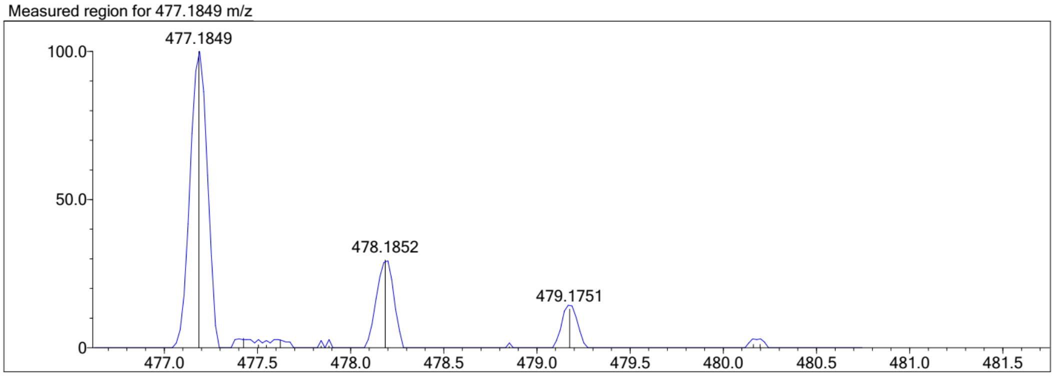

C20 H28 N8 O2 S2 [M+H]+ : Predicted region for 477.1849 m/z

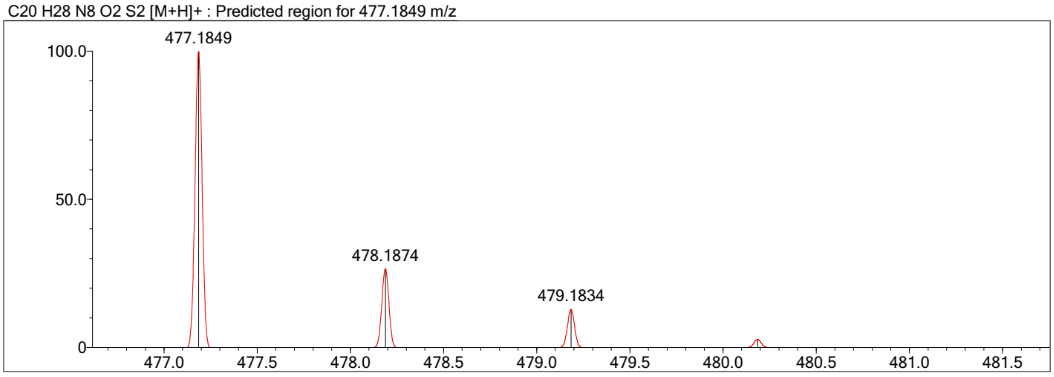

Rank	Score	Formula (M)	Ion	Meas. m/z	Pred. m/z	Df. (mDa)	Df. (ppm)	Iso	DBE
1	90.07	C20 H28 N8 O2 S2	[M+H]+	477.1849	477.1849	-0.0	0.00	90.07	11.0

Elmt	Val.	Min	Max
H	1	0	45
C	4	0	26
N	3	0	10
O	2	0	8

Elmt	Val.	Min	Max
F	1	0	0
S	2	0	5
Cl	1	0	0

Use Adduct
H
Na
K
NH4

Error Margin (ppm):	20	DBE Range:	-100.0 - 200.0	Electron Ions:	odd
HC Ratio:	0.0 - 100.0	Apply N Rule:	yes	Use MSn Info:	no
Max Isotopes:	all	Isotope RI (%):	1.00	Isotope Res:	10000
MSn Iso RI (%):	75.00	MSn Logic Mode:	OR	Max Results:	1000

Event#: 1 MS(E+) Ret. Time : 0.943 Scan# : 141

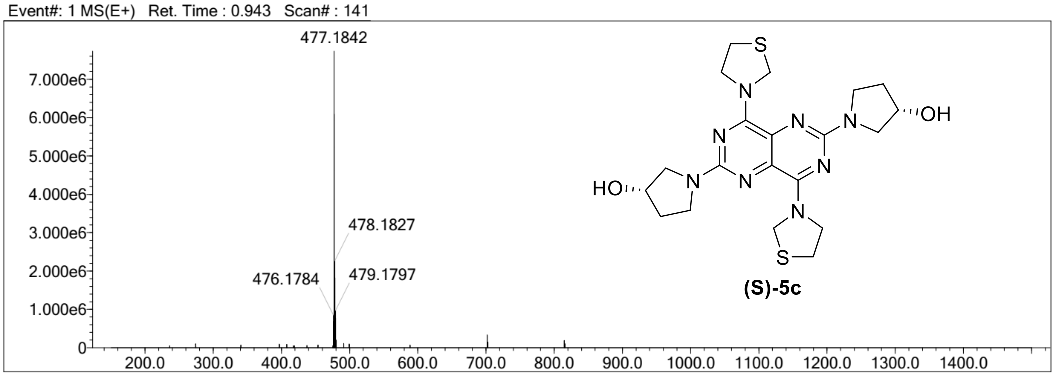

Measured region for 477.1842 m/z

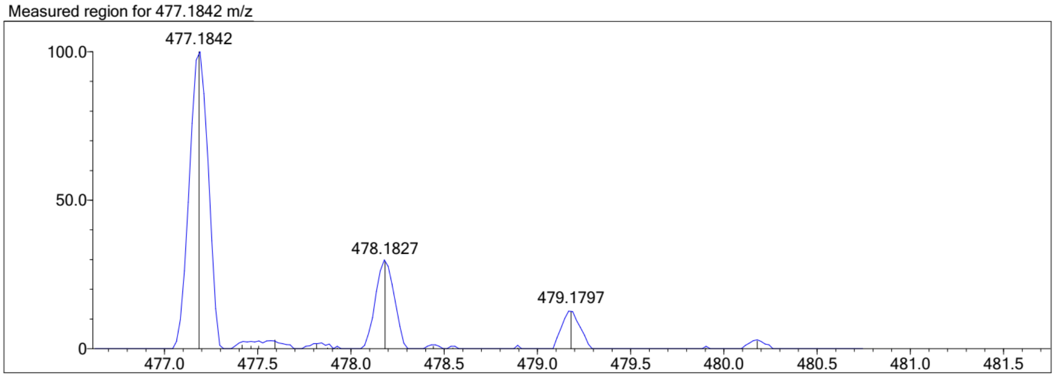

C20 H28 N8 O2 S2 [M+H]+ : Predicted region for 477.1849 m/z

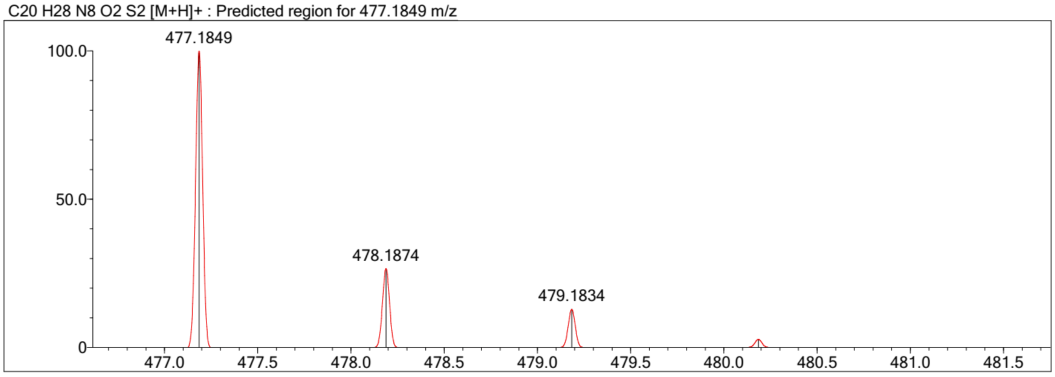

Rank	Score	Formula (M)	Ion	Meas. m/z	Pred. m/z	Df. (mDa)	Df. (ppm)	Iso	DBE
1	82.75	C20 H28 N8 O2 S2	[M+H]+	477.1842	477.1849	-0.7	-1.47	83.73	11.0

Elmt	Val.	Min	Max	Elmt	Val.	Min	Max	Use Adduct
H	1	0	45	F	1	0	4	H
C	4	0	35	S	2	0	0	Na
N	3	0	10	Cl	1	0	0	K
O	2	0	8					NH4

Error Margin (ppm):	20	DBE Range:	-100.0 - 200.0	Electron Ions:	odd
HC Ratio:	0.0 - 100.0	Apply N Rule:	yes	Use MSn Info:	no
Max Isotopes:	all	Isotope RI (%):	1.00	Isotope Res:	10000
MSn Iso RI (%):	75.00	MSn Logic Mode:	OR	Max Results:	1000

Event#: 1 MS(E+) Ret. Time : 1.130 Scan# : 169

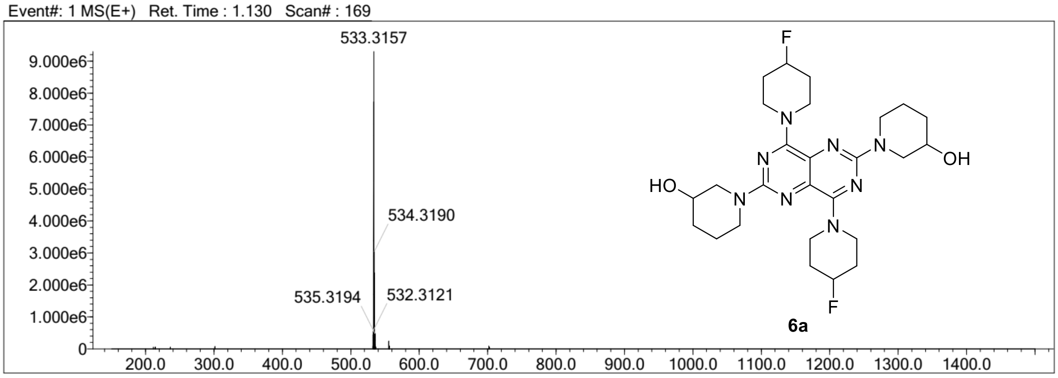

Measured region for 533.3157 m/z

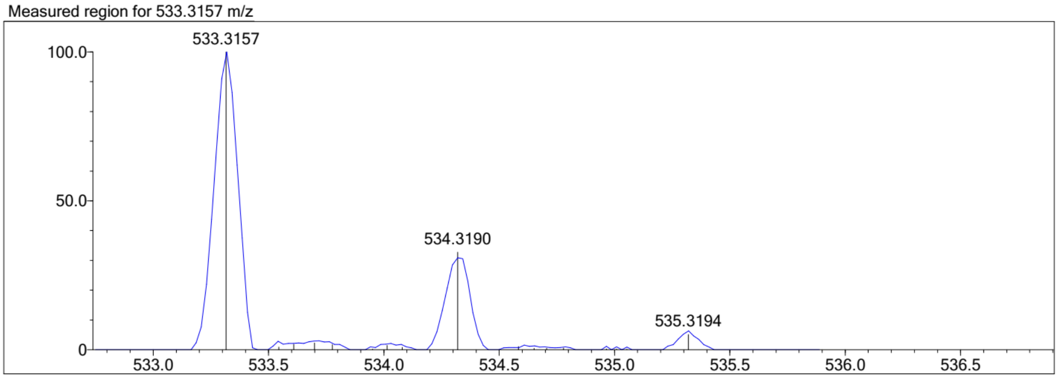

C26 H38 N8 O2 F2 [M+H]+ : Predicted region for 533.3159 m/z

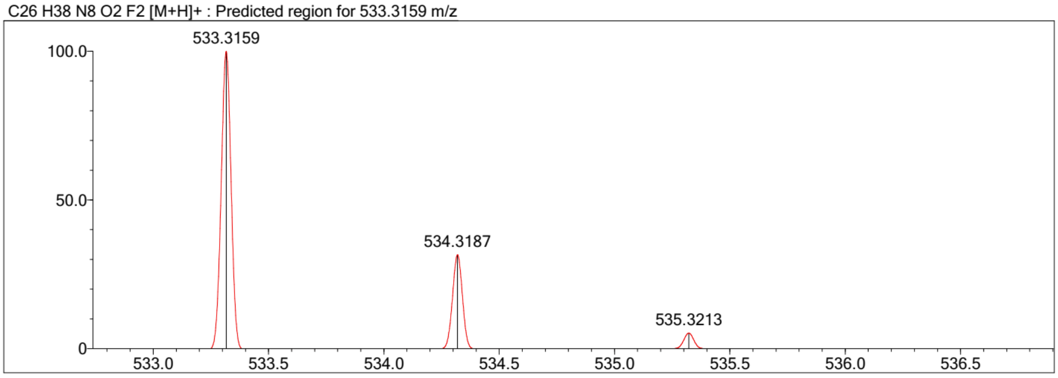

Rank	Score	Formula (M)	Ion	Meas. m/z	Pred. m/z	Df. (mDa)	Df. (ppm)	Iso	DBE
1	87.61	C26 H38 N8 O2 F2	[M+H]+	533.3157	533.3159	-0.2	-0.38	87.61	11.0

Elmt	Val.	Min	Max
H	1	0	40
B	3	0	0
C	4	10	27
N	3	0	10

Elmt	Val.	Min	Max
O	2	1	4
F	1	1	5
S	2	0	0
Cl	1	0	0

Elmt	Val.	Min	Max
K	1	0	0
Br	1	0	0

Use Adduct
H
Na
K
NH4

Error Margin (ppm): 100
HC Ratio: unlimited
Max Isotopes: all
MSn Iso RI (%): 75.00

DBE Range: not fixed
Apply N Rule: no
Isotope RI (%): 1.00
MSn Logic Mode: AND

Electron Ions: both
Use MSn Info: no
Isotope Res: 10000
Max Results: 100

Event#: 1 MS(E+) Ret. Time : 1.050 Scan# : 157

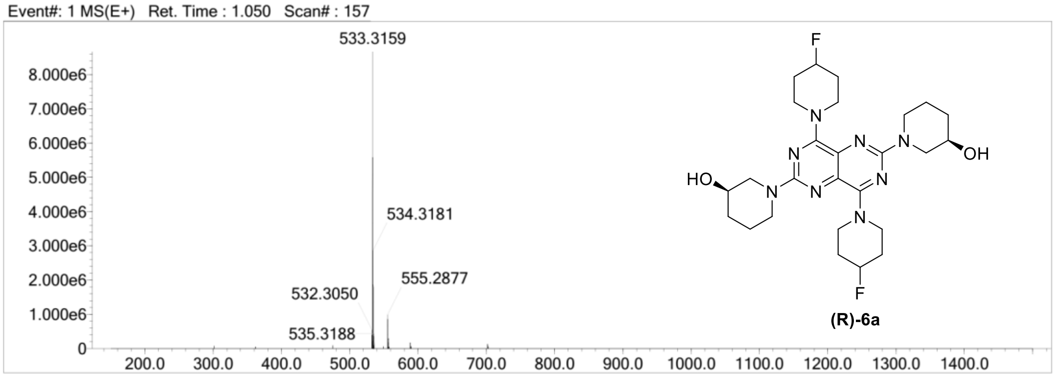

Measured region for 533.3159 m/z

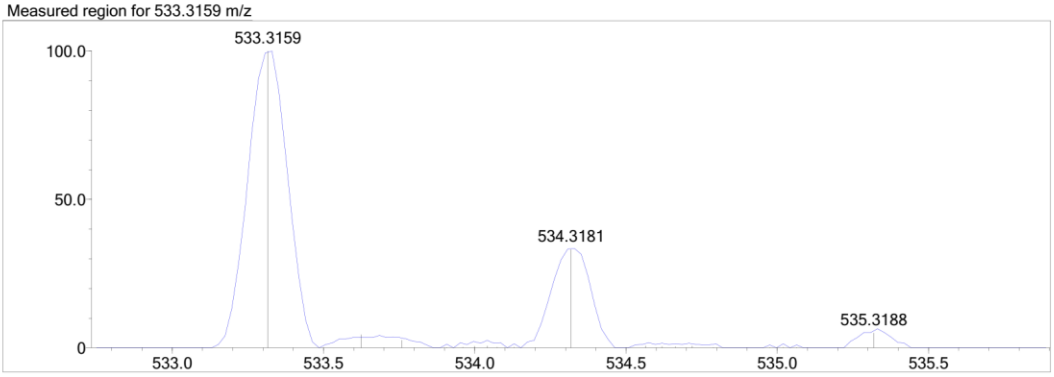

C26 H38 N8 O2 F2 [M+H]+ : Predicted region for 533.3159 m/z

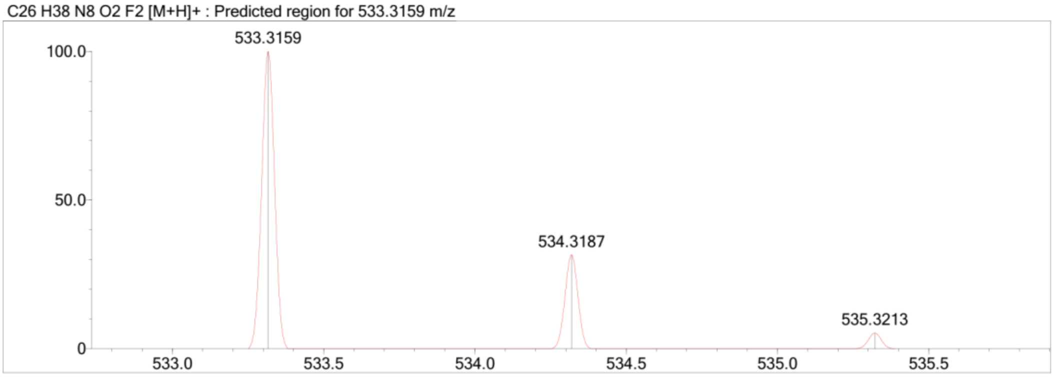

Rank	Score	Formula (M)	Ion	Meas. m/z	Pred. m/z	Df. (mDa)	Df. (ppm)	Iso	DBE
1	82.79	C26 H38 N8 O2 F2	[M+H]+	533.3159	533.3159	0.0	0.00	82.79	11.0

Elmt	Val.	Min	Max	Elmt	Val.	Min	Max	Use Adduct
H	1	0	45	F	1	0	4	H
C	4	0	35	S	2	0	0	Na
N	3	0	10	Cl	1	0	0	K
O	2	0	8					NH4

Error Margin (ppm):	20	DBE Range:	-100.0 - 200.0	Electron Ions:	odd
HC Ratio:	0.0 - 100.0	Apply N Rule:	yes	Use MSn Info:	no
Max Isotopes:	all	Isotope RI (%):	1.00	Isotope Res:	10000
MSn Iso RI (%):	75.00	MSn Logic Mode:	OR	Max Results:	1000

Event#: 1 MS(E+) Ret. Time : 0.717 Scan# : 107

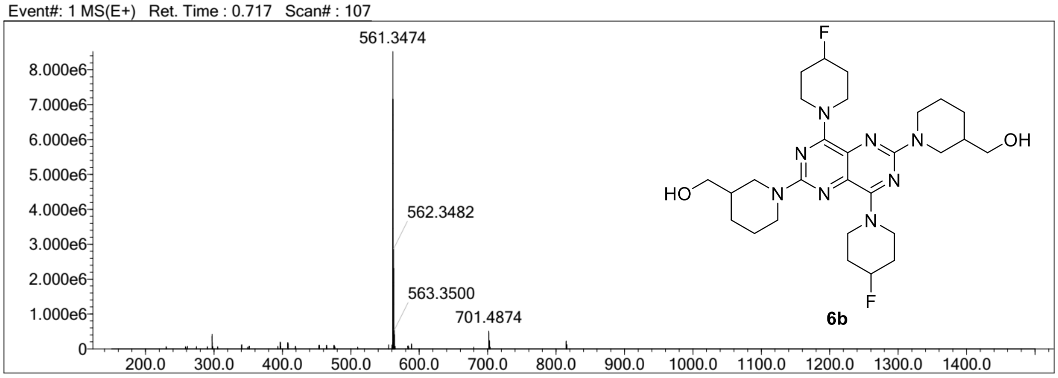

Measured region for 561.3474 m/z

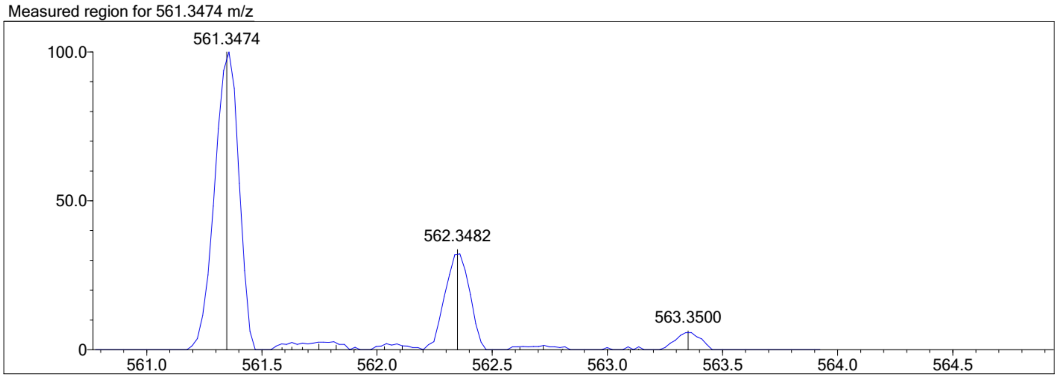

C28 H42 N8 O2 F2 [M+H]+ : Predicted region for 561.3472 m/z

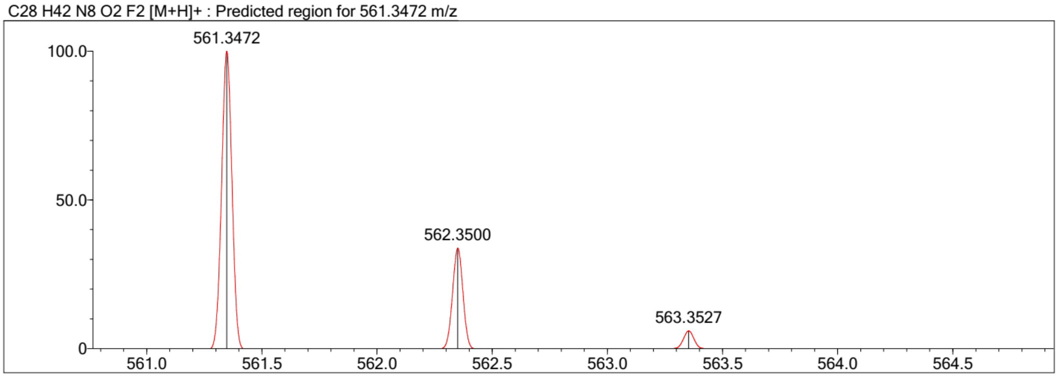

Rank	Score	Formula (M)	Ion	Meas. m/z	Pred. m/z	Df. (mDa)	Df. (ppm)	Iso	DBE
1	83.70	C28 H42 N8 O2 F2	[M+H]+	561.3474	561.3472	0.2	0.36	83.70	11.0

Elmt	Val.	Min	Max
H	1	0	45
C	4	0	35
N	3	0	10
O	2	0	8

Elmt	Val.	Min	Max
F	1	0	4
S	2	0	0
Cl	1	0	0

Use Adduct
H
Na
K
NH4

Error Margin (ppm):	20	DBE Range:	-100.0 - 200.0	Electron Ions:	odd
HC Ratio:	0.0 - 100.0	Apply N Rule:	yes	Use MSn Info:	no
Max Isotopes:	all	Isotope RI (%):	1.00	Isotope Res:	10000
MSn Iso RI (%):	75.00	MSn Logic Mode:	OR	Max Results:	1000

Event#: 1 MS(E+) Ret. Time : 1.157 Scan# : 173

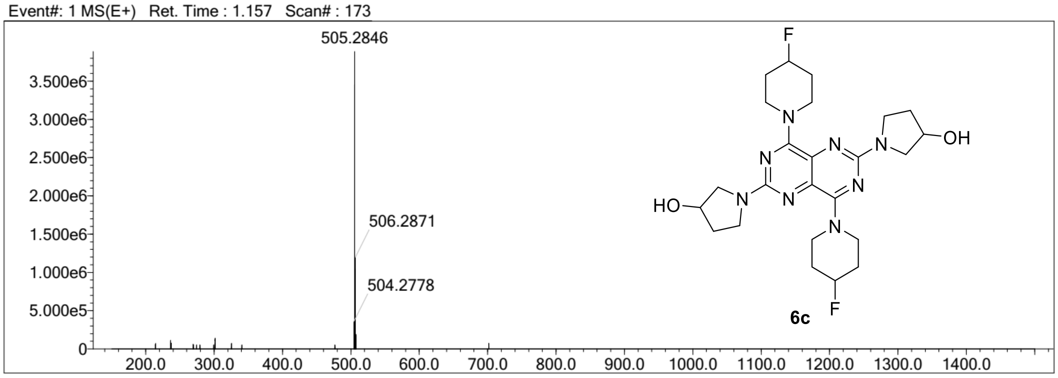

Measured region for 505.2846 m/z

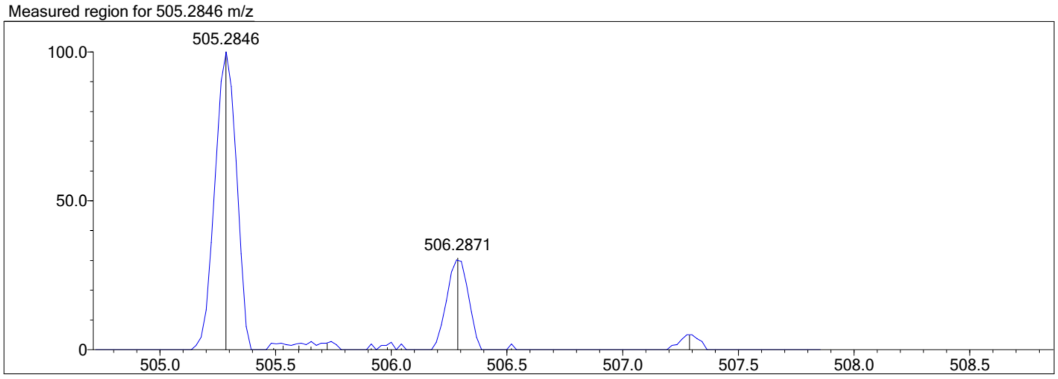

C24 H34 N8 O2 F2 [M+H]+ : Predicted region for 505.2846 m/z

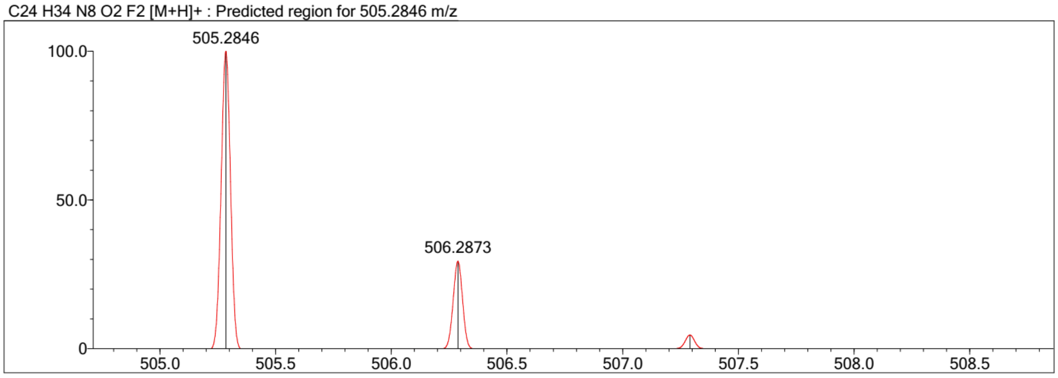

Rank	Score	Formula (M)	Ion	Meas. m/z	Pred. m/z	Df. (mDa)	Df. (ppm)	Iso	DBE
1	85.11	C24 H34 N8 O2 F2	[M+H]+	505.2846	505.2846	0.0	0.00	85.11	11.0

Elmt	Val.	Min	Max	Elmt	Val.	Min	Max	Elmt	Val.	Min	Max	Use Adduct
H	1	0	50	O	2	0	10	Cl	1	0	0	H
C	4	0	32	F	1	0	0	Br	1	0	0	Na
N	3	0	10	S	2	1	4					

Error Margin (ppm):	100	DBE Range:	not fixed	Electron Ions:	both
HC Ratio:	unlimited	Apply N Rule:	no	Use MSn Info:	no
Max Isotopes:	all	Isotope RI (%):	1.00	Isotope Res:	10000
MSn Iso RI (%):	75.00	MSn Logic Mode:	AND	Max Results:	100

Event#: 1 MS(E+) Ret. Time : 0.690 Scan# : 103

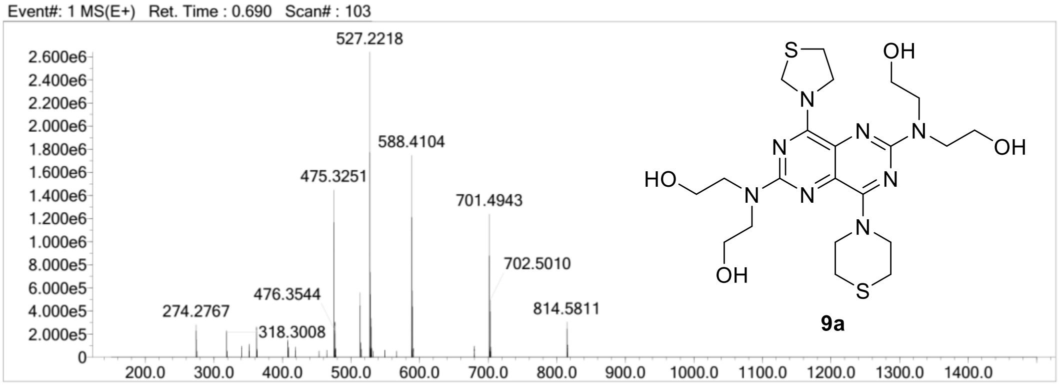

9a

Measured region for 527.2218 m/z

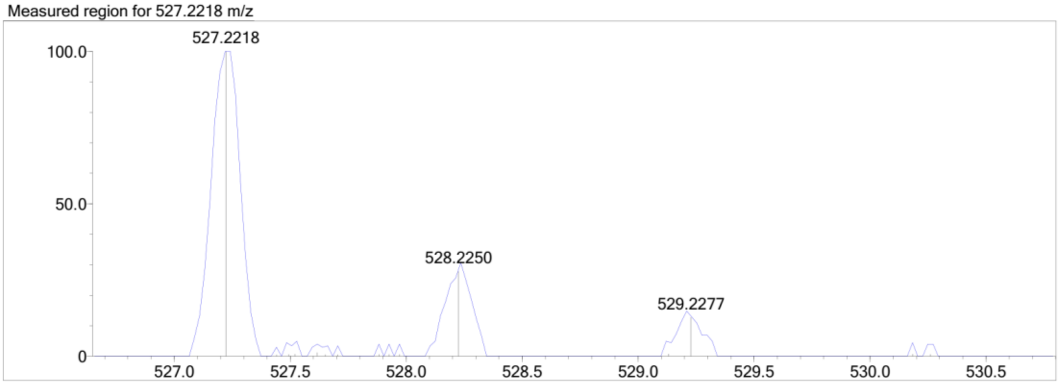

C21 H34 N8 O4 S2 [M+H]+ : Predicted region for 527.2217 m/z

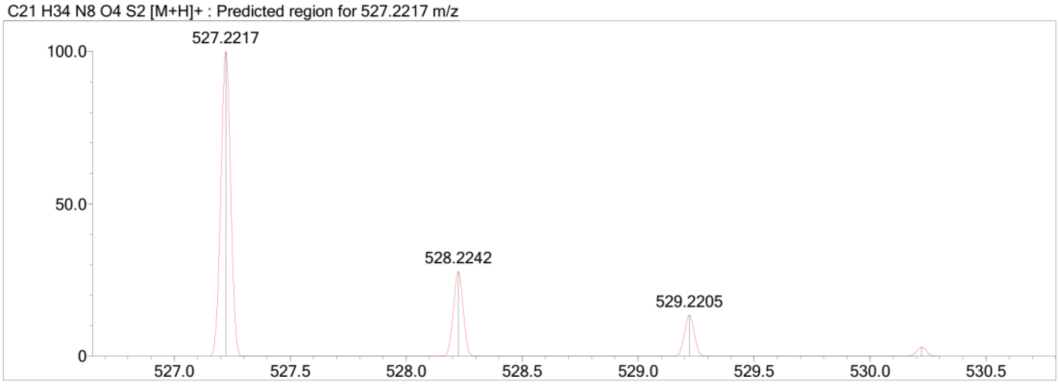

Rank	Score	Formula (M)	Ion	Meas. m/z	Pred. m/z	Df. (mDa)	Df. (ppm)	Iso	DBE
1	85.64	C21 H34 N8 O4 S2	[M+H]+	527.2218	527.2217	0.1	0.19	85.64	9.0

Elmt	Val.	Min	Max	Elmt	Val.	Min	Max	Elmt	Val.	Min	Max	Use Adduct
H	1	0	50	O	2	0	8	Cl	1	0	0	H
C	4	20	32	F	1	1	4	Br	1	0	0	Na
N	3	0	10	S	2	1	4					

Error Margin (ppm):	100	DBE Range:	not fixed	Electron Ions:	both
HC Ratio:	unlimited	Apply N Rule:	no	Use MSn Info:	no
Max Isotopes:	all	Isotope RI (%):	1.00	Isotope Res:	10000
MSn Iso RI (%):	75.00	MSn Logic Mode:	AND	Max Results:	100

Event#: 1 MS(E+) Ret. Time : 0.623 Scan# : 93

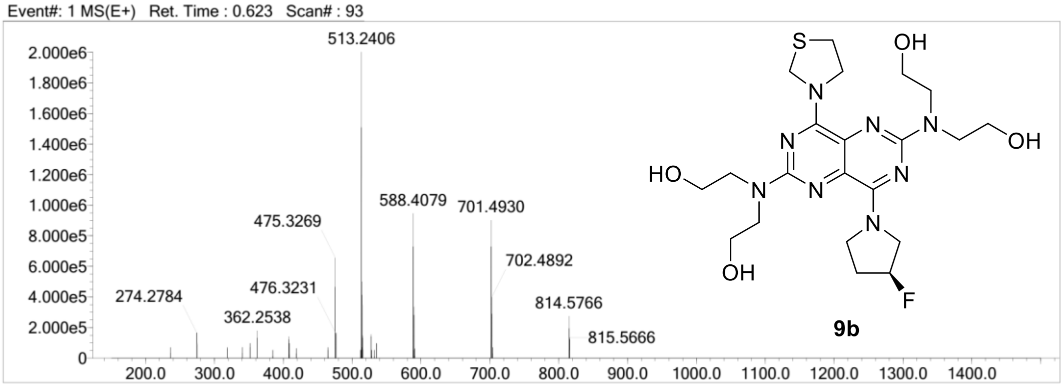

Measured region for 513.2406 m/z

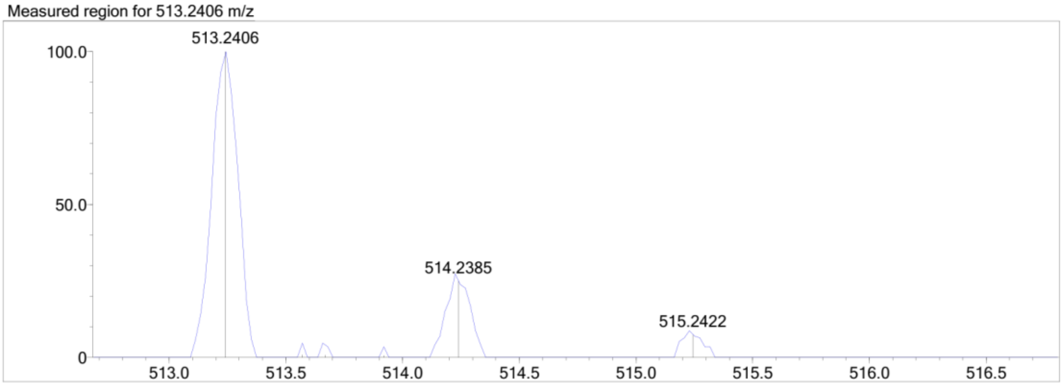

C21 H33 N8 O4 F S [M+H]+ : Predicted region for 513.2402 m/z

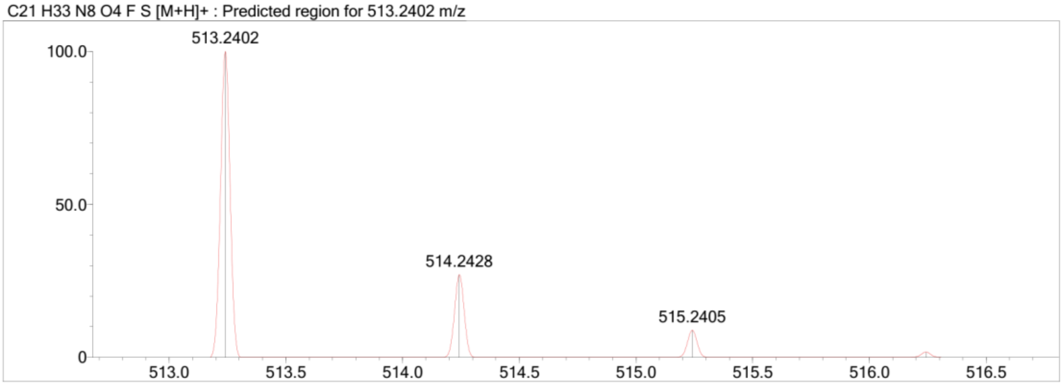

Rank	Score	Formula (M)	Ion	Meas. m/z	Pred. m/z	Df. (mDa)	Df. (ppm)	Iso	DBE
1	83.10	C21 H33 N8 O4 F S	[M+H]+	513.2406	513.2402	0.4	0.78	83.10	9.0
